# Exhaustive Genome-Wide Search for SNP-SNP Interactions Across 10 Human Diseases

**DOI:** 10.1534/g3.116.028563

**Published:** 2016-05-12

**Authors:** William Murk, Andrew T. DeWan

**Affiliations:** Department of Chronic Disease Epidemiology, Yale School of Public Health, New Haven, Connecticut 06510

**Keywords:** epistasis, statistical interaction, GERA, gene-gene interaction

## Abstract

The identification of statistical SNP-SNP interactions may help explain the genetic etiology of many human diseases, but exhaustive genome-wide searches for these interactions have been difficult, due to a lack of power in most datasets. We aimed to use data from the Resource for Genetic Epidemiology Research on Adult Health and Aging (GERA) study to search for SNP-SNP interactions associated with 10 common diseases. FastEpistasis and BOOST were used to evaluate all pairwise interactions among approximately *N* = 300,000 single nucleotide polymorphisms (SNPs) with minor allele frequency (MAF) ≥ 0.15, for the dichotomous outcomes of allergic rhinitis, asthma, cardiac disease, depression, dermatophytosis, type 2 diabetes, dyslipidemia, hemorrhoids, hypertensive disease, and osteoarthritis. A total of *N* = 45,171 subjects were included after quality control steps were applied. These data were divided into discovery and replication subsets; the discovery subset had > 80% power, under selected models, to detect genome-wide significant interactions (*P* < 10^−12^). Interactions were also evaluated for enrichment in particular SNP features, including functionality, prior disease relevancy, and marginal effects. No interaction in any disease was significant in both the discovery and replication subsets. Enrichment analysis suggested that, for some outcomes, interactions involving SNPs with marginal effects were more likely to be nominally replicated, compared to interactions without marginal effects. If SNP-SNP interactions play a role in the etiology of the studied conditions, they likely have weak effect sizes, involve lower-frequency variants, and/or involve complex models of interaction that are not captured well by the methods that were utilized.

Gene-gene interaction, also known as epistasis, is the phenomenon where the phenotypic effect of variation at one genetic locus depends on variation at other loci (Cordell 2002). Epistasis is thought to be biologically prevalent and to contribute to the missing heritability problem for complex human disease (Lunzer *et al.* 2010; Huang *et al.* 2012; Zuk *et al.* 2012; Hemani *et al.* 2013). As a result, a significant amount of effort has been invested into attempting to identify epistatic effects in human traits (Hemani *et al.* 2014; Wei *et al.* 2014; Murk *et al.* 2015). The study of epistasis is challenged by its high-dimensional nature, which on a genome-wide scale can result in a very large search space and significant difficulties in attaining sufficient statistical power to detect effects. To overcome this challenge, most studies of epistasis have restricted their search space to relatively small numbers of single nucleotide polymorphisms (SNPs), often focusing on those located in previously identified trait-relevant candidate genes (Murk *et al.* 2015). However, this type of approach will exclude the vast majority of the genetic search space and thus potentially miss a large number of interacting genes.

We aimed to conduct well-powered, genome-wide searches for epistasis at the pairwise SNP level (*i.e.*, to identify SNP-SNP interactions), using data from the GERA study (Hoffmann *et al.* 2011a,b). This study involved a large number of subjects (*N* = 78,486 initially available) who were genotyped using genome-wide SNP arrays, and who had health outcome data available for a number of electronic medical records-derived medical conditions. In addition, we sought to identify SNP characteristics that were enriched among replicated interactions, which could inform strategies to reduce the search space in smaller datasets.

To perform the exhaustive searches for interaction, two different analytical approaches were used: FastEpistasis and BOOST. FastEpistasis is a computationally efficient but imprecise method to screen for interactions, and is intended to be used in conjunction with follow-up analysis by slower but more precise methods, such as logistic regression (Purcell *et al.* 2007; Ueki and Cordell 2012). BOOST is another computationally efficient method that has been found to have greater power than FastEpistasis for some models of interaction, particularly models that do not have main effects (Wan *et al.* 2010). The use of these two different methods could therefore allow greater flexibility in detecting multiple different forms of interaction.

## Materials and Methods

### Study dataset

Authorized access to data from the GERA study was obtained (dbGaP Study Accession: phs000674.v1.p1), and use of these data was approved by the Yale Human Investigation Committee. This study is described in detail elsewhere (http://www.ncbi.nlm.nih.gov/projects/gap/cgi-bin/study.cgi?study_id=phs000674.v1.p1) (Hoffmann *et al.* 2011a,b; Kvale *et al.* 2015). Briefly, the study was comprised of 78,486 subjects who were members of the Kaiser Permanente Medical Care Plan, Northern California Region, and who participated in the Kaiser Permanente Research Program on Genes, Environment, and Health (RPGEH) survey study. All subjects were 18 or more years of age at the start of the RPGEH survey in 2007, were members of the aforementioned medical care plan for at least 2 yr prior, and provided broad consent for the use of their data in research. Genotype data were derived from custom-designed Affymetrix Axiom genome-wide SNP microarrays (Hoffmann *et al.* 2011a,b). Four different chips were designed, and subjects were assigned to a particular chip depending on his or her race/ethnicity categorization (http://www.ncbi.nlm.nih.gov/projects/gap/cgi-bin/GetPdf.cgi?id=phd004309). In the present study, we included all subjects who were genotyped on the EUR (“Non-Hispanic White”) chip (counts before quality control: *N* = 62,318 subjects and *N* = 670,176 SNPs available).

Health outcomes were binary-coded (yes or no) variables representing various medical conditions, as derived from Kaiser Permanente Electronic Medical Records. These conditions were defined based on a subject having at least two medical diagnoses within an ICD-9-based disease category. These conditions and the ICD-9 codes that were used to define them are listed here: http://www.ncbi.nlm.nih.gov/projects/gap/cgi-bin/GetPdf.cgi?id=phd004308. Individual-level diagnostic codes were not available (*i.e.*, only the derived binary variables were present in the dataset). We excluded from potential analysis the conditions “Cancer” and “Psychiatric,” as it was judged that these conditions were too broadly defined for the objectives of the present study. We also excluded any conditions for which we had less than 50% power to detect an interaction (based on Quanto-derived estimations to detect an interaction OR ≥ 1.50, at an α of 10^−12^; see power estimation methods below). After these exclusions, the conditions “asthma,” “allergic rhinitis,” “cardiac disease,” “depression,” “dermatophytosis,” “diabetes, type 2,” “dyslipidemia,” “hypertensive disease,” “hemorrhoids,” and “osteoarthritis” (*N* = 10 conditions) were included as outcomes for analysis.

Additional variables included from the dataset included birth year category, sex, and genetic structure principal components (10 components total). The principal components were previously calculated and provided by the original GERA study investigators; details of this are reported here: http://www.ncbi.nlm.nih.gov/projects/gap/cgi-bin/GetPdf.cgi?id=phd004309. Birth year category was defined as one of 14 possible time periods (category 1: birth year ≤ 1923; categories 2–14: 5-year categories, starting with 2 being the period 1924–1928). We defined “minimum age” as the difference in years between the time of survey (2007) and the last year of the birth year category. For example, the minimum age for a subject with a birth year category of 2 was defined to be 2007 − 1928 = 79 years.

### Sample quality control (QC)

Subjects were excluded if they (i) had an ambiguous SNP-estimated sex; (ii) had a subject call rate < 95%; (iii) were selected for exclusion due to relatedness with other subjects; (iv) had a reported race/ethnicity other than “white” or did not have principal components available for the EUR chip; and (v) were a PCA outlier, defined as having a value > 6 standard deviations on any one of the 10 principal components. Ambiguous SNP-estimated sex was defined as having an X-chromosome homozygosity estimate (F) between 0.2–0.8. There were 17 subjects for whom the unambiguous SNP-estimated sex differed from the reported sex; in such cases, the SNP-estimated sex was considered to be the true sex. To assess subject relatedness, pi-hat (proportion identical by descent) was estimated using the dataset after being pruned for linkage disequilibrium (LD) (*N* = 358,590 SNPs). Pi-hat was calculated for every possible pair of subjects; for pairs with pi-hat ≥ 0.20, one subject of the pair was randomly excluded. The numbers of subjects excluded at each QC step are listed in Supplemental Material, Table S1.

### Case/control definitions

Cases and controls were defined after sample quality control procedures were completed. For each of the 10 outcomes included for analysis, cases were subjects who were coded as having the respective condition, while controls were subjects without it. Controls were excluded if their birth year category exceeded the 99.5 percentile birth year category of cases (except in the case of asthma, allergic rhinitis, and dermatophytosis). For example, for osteoarthritis cases, the 99.5 percentile birth year category was 10 (minimum age of this category: 39 years); therefore, all controls with a birth year category of 11 or higher (*i.e.*, those younger than 39 years of age) were excluded. This was performed in order to exclude controls that were not of a sufficient age to be of comparable risk for the condition. No exclusions based on age were made for asthma, allergic rhinitis, and dermatophytosis, since these conditions are common in children. The number of controls excluded based on age are shown in Table S2. In the final analyses, across the different conditions, the numbers of cases ranged from 5549–24,047, and the numbers of controls ranged from 20,018–39,154.

### SNP quality control

Ten copies of the dataset, one for each included outcome condition, were created, and SNP QC procedures were performed within each condition-specific dataset. SNPs were excluded if they (i) could not be mapped to the hg19 reference genome; (ii) had a SNP call rate < 98% overall or in cases or controls separately; (iii) had a minor allele frequency (MAF) < 0.15 (this was specified because there was little power to detect an interaction involving SNPs that were less common than this); (iv) were nonautosomal; or (v) had a test for deviation from Hardy-Weinberg equilibrium with *P*-value < 10^−5^, among controls. The numbers of SNPs excluded at each QC step are listed in Table S3.

### Discovery and replication datasets

After sample and SNP QC, each outcome-specific dataset was randomly divided into a discovery and a replication dataset. These divisions were made such that the targeted size of each replication dataset was 1000 cases and 3000 controls. These numbers were chosen because they provided > 80% power for nominal replication (based on Quanto-derived estimations to detect an interaction OR ≥ 1.50, at an α of 0.05; see power estimation methods below). The final numbers of subjects in each discovery and replication dataset are shown in Table S2.

### Genomic inflation

At the level of marginal (individual-SNP) effects, test statistic inflation due to population stratification or other sources was assessed via calculations of the genomic inflation factor λ. Values of λ greater than 1.05 were considered evidence of test statistic inflation. Marginal test statistics were derived using genome-wide single-SNP logistic regression analyses performed within all 10 sets of discovery and replication datasets. The cardiac disease (λ = 1.14), dermatophytosis (λ = 1.23), type 2 diabetes (λ = 1.08), dyslipidemia (λ = 1.11), and hypertensive disease (λ = 1.30) data showed evidence of inflation (Table S4). For cardiac disease and dermatophytosis, this inflation was largely removed after adjusting for the first two principal components. For the other conditions, adjustment for the first two components either had no effect (type 2 diabetes) or reduced but did not remove the inflation [dyslipidemia (λ = 1.07) and hypertensive disease (λ=1.10)]. Adjustment for the full 10 principal components yielded no further improvement in inflation (data not shown). All principal components were those that were provided with the GERA dataset. Since subjects were genotyped using one of two different kits, we also sought to determine whether the type of kit had any effect on test statistic inflation. In our final analyses (after all exclusions), 1.5% of all subjects were genotyped on the less commonly used kit; when these subjects were excluded, the observed values of λ decreased by 0.02 or less, which was determined to be a negligible amount (data not shown). As a result, no exclusions based on kit type were made in the final analyses.

Interaction test statistic inflation was visually evaluated using quantile-quantile plots. To do this, a random selection of approximately 10,000 SNPs was obtained from each condition-specific dataset, and all possible pairs of SNPs (approximately 5 × 10^7^ pairs) were tested for interaction using the FastEpistasis analytical approach (described below). Observed *vs.* expected-under-the-null −log P-values were plotted, and no evidence of inflation was observed (Figure S1; shown only for the discovery datasets).

### Analytical approaches

All SNPs were diallelic. The analytical referent allele was the major allele (by allele frequency in the discovery dataset), while the nonreferent allele (which may also be known as the “alternative” or “coded” allele) was the minor allele. To assess marginal effects, logistic regression models were used, with SNPs coded additively (based on the number of copies of the nonreferent allele). Unadjusted and adjusted (for birth year category, age, and the first two principal components) analyses were performed for all condition-specific datasets. For ranking of SNPs by marginal effect *P*-value, the adjusted analyses in the discovery datasets were used.

To assess interactions on a genome-wide scale, all pairwise interactions among all postQC SNPs were assessed in each condition-specific discovery dataset using two different approaches: FastEpistasis and BOOST (as implemented in the version of Plink listed below). To avoid spurious evidence of interaction due to linkage disequilibrium between SNPs, an interaction was excluded if its two SNPs were located within 1 Mb of each other. From these genome-wide analyses, interactions with an interaction *P*-value < 10^−7^ were selected for follow-up. For interactions selecting from FastEpistasis analyses, the follow-up consisted of reanalysis of the selected interactions using logistic regression modeling, in both the discovery and replication datasets. Unadjusted and adjusted (for birth year category, age, and the first two principal components) logistic regression analyses were performed. In these models, SNPs were coded additively, and the nonreferent allele was the minor allele in the discovery dataset. Models included main effects for each SNP and an interaction term for the SNPs; statistical significance was based on an explicit test of the interaction term. For interactions selected from BOOST analyses, the follow-up consisted of analysis of the selected interactions in the replication dataset, also using BOOST. For final ranking of interactions by *P*-value, the adjusted analyses of the logistic regression modeling, or the BOOST analyses, from the discovery datasets were used.

### Statistical significance and replication

Nominal significance was defined as *P* < 0.05. Bonferroni correction was used to determine strict statistical significance. For marginal effects, genome-wide significance was defined as *P*-value < 10^−7^ (*i.e.*, 0.05 corrected for tests of the approximately 300,000 SNPs included in each condition-specific marginal analysis). Marginal effect significance was declared based on *P*-values from the analyses that were adjusted for birth year category, sex, and the first two principal components. For interactions, genome-wide significance was defined as interaction *P*-value < 10^−12^ (*i.e.*, 0.05 corrected for tests of the approximately 4.5 × 10^10^ interactions evaluated in each condition-specific analysis). Interaction significance was declared based on *P*-values from the logistic regression (adjusted) or BOOST analyses. For the enrichment analyses, significance was defined as *P*-value < 0.0004 (*i.e.*, 0.05 corrected for 120 enrichment tests).

For marginal effect analyses and interactions assessed with logistic regression (*i.e.*, interactions that were followed-up after the FastEpistasis analyses), an effect was considered replicated if it had both a *P*-value < 0.05 and a consistent direction of effect (with that of the discovery dataset, based on a marginal effect odds ratio or interaction odds ratio, respectively) in the replication dataset. For interactions assessed using BOOST, an interaction was considered replicated only if it had a *P*-value < 0.05 in the replication dataset (directions of effects were not generated in these analyses and thus were not be compared).

### Analytical software

Quality control procedures, marginal effects testing, and genome-wide interaction tests (FastEpistasis and BOOST) were performed using Plink 1.90 β (https://www.cog-genomics.org/plink2) (Chang *et al.* 2015). To assess the accuracy of epistasis tests made using Plink 1.90, we compared its test results to those of similar analyses made using Plink 1.07 for a sample of interactions, and found concordant results (Figure S2). Logistic regression tests for epistasis were performed using CASSI Genome-Wide Interaction Analysis Software v2.50 (https://www.staff.ncl.ac.uk/richard.howey/cassi/).

### Data and reagent availability

File S1 provides a description of all interactions selected for follow-up (https://figshare.com/s/01e151ea20ecb5cdb8db; DOI: 10.6084/m9.figshare.3113551). File S2 provides additional methodological detail. Figure S1 depicts quantile-quantile plots for interactions in the discovery datasets. Figure S2 provides a comparison of epistasis analysis results derived from Plink 1.07 and Plink 1.90. Table S1 contains subject quality control data. Table S2 contains disease-specific subject counts. Table S3 contains SNP quality control data. Table S4 contains genomic inflation data for marginal effects. Table S5 contains database search terms for identifying disease-related genes. Table S6 contains SNP annotation counts. Table S7 contains power analyses for the discovery datasets. Table S8 contains power analyses for the replication datasets. Table S9 contains the penetrance table for epiSIM power simulations. Table S10 contains estimates of phenotypic variance explained by additive genetic variance of the included SNPs. Table S11, Table S12, Table S13, Table S14, Table S15, Table S16, Table S17, Table S18, Table S19, and Table S20 describe the top 10 marginal effects for each studied condition. Table S21, Table S22, Table S23, Table S24, Table S25, Table S26, Table S27, Table S28, Table S29, and Table S30 describe the enrichment analyses for each condition. Table S31 describes the BioGrid interactions.

## Results

### Dataset description

The 10 conditions included as outcomes for analysis, and case/control characteristics for each condition, are shown in Table 1. A majority of the subjects were women, and the median age of the subjects was at or above 59 years of age. All subjects were classified as being of white race/ethnicity. Assuming a logistic regression model, most of the discovery data offered greater than 80% power to detect an interaction with genome-wide significance (assessing all possible pairwise interactions among approximately *N* = 300,000 SNPs, or *N* = 4.5 × 10^10^ interactions), assuming an interactions odds ratio of ≥ 1.50 for SNPs with MAF of ≥ 0.15 (Table S7). The exceptions to this are the data for depression, dermatophytosis, and type 2 diabetes, which only had greater than 50% power. All replication datasets had greater than 80% power to detect the same kind of interaction at a nominal significance of *P* < 0.05 (Table S8). Estimates of the proportion of phenotypic variance explained by additive genetic variance of the included SNPs ranged from 3.6% (dermatophytosis) to 24.6% (diabetes, type 2) (Table S10).

**Table 1:** Subject characteristics

Condition	Dataset	*N*, Cases	*N*, Controls	Min. Age (Years), Range		Min. Age (Years), Median		Male, %	
				Cases	Controls	Cases	Controls	Cases	Controls
Allergic rhinitis	Discovery	10,258	30,933	18–84	18–84	64	59	31.1	37.2
	Replication	976	3004	18–84	18–84	59	59	29.8	35.9
Asthma	Discovery	6486	34,669	18–84	18–84	64	59	28.3	37.1
	Replication	988	3028	18–84	18–84	64	59	26.3	37.4
Cardiac disease	Discovery	11,069	28,979	34–84	34–84	69	59	50.1	30.5
	Replication	1004	3013	34–84	34–84	69	59	46.9	31.2
Depression	Discovery	4824	36,162	24–84	24–84	59	64	24.6	37.3
	Replication	978	2992	24–84	24–84	59	59	24.3	36.9
Dermatophytosis	Discovery	5163	36,083	18–84	18–84	64	59	43.6	34.2
	Replication	989	2936	18–84	18–84	64	59	45.2	35.2
Diabetes, type 2	Discovery	4563	35,573	34–84	34–84	64	59	49.4	33.9
	Replication	986	2943	34–84	34–84	64	59	48.8	33.7
Dyslipidemia	Discovery	23,061	17,021	34–84	34–84	64	59	40.2	29.8
	Replication	986	2997	34–84	34–84	64	59	38.3	30.5
Hemorrhoids	Discovery	6199	34,356	29–84	29–84	64	59	40.7	34.8
	Replication	1006	3117	29–84	29–84	64	59	41.6	34.0
Hypertensive disease	Discovery	21,713	18,332	34–84	34–84	69	54	40.1	31.6
	Replication	984	3036	34–84	34–84	64	59	38.1	30.8
Osteoarthritis	Discovery	15,454	23,578	39–84	39–84	69	59	31.9	38.7
	Replication	961	2985	39–84	39–84	69	59	32.5	38.8

Min. age, the minimum possible age of a subject (see the *Materials and Methods* section).

### Marginal effects

Genome-wide tests of marginal effects (*i.e.*, the effects of SNPs considered individually) were conducted across all 10 conditions (see Table 2 for a summary, and Table S11, Table S12, Table S13, Table S14, Table S15, Table S16, Table S17, Table S18, Table S19, and Table S20 for more extensive analyses). Among the most significant SNPs for each condition, five were genome-wide significant (with *P*-values ranging from 5.75 × 10^−8^ to 1.83 × 10^−37^), three of which were replicated (Table 2). The genome-wide significant SNPs tended to be in or near well-known or highly plausible disease-relevant genes, such as *IL1RL1* (rs2160203) in allergic rhinitis (Li *et al.* 2015), *HLA-DQB1* (rs17612802; 6.9 kb distant) in asthma (Li *et al.* 2010), *TCF7L2* (rs4506565) in type 2 diabetes (Zeggini and McCarthy 2007), *APOB* (rs1367117) in dyslipidemia (Di Taranto *et al.* 2015), and *KCNK3* (rs1275985) in hypertensive disease (Girerd *et al.* 2014). However, no significant marginal effects were found for cardiac disease, depression, dermatophytosis, hemorrhoids, or osteoarthritis.

**Table 2:** Most significant marginal associations, by condition

Condition	RSID	Chr	A1	A0	Discovery, Unadjusted		Discovery, Adjusted		Replication, Adjusted		Genome-wide Sig.?	Rep.?	Anno.	Gene
					OR (95% C.I.)	*P*	OR (95% C.I.)	*P*	OR (95% C.I.)	*P*				
Allergic rhinitis	rs2160203	2	G	A	0.90 (0.87, 0.94)	6.61E-08	0.90 (0.87, 0.94)	5.75E-08	0.93 (0.82, 1.05)	2.16E-01	Yes	No	R, D, G	*IL1RL1*
Asthma	rs17612802	6	C	T	0.87 (0.83, 0.90)	6.01E-13	0.86 (0.83, 0.90)	2.46E-13	0.84 (0.76, 0.94)	1.81E-03	Yes	Yes		(*HLA-DQB1*)*^a^*
Cardiac disease	rs6843082	4	G	A	1.06 (1.02, 1.10)	4.57E-03	1.11 (1.07, 1.16)	7.41E-07	0.97 (0.86, 1.13)	8.51E-01	No	No		
Depression	rs7797095	7	T	C	1.13 (1.07, 1.19)	1.12E-05	1.14 (1.08, 1.20)	4.11E-06	0.94 (0.82, 1.09)	4.18E-01	No	No	G	*KIAA1324L*
Dermatophytosis	rs35626362	4	T	C	0.90 (0.86, 0.94)	2.43E-05	0.89 (0.85, 0.94)	1.12E-05	1.05 (0.93, 1.19)	3.92E-01	No	No		
Diabetes, type 2	rs4506565	10	T	A	1.35 (1.29, 1.41)	1.55E-37	1.35 (1.28, 1.41)	3.22E-36	1.31 (1.17, 1.46)	1.92E-06	Yes	Yes	D, G	*TCF7L2*
Dyslipidemia	rs1367117	2	A	G	1.19 (1.16, 1.23)	4.18E-30	1.24 (1.20, 1.28)	1.83E-37	1.06 (0.94, 1.19)	3.41E-01	Yes	No	EX, D, G	*APOB*
Hemorrhoids	rs6106205	20	C	T	1.11 (1.07, 1.16)	5.52E-07	1.11 (1.07, 1.16)	6.57E-07	1.01 (1.00, 1.00)	9.99E-01	No	No	G	*C20orf26*
Hypertensive dis.	rs1275985	2	C	T	1.09 (1.06, 1.12)	1.02E-08	1.11 (1.07, 1.14)	2.82E-10	1.17 (1.07, 1.34)	1.84E-03	Yes	Yes	G	*KCNK3*
Osteoarthritis	rs6925021	6	A	G	0.94 (0.91, 0.97)	2.96E-04	0.92 (0.89, 0.95)	1.92E-06	1.05 (0.94, 1.19)	3.62E-01	No	No		

Most significant marginal effects, by condition, ranked by significance in each discovery adjusted analysis. RSID, reference SNP cluster ID; A1, nonreferent allele; A0, referent allele; OR, odds ratio; C.I., confidence interval; *P*, *P*-value; Genome-wide sig.?, whether or not the *P*-value from the discovery adjusted analysis was less than 10^−7^; Rep?, whether or not the marginal effect was nominally replicated; Anno., annotation assigned to the respective SNP, coded as follows; R, regulatory; D, disease-gene; G, any-gene; EX, exonic. M (marginal) not shown, since all listed SNPs have that annotation.

a Although not formally assigned a gene annotation due to its distance, this SNP is located 6.9 kb from the *HLA-DQB1* gene.

### Genome-wide search for interactions

The discovery datasets of all 10 conditions were subjected to exhaustive genome-wide searches for pairwise interactions, using both the FastEpistasis (with follow-up via logistic regression) and BOOST analytical approaches. Only one interaction met the criteria for being declared genome-wide significant (interaction *P* < 10^−12^ from the adjusted logistic regression analysis or from the BOOST analysis). This was the interaction between rs4456135 and rs12162346 for the outcome of dermatophytosis (*P* = 4.29 × 10^−13^, logistic regression); however, this was nonsignificant and in opposite direction in the replication dataset (Table 3). For each condition, the most significant interaction, and the most significant nominally replicated interaction, are listed in Table 3 and Table 4 for FastEpistasis and BOOST, respectively. The rank number (by interaction significance) of the most significant nominally replicated interactions ranged from 1 (hemorrhoids; BOOST analysis) to 100 (cardiac disease; FastEpistasis analysis). All followed-up interactions (*i.e.*, those with FastEpistasis or BOOST *P* < 10^−7^ in the discovery datasets) are described in File S1.

**Table 3:** Most significant interactions (overall and among those that were nominally replicated), by condition; FastEpistasis with logistic regression

Condition/Rank	SNP1				SNP2				Discovery				Replication			Rep.?	Anno1	Anno2	Gene1	Gene2
	RSID	Chr	A1	A0	RSID	Chr	A1	A0	FE, *P*	Unadj., *P*	Adj., OR	Adj., *P*	Unadj., *P*	Adj., OR	Adj., *P*					
Allergic rhinitis																				
1	rs493725	11	C	T	rs12456095	18	A	G	1.49E-10	1.52E-10	1.21 (1.14, 1.28)	8.61E-11	6.10E-01	0.94 (0.78, 1.13)	5.29E-01	No	G		*KIRREL3*	
74	rs10710098	12	–	A	rs837473	12	G	A	1.69E-09	2.33E-09	1.22 (1.14, 1.30)	2.08E-09	2.39E-02	1.27 (1.03, 1.57)	2.48E-02	Yes	G		*PPFIBP1*	
Asthma																				
1	rs6764801	3	G	T	rs4574345	4	C	T	2.57E-11	2.84E-11	1.27 (1.19, 1.37)	2.50E-11	1.11E-01	1.20 (0.98, 1.47)	7.62E-02	No				
19	rs7480608	11	A	C	rs996197	20	A	C	8.41E-10	8.31E-10	0.79 (0.73, 0.85)	3.53E-10	5.40E-02	0.81 (0.65, 1.00)	4.68E-02	Yes		G		*C20orf26*
Cardiac disease																				
1	rs1407721	1	T	G	rs455326	5	A	G	5.09E-10	6.86E-10	1.22 (1.15, 1.29)	1.64E-11	4.28E-01	1.01 (0.84, 1.22)	8.97E-01	No				
100	rs9322768	6	C	T	rs6463841	7	G	A	7.47E-09	6.54E-09	0.86 (0.82, 0.91)	4.19E-09	6.08E-02	0.80 (0.69, 0.94)	7.20E-03	Yes		G		*NXPH1*
Depression																				
1	rs16912862	9	G	A	rs4769180	13	C	T	7.78E-11	5.40E-11	0.79 (0.74, 0.85)	1.81E-11	9.92E-01	1.00 (0.85, 1.17)	9.56E-01	No	G		*ZNF169*	
3	rs7587468	2	G	A	rs13120959	4	T	G	1.16E-10	6.72E-11	0.80 (0.75, 0.86)	1.26E-10	5.17E-02	0.84 (0.71, 0.99)	3.51E-02	Yes		G		*PRSS12*
Dermatophytosis																				
1	rs4456135	1	C	T	rs12162346	2	T	C	1.76E-12	2.02E-12	1.29 (1.20, 1.38)	4.29E-13	1.43E-01	0.87 (0.74, 1.04)	1.23E-01	No	G		*LPHN2*	
17	rs4318363	2	C	T	rs7896441	10	G	A	3.02E-10	4.45E-10	1.23 (1.16, 1.32)	1.98E-10	3.54E-02	1.19 (1.01, 1.39)	3.27E-02	Yes		G		*TACC2*
Diabetes, type 2																				
1	rs6677074	1	A	C	rs34332506	3	C	T	4.84E-10	5.33E-10	0.79 (0.73, 0.85)	1.02E-10	8.66E-01	1.00 (0.84, 1.18)	9.81E-01	No	G	G	*CASQ2*	*PCOLCE2*
8	rs4986223	18	C	T	rs59493447	22	T	G	3.29E-10	8.47E-10	1.33 (1.22, 1.45)	3.11E-10	2.83E-02	1.26 (1.01, 1.57)	3.65E-02	Yes	G	D, G	*BC040860*	*SREBF2*
Dyslipidemia																				
1	rs3860935	9	T	C	rs12243792	10	A	G	8.90E-11	8.99E-11	1.20 (1.14, 1.26)	1.09E-11	7.29E-01	1.03 (0.86, 1.24)	7.56E-01	No	G	G, M	*FRMD3*	*ZNF248*
57	rs1655483	11	G	A	rs2617815	19	G	A	4.51E-09	4.18E-09	1.18 (1.12, 1.25)	1.69E-09	3.22E-03	1.28 (1.05, 1.56)	1.33E-02	Yes		R, G		*VAV1*
Hemorrhoids																				
1	rs12043442	1	C	T	rs16858754	3	T	C	4.92E-11	2.00E-11	0.71 (0.65, 0.79)	1.05E-11	3.08E-01	0.88 (0.69, 1.13)	3.21E-01	No				
7	rs2564056	2	C	T	rs13421607	2	T	C	1.14E-10	1.99E-10	1.22 (1.15, 1.30)	2.44E-10	1.31E-02	1.25 (1.06, 1.48)	8.07E-03	Yes				
Hypertensive disease																				
1	rs10187912	2	A	G	rs9929738	16	G	A	9.09E-10	9.27E-10	0.82 (0.77, 0.87)	1.41E-11	1.91E-01	0.92 (0.74, 1.15)	4.63E-01	No				
87	rs7519626	1	C	T	rs17342461	4	C	T	1.52E-08	1.39E-08	0.87 (0.83, 0.91)	2.20E-09	9.65E-02	0.84 (0.71, 0.99)	4.12E-02	Yes				
Osteoarthritis																				
1	rs7316595	12	C	T	rs2066936	19	G	A	1.05E-09	1.05E-09	0.85 (0.81, 0.89)	1.86E-11	9.77E-01	1.06 (0.90, 1.24)	5.03E-01	No	G	G	*LOC643339*	*ZNF773*
6	rs272051	2	G	A	rs7630522	3	T	C	1.08E-08	1.01E-08	1.18 (1.13, 1.25)	1.33E-10	5.87E-02	1.22 (1.01, 1.46)	3.42E-02	Yes				

Interactions were first analyzed with FastEpistasis and then subjected to a follow-up analysis with logistic regression. For each condition, the most significant of all interactions is listed, followed by the most significant interaction that was nominally replicated. Interactions were ranked by significance in the adjusted logistic regression analysis (discovery). Numbers in the leftmost column indicate the overall ranks of the interactions for the respective condition. Blanks in the “Anno” or “Gene” columns indicate no annotation or gene assigned to the respective SNP. SNP, single nucleotide polymorphism; Rep.?, whether or not the interaction was nominally replicated; Anno1/Anno2, annotation assigned to SNP1 or SNP2, respectively; Gene1/Gene2, gene assigned to SNP1 and SNP2, respectively; RSID, reference SNP cluster ID; Chr, chromosome number; A1, nonreferent allele; A0, referent allele. FE, *P*, *P*-value from the FastEpistasis analysis; Unadj., *P*, *P*-value from the unadjusted logistic regression analysis; Adj., OR, interaction odds ratio and 95% confidence interval, from the adjusted logistic regression analysis; Adj., *P*, *P*-value from the adjusted logistic regression analysis; All “adjusted” analyses were adjusted for the first two principal components, birth year category, and sex; G, any-gene; D, disease-gene; M, marginal; R, regulatory.

**Table 4:** Most significant interactions (overall and among those that were nominally replicated), by condition; BOOST

Condition/Rank	SNP1				SNP2				Discovery *P*	Replication *P*	Rep.?	Anno1	Anno2	Gene1	Gene2
	RSID	Chr	A1	A0	RSID	Chr	A1	A0							
Allergic rhinitis															
1	rs13403689	2	T	G	rs11086806	20	G	T	3.92E-11	1.52E-01	No	G	G	*OSBPL6*	*CHD6*
31	rs7581504	2	A	G	rs11086806	20	G	T	7.50E-10	4.97E-02	Yes	G, M	G	*OSBPL6*	*CHD6*
Asthma															
1	rs1461773	6	A	G	rs1362930	7	T	C	2.55E-11	6.31E-01	No	G		*OPRM1*	
30	rs6722509	2	C	T	rs11709714	3	A	G	8.78E-10	3.73E-02	Yes	G	G	*OR6B3*	*CP*
Cardiac disease															
1	rs12943579	17	G	A	rs886617	22	T	C	6.54E-11	7.48E-01	No		G		*SEC14L6*
26	rs2580405	2	T	G	rs2355635	10	C	T	4.51E-10	3.72E-02	Yes				
Depression															
1	rs2651975	12	C	A	rs9940287	16	T	C	4.85E-11	6.67E-02	No	G		*TMCC3*	
18	rs6414384	3	G	A	rs10843021	12	T	C	6.16E-10	2.27E-02	Yes	G		*KCNAB1*	
Dermatophytosis															
1	rs74378451	10	G	A	rs1536032	13	A	G	1.20E-12	9.44E-01	No	G	M	*RNLS*	
18	rs400883	4	G	T	rs12897227	14	A	G	3.22E-10	2.61E-03	Yes	G	G	*ANK2*	*KTN1*
Diabetes, type 2															
1	rs1327614	1	G	A	rs12895385	14	C	T	2.33E-11	6.92E-01	No				
2	rs11900922	2	C	T	rs770116	12	T	C	2.69E-11	7.99E-03	Yes		D, G		*NAV3*
Dyslipidemia															
1	rs7646670	3	T	C	rs72841214	17	T	C	2.80E-11	6.73E-01	No				
10	rs251162	5	G	A	rs3783322	14	G	A	2.84E-10	4.95E-02	Yes		G		*EML1*
Hemorrhoids															
1	rs11684491	2	G	A	rs6792001	3	A	G	1.18E-11	2.97E-02	Yes				
Hypertensive disease															
1	rs3128854	6	A	G	rs75377761	6	C	T	1.09E-11	5.66E-01	No	G	G	*OR2H1*	*COQ3*
15	rs2310357	4	T	C	rs4449525	5	G	A	2.51E-10	1.56E-02	Yes	R, G		*SORBS2*	
Osteoarthritis															
1	rs57799846	1	A	G	rs8046139	16	C	T	1.85E-11	5.33E-01	No	G		*KCNK1*	
14	rs4408841	3	A	G	rs4858960	3	A	C	2.78E-10	1.69E-02	Yes				

For each condition, the most significant of all interactions is listed, followed by the most significant interaction that was nominally replicated. Interactions were ranked by significance in the BOOST analysis (discovery). Numbers in the leftmost column indicate the overall ranks of the interactions for the respective condition. Blanks in the “Anno” or “Gene” columns indicate no annotation or gene assigned to the respective SNP. SNP, single nucleotide polymorphism; Discovery *P*, *P*-value from the discovery BOOST analysis; Replication *P*, *P*-value from the replication BOOST analysis; Rep.?, whether or not the interaction was nominally replicated; Anno1/Anno2, annotation assigned to SNP1 or SNP2, respectively; Gene1/Gene2, gene assigned to SNP1 and SNP2, respectively; RSID, reference SNP cluster ID; Chr, chromosome number; A1, nonreferent allele; A0, referent allele; G, any-gene; M, marginal; D, disease-gene; R, regulatory.

### Enrichment analysis

We next attempted to determine if there was an enrichment of nominally replicated interactions with particular SNP annotations (based on six possible categories; see the Supplemental Materials), among all followed-up interactions. These results are shown in Table S21, Table S22, Table S23, Table S24, Table S25, Table S26, Table S27, Table S28, Table S29, and Table S30. The only annotation category that showed evidence for enrichment was that of “marginal” (SNPs with a marginal effect *P* < 0.05 in the discovery dataset of the respective condition). Both cardiac disease and hypertensive disease showed strictly significant (*P* < 0.0004) evidence for this type of enrichment, while dyslipidemia also showed nominally significant evidence (*P* < 0.05) for the same. For example, in cardiac disease (analyzed by logistic regression; Table S23), among interactions where both SNPs had the “marginal” annotation, 20.0% were nominally replicated; while among interactions where neither of the SNPs had this annotation, only 2.5% were nominally replicated.

However, no annotation strategy could have been used to successfully narrow the search space in order to find a replicated, significant interaction. This can be seen in Table S21, Table S22, Table S23, Table S24, Table S25, Table S26, Table S27, Table S28, Table S29, and Table S30, comparing entries in the columns “Min Int P” with prefix “R” (the smallest *P*-value among all replicated interactions matching the stated annotation type) and “Thresh” (a hypothetical significance threshold, defined as 0.05 corrected for the number of interactions that would have resulted had the search been limited to interactions matching the stated annotation type). For example, in cardiac disease, there were *N* = 16,284 SNPs with the “marginal” annotation (Table S6). An exhaustive pairwise analysis of just these SNPs would result in 1.3 × 10^8^ evaluated interactions and a significance threshold of *P* < 3.77 × 10^−10^. The most significant replicated interaction among all of these had an actual *P*-value of 1.09 × 10^−7^ (Table S23), and thus would not have been considered significant. Therefore, although we can detect a possible enrichment among interactions with marginal effects, it may not be possible to use this filtering approach alone to identify which of the interactions are actually be genuine; effect sizes may be too small to detect.

### BioGRID interactions

Finally, we sought to determine whether any interaction that met the criteria for follow-up involved genes (or their products) that have been previously reported to physically or genetically interact, as curated in the BioGRID database. Across all analyzed conditions, there were three BioGRID interactions among the followed-up interactions for the FastEpistasis analyses, and two for the BOOST analyses (Table S31). However, none of these interactions were nominally replicated.

## Discussion

To our knowledge, this study presents one of the largest (by subject count) searches for SNP-SNP interactions yet conducted for human disease. Despite this, and the use of two different analytical methods, we failed to detect a significant, replicable interaction after exhaustively searching through 45 billion possible interactions in each of 10 different complex diseases. One possible explanation for this is that no interactions exist for these conditions involving SNPs with a MAF ≥ 0.15 operating under the interaction models specified in the power analyses. If SNP-SNP interactions do contribute to the conditions that were analyzed, then they are likely of weak effect and/or involve lower-frequency SNPs, which will be very challenging to study. Statistical power drops off rapidly as the interaction odds ratio goes from 1.50–1.25; even at a modest significance level of *P* < 10^−7^, we did not have power to detect an interaction with an odds ratio of 1.25 for any condition (Table S7).

In addition, SNP annotation by functionality (exonic function, regulatory function, and disease-relevant eQTL) and gene assignment (disease-relevant gene and any gene) failed to result in any appreciable enrichment in replicated interactions, suggesting that these methods alone may not be useful in trimming the epistasis search space. Only SNPs with marginal significance showed evidence for enrichment, for some outcomes. Interestingly, this is consistent with a recent suggestion that “epistatic effects that seem to be statistically robust have large marginal effects” (Wei *et al.* 2014). Furthermore, among all interactions that met follow-up criteria, few involved genes were reported to interact in the BioGRID database, and none of these few were nominally replicated. This suggests that robust statistical SNP-SNP interactions do not correspond to gene pairs whose products are known to interact physically (since the majority of interactions listed in the BioGRID data were identified based on protein-protein interactions).

Apart from the nonexistence of strong (large effect size) interactions, there are a number of possible alternative explanations for our observed lack of detection. The first is that some of the included conditions may have been too broadly defined to capture a strong association signal, since they were defined based on having two or more of several possible ICD-9 codes within a diagnostic class, and it was not possible to ascertain narrower phenotypes. This might particularly have been a problem for the outcome of “cardiac disease,” which was constructed out of a large of number of disparate codes (http://www.ncbi.nlm.nih.gov/projects/gap/cgi-bin/GetPdf.cgi?id=phd004308). Moreover, it may be possible that associations will differ depending on age of onset, which we did not have information on. The etiology of some of the conditions (*e.g.*, dermatophytosis and hemorrhoids, which had few entries in the Genetic Association Database and the DisGenNET database; Table S5) may simply have a weaker genetic basis than others, which could also explain a lack of association. For half of the conditions (allergic rhinitis, asthma, type 2 diabetes, dyslipidemia, and hypertensive disease), strong marginal effects involving well-known disease-relevant genes were found, which supports the validity of the analytical approach for these conditions. However, no strictly significant marginal effects were found for the other half (cardiac disease, depression, dermatophytosis, hemorrhoids, and osteoarthritis), which could be attributable to the aforementioned problems. This is also suggested by the observation that the additive genetic variance captured by the included SNPs was low for some conditions (Table S10**)**. In addition, the datasets for depression, dermatophytosis, and type 2 diabetes had lower power in general than the other conditions (Table S7), which could also explain a lack of interactions for these conditions in particular. Furthermore, we excluded interactions involving SNPs located within 1 Mb of one another, to prevent the analysis of SNPs in linkage disequilibrium; however, this may have also excluded genuine interactions involving close-proximity SNPs. Additionally, power was estimated assuming a limited number of interaction models and no noise in the data; as such, it is likely that the analytical approaches (FastEpistasis, BOOST, and logistic regression) had low power to detect interaction models that are not captured well by these methods. Finally, epistasis may involve higher orders of interaction that may not be apparent in the second-order (pairwise) interactions that were examined in this study.

Limitations of the enrichment analysis include the fact that the exonic and regulatory annotations only considered directly typed SNPs, and that the eQTL and disease gene annotations assumed that typed SNPs were within a short distance of an eQTL (± 1 kb) or a disease gene (± 5 kb). Although these choices were made in order to limit the number of annotated SNPs, they could potentially miss SNPs that were in linkage disequilibrium with these features but located a farther distance away. In addition, the disease gene annotations relied on literature curation databases that may have different levels of completeness or validity for different conditions. It is also possible that other annotation methods not considered here (such as the use of Biofilter (Bush *et al.* 2009)) may have greater success than we found.

Given the development of statistical methodologies that enable the computationally efficient evaluation of vast numbers of interactions, and the increasing availability of very large genetic association databases, it is becoming increasingly feasible to search for SNP-SNP interactions on a genome-wide scale. However, based on the experiences found in the present study, these interactions are likely to have small effect sizes, involve low-frequency variants, and/or involve complex models of interaction, which may require alternative methods of detection.

## Supplementary Material

Supplemental Material
