# Supplementary material for: Exhaustive Genome-Wide Search for SNP-SNP Interactions Across 10 Human Diseases
Source: G3 (Bethesda). 2016 May 12;6(7):2043–50. doi: 10.1534/g3.116.028563 (PMC4938657; doi:10.1534/g3.116.028563)
Supplement: Supplemental Material [file supp_6_7_2043__index.html]

Exhaustive Genome-Wide Search for SNP-SNP Interactions Across 10 Human Diseases — Supplemental Material 

# Exhaustive Genome-Wide Search for SNP-SNP Interactions Across 10 Human Diseases

## Supplemental Material for Murk and DeWan, 2016

**Files in this Data Supplement:**

- Figure S1 - Quantile-quantile plots for interaction P-values from the discovery datasets. (.pdf, 425 KB)
- Table S7 - Power analyses, discovery datasets. (.pdf, 337 KB)
- Table S8 - Power analyses, replication datasets. (.pdf, 283 KB)
- Table S9 - Penetrance table for the model simulated in epiSIM. (.pdf, 269 KB)
- Table S10 - Estimation of phenotypic variance explained by additive genetic variance of the included SNPs. (.pdf, 281 KB)
- Table S11 - Top 10 most significant marginal associations, allergic rhinitis. (.pdf 387 KB)
- Table S12 - All genome-wide significant marginal associations, asthma. (.pdf, 469 KB)
- Table S13 - Top 10 most significant marginal associations, cardiac disease. (.pdf, 385 KB)
- Table S14 - Top 10 most significant marginal associations, depression. (.pdf, 385 KB)
- Table S15 - Top 10 most significant marginal associations, dermatophytosis. (.pdf, 385 KB)
- Table S16 - All genome-wide significant marginal associations, type 2 diabetes. (.pdf, 398 KB)
- Figure S2 - Comparison of epistasis analysis results derived from Plink 1.07 and Plink 1.90. (.pdf, 340 KB)
- Table S17 - All genome-wide significant marginal associations, dyslipidaemia. (.pdf, 551 KB)
- Table S18 - Top 10 most significant marginal associations, hemorrhoids. (.pdf, 385 KB)
- Table S19 - Top 10 most significant marginal associations, hypertensive disease. (.pdf, 386 KB)
- Table S20 - Top 10 most significant marginal associations, osteoarthritis. (.pdf, 385 KB)
- Table S21 - Enrichment analysis (allergic rhinitis). (.pdf, 287 KB)
- Table S22 - Enrichment analysis (asthma). (.pdf, 286 KB)
- Table S23 - Enrichment analysis (cardiac disease). (.pdf, 287 KB)
- Table S24 - Enrichment analysis (depression). (.pdf, 285 KB)
- Table S25 - Enrichment analysis (dermatophytosis). (.pdf, 286 KB)
- Table S26 - Enrichment analysis (diabetes, type 2). (.pdf, 286 KB)
- Table S27 - Enrichment analysis (dyslipidaemia). (.pdf, 286 KB)
- Table S28 - Enrichment analysis (hemorrhoids). (.pdf, 286 KB)
- Table S29 - Enrichment analysis (hypertensive disease). (.pdf, 286 KB)
- Table S30 - Enrichment analysis (osteoarthritis). (.pdf, 286 KB)
- Table S31 - BioGRID interactions (FastEpistasis with logistic regression), by condition. (.pdf, 286 KB)
- File S2 - Supplemental methods. (.pdf, 385 KB)
- Table S1 - Subject quality control. (.pdf, 273 KB)
- Table S2 - Disease-specific subject counts. (.pdf, 280 KB)
- Table S3 - SNP quality control. (.pdf, 281 KB)
- Table S4 - Genomic inflation, marginal effect. (.pdf, 282 KB)
- Table S5 - Database search for previously known disease-related candidate genes. (.pdf, 276 KB)
- Table S6 - SNP annotations, by category. (.pdf, 283 KB)
- File S1 - Descriptions of all interactions selected for follow-up. (.xlsx, 25 MB)
