# Supplementary material for: Exhaustive Genome-Wide Search for SNP-SNP Interactions Across 10 Human Diseases
Source: G3 (Bethesda). 2016 May 12;6(7):2043–50. doi: 10.1534/g3.116.028563 (PMC4938657; doi:10.1534/g3.116.028563)
Supplement: Supplemental Material [file supp_g3.116.028563_FigureS1.pdf]

Figure S-1. Quantile-quantile plots for interaction P-values from the discovery datasets.

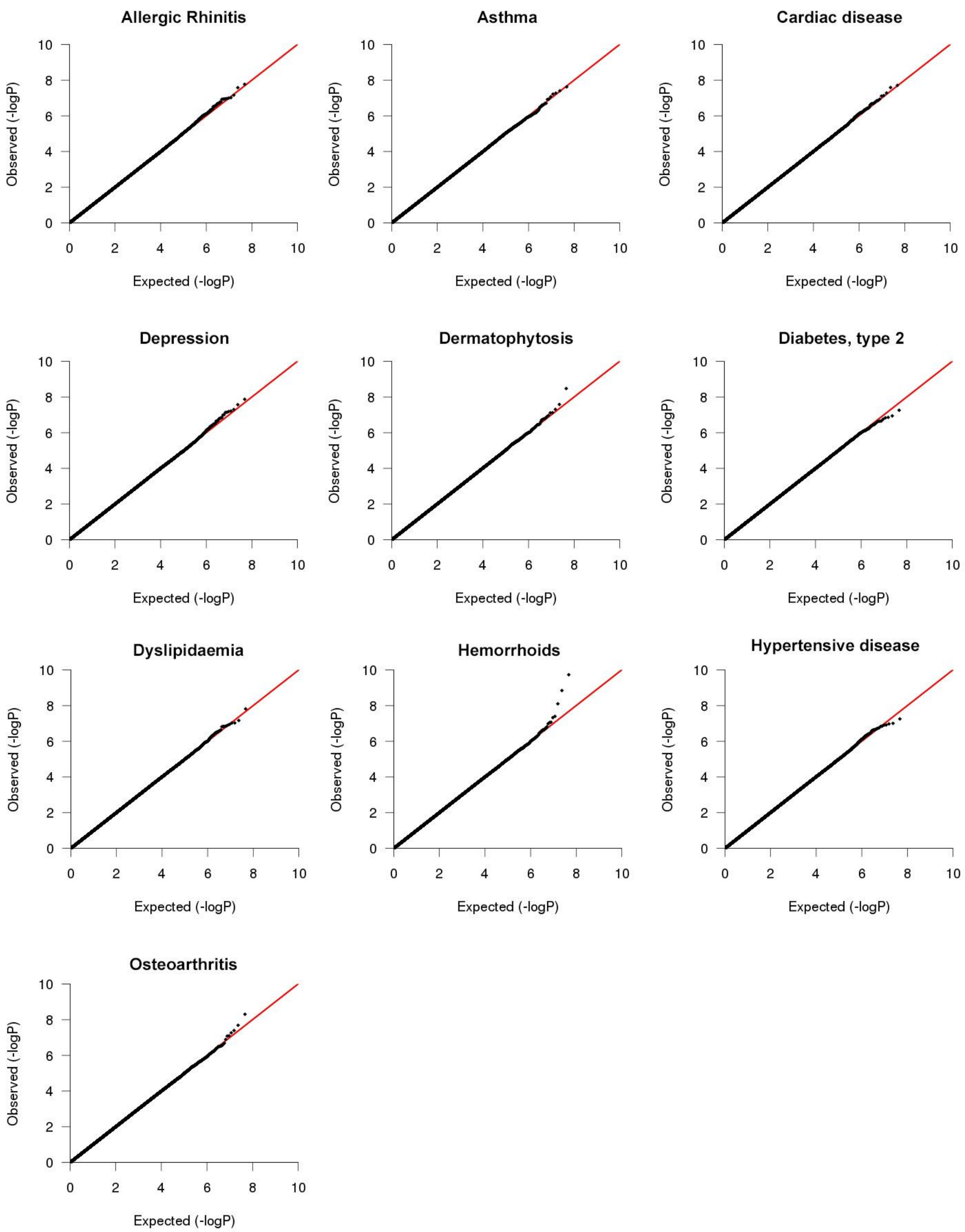

Interaction P-values were obtained via FastEpistasis, among all pairwise interactions involving approximately 10,000 randomly selected SNPs in each discovery dataset (approximately 5×10^7 pairs each).
