# Supplementary material for: Exhaustive Genome-Wide Search for SNP-SNP Interactions Across 10 Human Diseases
Source: G3 (Bethesda). 2016 May 12;6(7):2043–50. doi: 10.1534/g3.116.028563 (PMC4938657; doi:10.1534/g3.116.028563)
Supplement: Supplemental Material [file supp_g3.116.028563_TableS7.pdf]

Table S-7. Power analyses, discovery datasets.

Condition	Type I error (α)	Quanto		Simulated					
				Fast epistasis			BOOST		
		Int OR = 1.25	Int OR = 1.50	f = 0.15	f = 0.20	f = 0.25	f = 0.15	f = 0.20	f = 0.25
**Allergic rhinitis**	E-7	0.439	0.999	1.00	1.00	1.00	1.00	1.00	1.00
	E-8	0.289	0.999	1.00	1.00	1.00	1.00	1.00	1.00
Cases = 10,258	E-9	0.175	0.999	0.93	1.00	1.00	1.00	1.00	1.00
Controls = 30,933	E-10	0.099	0.999	0.91	1.00	1.00	1.00	1.00	1.00
Prev = 0.25	E-11	0.051	0.997	0.87	1.00	1.00	1.00	1.00	1.00
b = 0.243, 0.241, 0.238	E-12	0.025	0.992	0.73	1.00	1.00	1.00	1.00	1.00
**Asthma**	E-7	0.187	0.999	0.63	0.99	1.00	0.41	0.93	1.00
	E-8	0.098	0.996	0.48	0.95	1.00	0.25	0.91	1.00
Cases = 6,486	E-9	0.047	0.987	0.27	0.92	1.00	0.16	0.79	0.99
Controls = 34,669	E-10	0.021	0.970	0.19	0.86	1.00	0.08	0.68	0.99
Prev = 0.15	E-11	0.009	0.938	0.12	0.73	0.99	0.03	0.57	0.99
b = 0.146, 0.144, 0.143	E-12	0.004	0.888	0.08	0.64	0.99	0.01	0.51	0.99
**Cardiac disease**	E-7	0.475	0.999	1.00	1.00	1.00	1.00	1.00	1.00
	E-8	0.321	0.999	1.00	1.00	1.00	0.99	1.00	1.00
Cases = 11,069	E-9	0.199	0.999	0.99	1.00	1.00	0.99	1.00	1.00
Controls = 28,979	E-10	0.115	0.999	0.99	1.00	1.00	0.95	1.00	1.00
Prev = 0.25	E-11	0.062	0.998	0.97	1.00	1.00	0.90	1.00	1.00
b = 0.243, 0.241, 0.239	E-12	0.031	0.995	0.92	1.00	1.00	0.84	1.00	1.00
**Depression**	E-7	0.081	0.981	0.29	0.81	1.00	0.18	0.57	0.95
	E-8	0.036	0.953	0.21	0.69	0.99	0.07	0.49	0.92
Cases = 4,824	E-9	0.015	0.903	0.14	0.54	0.93	0.01	0.38	0.87
Controls = 36,162	E-10	0.006	0.827	0.04	0.41	0.89	0.01	0.29	0.82
Prev = 0.15	E-11	0.002	0.727	0.01	0.33	0.84	0.01	0.21	0.73
b = 0.146, 0.145, 0.143	E-12	0.001	0.610	0.01	0.27	0.80	0.00	0.11	0.63
**Dermatophytosis**	E-7	0.100	0.989	0.45	0.96	1.00	0.24	0.85	1.00
	E-8	0.046	0.971	0.28	0.89	1.00	0.11	0.75	1.00
Cases = 5,163	E-9	0.020	0.935	0.15	0.81	1.00	0.04	0.57	0.99
Controls = 36,083	E-10	0.008	0.876	0.08	0.71	1.00	0.02	0.45	0.94
Prev = 0.15	E-11	0.003	0.793	0.04	0.56	0.98	0.00	0.27	0.90
b = 0.146, 0.145, 0.143	E-12	0.001	0.689	0.01	0.42	0.95	0.00	0.20	0.85
**Diabetes, type 2**	E-7	0.067	0.973	0.21	0.74	0.99	0.10	0.55	0.95
	E-8	0.029	0.933	0.14	0.59	0.97	0.05	0.44	0.87
Cases = 4,563	E-9	0.011	0.868	0.08	0.50	0.92	0.02	0.28	0.81
Controls = 35,573	E-10	0.004	0.777	0.02	0.34	0.84	0.01	0.18	0.72
Prev = 0.15	E-11	0.001	0.663	0.01	0.25	0.79	0.01	0.12	0.60
b = 0.146, 0.145, 0.143	E-12	0.001	0.539	0.01	0.19	0.74	0.00	0.06	0.51
**Dyslipidaemia**	E-7	0.582	0.999	1.00	N/A	N/A	1.00	N/A	N/A
	E-8	0.422	0.999	1.00	N/A	N/A	1.00	N/A	N/A
Cases = 23,061	E-9	0.282	0.999	1.00	N/A	N/A	1.00	N/A	N/A
Controls = 17,021	E-10	0.175	0.999	1.00	N/A	N/A	1.00	N/A	N/A
Prev = 0.55	E-11	0.101	0.999	1.00	N/A	N/A	1.00	N/A	N/A
b = 0.535	E-12	0.055	0.997	1.00	N/A	N/A	1.00	N/A	N/A
**Hemorrhoids**	E-7	0.165	0.998	0.63	0.97	1.00	0.32	0.92	1.00
	E-8	0.084	0.993	0.41	0.94	1.00	0.17	0.88	1.00
Cases = 6,199	E-9	0.039	0.981	0.23	0.89	1.00	0.08	0.80	1.00
Controls = 34,356	E-10	0.017	0.957	0.15	0.85	1.00	0.06	0.66	1.00
Prev = 0.15	E-11	0.007	0.916	0.07	0.76	1.00	0.03	0.49	0.98
b= 0.146, 0.145, 0.143	E-12	0.003	0.854	0.04	0.62	0.99	0.00	0.38	0.94
**Hypertensive disease**	E-7	0.613	0.999	1.00	N/A	N/A	1.00	N/A	N/A
	E-8	0.453	0.999	1.00	N/A	N/A	1.00	N/A	N/A
Cases = 21,713	E-9	0.310	0.999	1.00	N/A	N/A	1.00	N/A	N/A
Controls = 18,332	E-10	0.196	0.999	1.00	N/A	N/A	1.00	N/A	N/A
Prev = 0.50	E-11	0.116	0.999	1.00	N/A	N/A	1.00	N/A	N/A
b = 0.485	E-12	0.065	0.998	1.00	N/A	N/A	1.00	N/A	N/A
**Osteoarthritis**	E-7	0.587	0.999	1.00	1.00	1.00	1.00	1.00	1.00
	E-8	0.427	0.999	1.00	1.00	1.00	1.00	1.00	1.00
Cases = 15,454	E-9	0.287	0.999	1.00	1.00	1.00	1.00	1.00	1.00
Controls = 23,578	E-10	0.179	0.999	1.00	1.00	1.00	1.00	1.00	1.00
Prev = 0.40	E-11	0.104	0.999	1.00	1.00	1.00	1.00	1.00	1.00
b = 0.389, 0.385, 0.381	E-12	0.057	0.998	1.00	1.00	1.00	1.00	1.00	1.00

f: relative penetrance. b: baseline penentrance, corresponding to each respective relative penetrance going from left to right. Green indicates power > 80%. Yellow indicates the genome-wide significance threshold. N/A: not estimated because a model penetrance would exceed 1.0.
