# Supplementary material for: Exhaustive Genome-Wide Search for SNP-SNP Interactions Across 10 Human Diseases
Source: G3 (Bethesda). 2016 May 12;6(7):2043–50. doi: 10.1534/g3.116.028563 (PMC4938657; doi:10.1534/g3.116.028563)
Supplement: Supplemental Material [file supp_g3.116.028563_TableS8.pdf]

**Table S-8. Power analyses, replication datasets.**

| Condition | Type I error (α) | Quanto | | Simulated | | | | | |
|---|---|---|---|---|---|---|---|---|---|
| | | | | Fast epistasis | | | BOOST | | |
| | | OR = 1.25 | OR = 1.50 | f = 0.15 | f = 0.20 | f = 0.25 | f = 0.15 | f = 0.20 | f = 0.25 |
| **Allergic rhinitis**<br>Cases = 976<br>Controls = 3,004<br>Prev = 0.25<br>b = 0.243, 0.241, 0.238 | 0.05 | 0.363 | 0.845 | 0.66 | 0.90 | 0.99 | 0.46 | 0.81 | 0.94 |
| **Asthma**<br>Cases = 988<br>Controls = 3,028<br>Prev = 0.15<br>b = 0.146, 0.144, 0.143 | 0.05 | 0.366 | 0.856 | 0.49 | 0.77 | 0.92 | 0.28 | 0.57 | 0.80 |
| **Cardiac disease**<br>Cases = 1,004<br>Controls = 3,013<br>Prev = 0.25<br>b = 0.243, 0.241, 0.239 | 0.05 | 0.366 | 0.848 | 0.75 | 0.96 | 0.99 | 0.54 | 0.85 | 0.98 |
| **Depression**<br>Cases = 978<br>Controls = 2,992<br>Prev = 0.15<br>b = 0.146, 0.145, 0.143 | 0.05 | 0.367 | 0.857 | 0.48 | 0.69 | 0.90 | 0.33 | 0.54 | 0.74 |
| **Dermatophytosis**<br>Cases = 989<br>Controls = 2,936<br>Prev = 0.15<br>b = 0.146, 0.145, 0.143 | 0.05 | 0.368 | 0.857 | 0.53 | 0.77 | 0.91 | 0.29 | 0.56 | 0.79 |
| **Diabetes, type 2**<br>Cases = 986<br>Controls = 2,943<br>Prev = 0.15<br>b = 0.146, 0.145, 0.143 | 0.05 | 0.367 | 0.857 | 0.42 | 0.66 | 0.90 | 0.29 | 0.44 | 0.76 |
| **Dyslipidaemia**<br>Cases = 986<br>Controls = 2,997<br>Prev = 0.55<br>b = 0.535 | 0.05 | 0.343 | 0.802 | 1.00 | N/A | N/A | 1.00 | N/A | N/A |
| **Hemorrhoids**<br>Cases = 1,006<br>Controls = 3,117<br>Prev = 0.15<br>b= 0.146, 0.145, 0.143 | 0.05 | 0.351 | 0.812 | 0.63 | 0.80 | 0.93 | 0.44 | 0.72 | 0.86 |
| **Hypertensive disease**<br>Cases = 984<br>Controls = 3,036<br>Prev = 0.50<br>b = 0.485 | 0.05 | 0.347 | 0.809 | 1.00 | N/A | N/A | 0.98 | N/A | N/A |
| **Osteoarthritis**<br>Cases = 961<br>Controls = 2,985<br>Prev = 0.40<br>b = 0.389, 0.385, 0.381 | 0.05 | 0.346 | 0.814 | 0.93 | 1.00 | 1.00 | 0.82 | 0.98 | 1.00 |

f: relative penetrance. b: baseline penetrance, corresponding to each respective relative penetrance going from left to right. Green indicates power > 80%. N/A: not estimated because a model penetrance would exceed 1.0.
