# Supplementary material for: Exhaustive Genome-Wide Search for SNP-SNP Interactions Across 10 Human Diseases
Source: G3 (Bethesda). 2016 May 12;6(7):2043–50. doi: 10.1534/g3.116.028563 (PMC4938657; doi:10.1534/g3.116.028563)
Supplement: Supplemental Material [file supp_g3.116.028563_TableS9.pdf]

Table S-9. Penetrance table for the model simulated in epiSIM.

		SNP A genotype		
		AA	Aa	aa
**SNP B genotype**	BB	b	b	b
	Bb	b	$b(1 + f)^2$	$b(1 + f)^3$
	bb	b	$b(1 + f)^3$	$b(1 + f)^4$

b: baseline penentrance. f: relative penetrance.
