# Supplementary material for: Exhaustive Genome-Wide Search for SNP-SNP Interactions Across 10 Human Diseases
Source: G3 (Bethesda). 2016 May 12;6(7):2043–50. doi: 10.1534/g3.116.028563 (PMC4938657; doi:10.1534/g3.116.028563)
Supplement: Supplemental Material [file supp_g3.116.028563_TableS10.pdf]

Table S-10. Estimation of phenotypic variance explained by additive genetic variance of the included SNPs.

Condition	Prevalence	V(G)		Vp		V(G)/Vp		V(G)/Vp_L		P-val	n
		Estimate	SE	Estimate	SE	Estimate	SE	Estimate	SE		
Allergic rhinitis	0.25	0.013	0.009	0.250	0.004	0.054	0.036	**0.075**	0.050	6.2E-02	8058
Asthma	0.15	0.031	0.009	0.250	0.004	0.125	0.036	**0.149**	0.043	3.3E-05	8012
Cardiac disease	0.25	0.021	0.009	0.250	0.004	0.085	0.037	**0.119**	0.051	6.4E-03	7916
Depression	0.15	0.009	0.009	0.250	0.004	0.038	0.036	**0.045**	0.042	1.4E-01	7953
Dermatophytosis	0.15	0.007	0.009	0.250	0.004	0.030	0.034	**0.036**	0.041	1.8E-01	8079
Diabetes, type 2	0.15	0.051	0.010	0.250	0.004	0.206	0.039	**0.246**	0.046	6.2E-09	7987
Dyslipidaemia	0.55	0.017	0.009	0.250	0.004	0.069	0.037	**0.107**	0.057	2.7E-02	7915
Hemorrhoids	0.15	0.012	0.009	0.250	0.004	0.047	0.036	**0.057**	0.043	8.6E-02	7998
Hypertensive disease	0.50	0.018	0.009	0.250	0.004	0.071	0.035	**0.111**	0.055	1.4E-02	8109
Osteoarthritis	0.40	0.006	0.009	0.250	0.004	0.024	0.035	**0.037**	0.054	2.5E-01	7998

V(G): Genetic variance. **Vp:** Phenotypic variance. **V(G)/Vp:** Genetic variance over the phenotypic variance (variance explained). **V(G)/Vp_L** (emphasized in bold): Variance explained when transformed from the observed scale to the underlying scale (based on the prevalence estimate). **P-val**: P-value for a likelihood ratio test comparing full and reduced models with and without the genetic variance component. Residual variance estimates are not shown. **n**: Total number of subjects included for each analysis. Approximately 300,000 SNPs were included in each analysis (see **Table S-3** for precise numbers).
