# Supplementary material for: Exhaustive Genome-Wide Search for SNP-SNP Interactions Across 10 Human Diseases
Source: G3 (Bethesda). 2016 May 12;6(7):2043–50. doi: 10.1534/g3.116.028563 (PMC4938657; doi:10.1534/g3.116.028563)
Supplement: Supplemental Material [file supp_g3.116.028563_TableS11.pdf]

Explanation for Tables S-11 through S-20:

These tables present either the top 10 most significant marginal associations, or all genome-wide significant marginal associations, for the ten outcome conditions. SNPs were ranked by significance in the adjusted discovery analysis. **A1**: non-referent allele. **A0**: referent allele. **OR:** odds ratio; **CI:** confidence interval. **P**: P-value**. Genome-wide sig.?** Whether or not the P-value from the discovery adjusted analysis was less than 10^{-7}. **Rep?** Whether or not the marginal effect was nominally replicated. **Annotation**: Annotation assigned to the respective SNP, coded as follows: EX, exonic; R, regulatory; D, disease-gene; Q, disease-eQTL; G, any-gene. Marginal annotations (M) are not listed, since all SNPs in these tables would have that annotation. Green shading indicates genome-wide significant or replicated.

Table S-11. Top 10 most significant marginal associations, allergic rhinitis.

RSID	Chr	Position	A1	A0	Discovery, unadjusted		Discovery, adjusted		Replication, adjusted		Genome-wide sig.?	Replicated?	Annotation	Gene
					OR (95% CI)	P	OR (95% CI)	P	OR (95% CI)	P				
rs2160203	2	102960824	G	A	0.90 (0.87, 0.94)	6.61E-08	0.90 (0.87, 0.94)	5.75E-08	0.93 (0.82, 1.05)	2.16E-01	Yes	No	R, D, G,	IL1RL1
rs34624588	5	110452316	-	T	0.92 (0.89, 0.95)	1.25E-07	0.92 (0.89, 0.95)	1.11E-07	0.95 (0.85, 1.05)	2.95E-01	No	No	G,	WDR36
rs3806933	5	110406742	T	C	0.92 (0.89, 0.95)	1.50E-07	0.92 (0.89, 0.95)	1.17E-07	0.96 (0.87, 1.07)	4.66E-01	No	No	D, G,	TSLP
rs4988956	2	102968007	A	G	0.91 (0.89, 0.95)	9.61E-08	0.92 (0.89, 0.95)	1.21E-07	0.94 (0.84, 1.04)	2.10E-01	No	No	EX, R, D, G,	IL1RL1
rs10173193	2	102975050	A	G	0.92 (0.89, 0.95)	1.81E-07	0.92 (0.89, 0.95)	2.15E-07	0.94 (0.84, 1.04)	1.93E-01	No	No	G,	IL18R1
rs10206753	2	102968362	C	T	0.92 (0.89, 0.95)	1.73E-07	0.92 (0.89, 0.95)	2.25E-07	0.94 (0.84, 1.04)	2.36E-01	No	No	EX, D, G,	IL1RL1
rs10192036	2	102968211	A	C	0.92 (0.89, 0.95)	2.05E-07	0.92 (0.89, 0.95)	2.65E-07	0.94 (0.84, 1.05)	2.53E-01	No	No	EX, D, G,	IL1RL1
rs1438673	5	110467499	C	T	1.09 (1.05, 1.12)	4.91E-07	1.09 (1.05, 1.12)	3.75E-07	1.04 (0.93, 1.15)	5.23E-01	No	No	G,	WDR36
rs1592459	2	103031569	G	A	0.92 (0.89, 0.95)	5.41E-07	0.92 (0.89, 0.95)	7.61E-07	0.98 (0.88, 1.09)	6.92E-01	No	No	G,	IL18RAP
rs6710528	2	103016142	T	C	0.92 (0.89, 0.95)	6.85E-07	0.92 (0.89, 0.95)	9.56E-07	0.98 (0.88, 1.08)	5.80E-01	No	No	G,	IL18R1
