# Supplementary material for: Exhaustive Genome-Wide Search for SNP-SNP Interactions Across 10 Human Diseases
Source: G3 (Bethesda). 2016 May 12;6(7):2043–50. doi: 10.1534/g3.116.028563 (PMC4938657; doi:10.1534/g3.116.028563)
Supplement: Supplemental Material [file supp_g3.116.028563_TableS12.pdf]

Table S-12. All genome-wide significant marginal associations, asthma.

RSID	Chr	Position	A1	A0	Discovery, unadjusted		Discovery, adjusted		Replication, adjusted		Genome-wide sig.?	Replicated?	Annotation	Gene
					OR (95% CI)	P	OR (95% CI)	P	OR (95% CI)	P				
rs17612802	6	32620359	C	T	0.87 (0.83, 0.90)	6.01E-13	0.86 (0.83, 0.90)	2.46E-13	0.84 (0.76, 0.94)	1.81E-03	Yes	Yes		
rs9273304	6	32622923	G	A	0.87 (0.84, 0.90)	6.77E-13	0.87 (0.83, 0.90)	2.53E-13	0.84 (0.76, 0.93)	1.15E-03	Yes	Yes	D, Q, G,	HLA-DQB1
rs1063355	6	32627714	T	G	0.87 (0.84, 0.90)	7.07E-13	0.87 (0.83, 0.90)	2.73E-13	0.84 (0.76, 0.94)	1.83E-03	Yes	Yes	R, D, Q, G,	HLA-DQB1
b37:6:32624786	6	32624786	T	G	0.87 (0.83, 0.90)	8.19E-13	0.87 (0.83, 0.90)	3.47E-13	0.84 (0.76, 0.94)	1.53E-03	Yes	Yes	D, G,	HLA-DQB1
rs9272656	6	32608629	A	G	0.87 (0.84, 0.90)	1.82E-12	0.87 (0.83, 0.90)	5.71E-13	0.84 (0.75, 0.93)	1.02E-03	Yes	Yes	D, Q, G,	HLA-DQA1
rs9273215	6	32613712	G	A	0.87 (0.84, 0.90)	1.63E-12	0.87 (0.83, 0.90)	6.00E-13	0.84 (0.76, 0.93)	1.23E-03	Yes	Yes	D, G,	HLA-DQA1
rs17612583	6	32615470	G	A	0.87 (0.84, 0.90)	2.03E-12	0.87 (0.84, 0.90)	7.71E-13	0.84 (0.76, 0.93)	1.17E-03	Yes	Yes	D, G,	HLA-DQA1
rs17612576	6	32615458	G	A	0.87 (0.84, 0.91)	2.31E-12	0.87 (0.84, 0.90)	8.73E-13	0.84 (0.76, 0.94)	1.49E-03	Yes	Yes	D, G,	HLA-DQA1
rs9273021	6	32611725	A	G	0.87 (0.84, 0.91)	2.56E-12	0.87 (0.84, 0.90)	9.65E-13	0.84 (0.76, 0.93)	1.28E-03	Yes	Yes	D, G,	HLA-DQA1
rs9273514	6	32628538	G	T	0.87 (0.84, 0.91)	3.15E-12	0.87 (0.84, 0.90)	1.00E-12	0.85 (0.77, 0.94)	2.29E-03	Yes	Yes	D, Q, G,	HLA-DQB1
rs9273329	6	32623242	G	A	0.87 (0.84, 0.91)	2.99E-12	0.87 (0.84, 0.90)	1.14E-12	0.84 (0.76, 0.94)	1.32E-03	Yes	Yes	R, D, Q, G,	HLA-DQB1
rs17843605	6	32620391	G	T	0.87 (0.84, 0.91)	3.28E-12	0.87 (0.84, 0.90)	1.26E-12	0.84 (0.76, 0.93)	1.19E-03	Yes	Yes		
rs17612510	6	32614883	C	T	0.87 (0.84, 0.91)	3.72E-12	0.87 (0.84, 0.90)	1.31E-12	0.84 (0.76, 0.93)	1.30E-03	Yes	Yes	D, G,	HLA-DQA1
rs9274177	6	32630407	A	G	0.87 (0.84, 0.91)	8.89E-12	0.87 (0.84, 0.90)	2.21E-12	0.85 (0.77, 0.95)	2.74E-03	Yes	Yes	D, G,	HLA-DQB1
rs17843577	6	32615510	C	T	0.87 (0.84, 0.91)	6.77E-12	0.87 (0.84, 0.91)	2.48E-12	0.84 (0.76, 0.93)	1.12E-03	Yes	Yes	D, G,	HLA-DQA1
rs9271176	6	32578127	A	G	0.87 (0.84, 0.91)	3.81E-11	0.87 (0.83, 0.91)	1.59E-11	0.91 (0.82, 1.02)	1.14E-01	Yes	No	Q,	
rs9274684	6	32636785	T	C	1.12 (1.07, 1.16)	9.65E-09	1.13 (1.08, 1.17)	8.20E-10	1.09 (0.98, 1.20)	1.15E-01	Yes	No	D, Q, G,	HLA-DQB1
rs7755212	6	32441408	C	T	0.89 (0.85, 0.92)	6.55E-10	0.89 (0.85, 0.92)	9.47E-10	0.88 (0.79, 0.98)	1.68E-02	Yes	Yes	R, Q,	
rs9268893	6	32431927	C	T	0.89 (0.86, 0.92)	1.75E-09	0.89 (0.86, 0.92)	2.58E-09	0.89 (0.81, 0.99)	3.89E-02	Yes	Yes		
rs9268877	6	32431147	A	G	0.89 (0.86, 0.92)	2.13E-09	0.89 (0.86, 0.93)	2.95E-09	0.89 (0.80, 0.99)	3.27E-02	Yes	Yes	Q,	
rs5026743	6	32439964	G	T	0.89 (0.86, 0.93)	3.47E-09	0.89 (0.86, 0.93)	4.73E-09	0.89 (0.80, 0.99)	2.78E-02	Yes	Yes	Q,	
rs9275123	6	32650256	G	T	0.90 (0.87, 0.94)	9.41E-08	0.89 (0.86, 0.93)	4.93E-09	0.92 (0.84, 1.03)	1.56E-01	Yes	No		
rs9268979	6	32435044	T	C	0.89 (0.86, 0.93)	4.51E-09	0.89 (0.86, 0.93)	6.48E-09	0.88 (0.80, 0.98)	2.21E-02	Yes	Yes		
rs9275071	6	32648490	G	A	0.90 (0.87, 0.94)	1.33E-07	0.89 (0.86, 0.93)	7.30E-09	0.93 (0.84, 1.03)	1.83E-01	Yes	No		
rs4642516	6	32657543	G	T	0.90 (0.87, 0.94)	1.37E-07	0.89 (0.86, 0.93)	7.60E-09	0.92 (0.84, 1.03)	1.47E-01	Yes	No	R, Q,	
rs9275086	6	32648809	A	G	0.90 (0.87, 0.94)	1.39E-07	0.89 (0.86, 0.93)	7.66E-09	0.93 (0.84, 1.03)	1.67E-01	Yes	No		
rs7739357	6	32441641	A	G	0.89 (0.86, 0.93)	9.68E-09	0.89 (0.86, 0.93)	1.20E-08	0.89 (0.81, 1.00)	4.39E-02	Yes	Yes	R, Q,	
rs521539	6	32581973	A	G	1.14 (1.09, 1.20)	9.66E-09	1.14 (1.09, 1.20)	1.24E-08	1.11 (0.98, 1.26)	9.99E-02	Yes	No		
rs9274733	6	32637874	C	T	0.91 (0.87, 0.94)	2.38E-07	0.90 (0.86, 0.93)	1.25E-08	0.93 (0.85, 1.04)	2.10E-01	Yes	No	D, Q, G,	HLA-DQB1
rs9269080	6	32440969	G	A	0.89 (0.86, 0.93)	9.20E-09	0.90 (0.86, 0.93)	1.47E-08	0.90 (0.81, 1.00)	4.12E-02	Yes	Yes	Q,	
rs9275146	6	32651495	G	T	0.91 (0.87, 0.94)	2.69E-07	0.90 (0.86, 0.93)	1.47E-08	0.93 (0.84, 1.03)	1.73E-01	Yes	No		
rs9268833	6	32428062	T	C	1.12 (1.08, 1.17)	1.68E-08	1.12 (1.08, 1.17)	1.97E-08	1.17 (1.05, 1.31)	3.93E-03	Yes	Yes	R, Q,	
rs34965214	6	32609545	T	C	1.15 (1.09, 1.20)	1.35E-08	1.15 (1.09, 1.20)	2.19E-08	1.18 (1.04, 1.35)	9.88E-03	Yes	Yes	D, Q, G,	HLA-DQA1
rs9271544	6	32590120	A	C	1.12 (1.08, 1.17)	1.12E-08	1.12 (1.08, 1.17)	2.53E-08	1.12 (1.01, 1.26)	2.82E-02	Yes	Yes	Q,	
rs9271432	6	32588056	T	C	1.12 (1.08, 1.17)	9.81E-09	1.12 (1.08, 1.17)	2.55E-08	1.14 (1.03, 1.28)	1.27E-02	Yes	Yes		
rs660895	6	32577380	G	A	1.14 (1.09, 1.20)	2.26E-08	1.14 (1.09, 1.20)	2.71E-08	1.12 (0.98, 1.26)	1.06E-01	Yes	No	R, Q,	
rs9268835	6	32428115	A	G	1.12 (1.08, 1.17)	2.49E-08	1.12 (1.08, 1.17)	3.02E-08	1.16 (1.05, 1.30)	5.32E-03	Yes	Yes	Q,	
rs9271488	6	32589000	T	G	1.12 (1.08, 1.17)	1.30E-08	1.12 (1.08, 1.17)	3.10E-08	1.14 (1.03, 1.28)	1.44E-02	Yes	Yes		
rs3104371	6	32602137	G	C	1.15 (1.09, 1.20)	2.37E-08	1.14 (1.09, 1.20)	3.31E-08	1.16 (1.02, 1.32)	2.34E-02	Yes	Yes	D, Q, G,	HLA-DQA1
rs9272319	6	32604108	C	T	1.12 (1.08, 1.17)	1.47E-08	1.12 (1.08, 1.16)	3.43E-08	1.13 (1.02, 1.27)	2.07E-02	Yes	Yes	D, Q, G,	HLA-DQA1
rs9271170	6	32577889	T	C	0.89 (0.85, 0.93)	1.58E-07	0.89 (0.85, 0.92)	3.84E-08	0.92 (0.83, 1.04)	2.02E-01	Yes	No	R, Q,	

Table S-12. All genome-wide significant marginal associations, asthma (continued).

RSID	Chr	Position	A1	A0	Discovery, unadjusted		Discovery, adjusted		Replication, adjusted		Genome-wide sig.?	Replicated?	Annotation	Gene
					OR (95% CI)	P	OR (95% CI)	P	OR (95% CI)	P				
rs9271472	6	32588700	T	C	1.12 (1.08, 1.17)	1.59E-08	1.12 (1.08, 1.16)	3.85E-08	1.13 (1.02, 1.27)	1.82E-02	Yes	Yes		
rs9271769	6	32594217	T	G	1.12 (1.08, 1.17)	1.65E-08	1.12 (1.07, 1.16)	3.95E-08	1.13 (1.02, 1.27)	2.10E-02	Yes	Yes	Q,	
rs522308	6	32581922	T	C	1.12 (1.08, 1.17)	1.42E-08	1.12 (1.08, 1.17)	4.03E-08	1.17 (1.06, 1.32)	3.56E-03	Yes	Yes		
rs9273062	6	32612161	A	C	1.12 (1.08, 1.17)	1.71E-08	1.12 (1.07, 1.16)	4.32E-08	1.14 (1.03, 1.27)	1.60E-02	Yes	Yes	D, G,	HLA-DQA1
rs9271470	6	32588662	T	C	1.12 (1.08, 1.17)	1.89E-08	1.12 (1.07, 1.16)	4.64E-08	1.14 (1.03, 1.27)	1.59E-02	Yes	Yes		
rs1391371	6	32603798	T	A	1.14 (1.09, 1.20)	3.01E-08	1.14 (1.09, 1.20)	4.67E-08	1.19 (1.05, 1.35)	7.40E-03	Yes	Yes	D, Q, G,	HLA-DQA1
rs41269955	6	32608269	A	G	1.15 (1.09, 1.21)	2.63E-08	1.15 (1.09, 1.20)	4.80E-08	1.18 (1.03, 1.34)	1.84E-02	Yes	Yes	D, Q, G,	HLA-DQA1
rs9272485	6	32605972	G	A	1.12 (1.08, 1.17)	2.17E-08	1.12 (1.07, 1.16)	5.49E-08	1.13 (1.02, 1.27)	2.22E-02	Yes	Yes	R, D, Q, G,	HLA-DQA1
rs9270839	6	32570375	A	C	0.89 (0.85, 0.93)	1.84E-07	0.89 (0.85, 0.93)	5.62E-08	0.90 (0.81, 1.02)	1.14E-01	Yes	No	Q,	
rs9271101	6	32576493	C	T	0.89 (0.85, 0.93)	1.83E-07	0.89 (0.85, 0.93)	6.06E-08	0.92 (0.83, 1.05)	2.26E-01	Yes	No	Q,	
rs601945	6	32573415	G	A	1.15 (1.09, 1.21)	3.30E-08	1.15 (1.09, 1.20)	6.14E-08	1.18 (1.03, 1.34)	1.58E-02	Yes	Yes		
rs9270901	6	32571933	C	A	0.89 (0.85, 0.93)	1.91E-07	0.89 (0.85, 0.93)	6.26E-08	0.90 (0.81, 1.02)	1.19E-01	Yes	No	Q,	
rs9270850	6	32570717	G	A	0.89 (0.85, 0.93)	1.99E-07	0.89 (0.85, 0.93)	6.48E-08	0.91 (0.82, 1.04)	1.71E-01	Yes	No	Q,	
rs9270935	6	32572701	A	G	0.89 (0.85, 0.93)	2.15E-07	0.89 (0.85, 0.93)	6.98E-08	0.91 (0.82, 1.04)	1.75E-01	Yes	No	Q,	
rs73727334	6	32570667	A	G	0.89 (0.86, 0.93)	2.36E-07	0.89 (0.85, 0.93)	7.03E-08	0.91 (0.82, 1.03)	1.47E-01	Yes	No	Q,	
rs7649348	3	49772375	G	A	1.11 (1.07, 1.15)	3.49E-08	1.11 (1.07, 1.15)	7.66E-08	1.05 (0.95, 1.17)	2.98E-01	Yes	No	G,	IP6K1
rs9271092	6	32576296	A	G	0.89 (0.85, 0.93)	2.40E-07	0.89 (0.85, 0.93)	8.10E-08	0.91 (0.82, 1.04)	1.68E-01	Yes	No	Q,	
rs9271090	6	32576247	T	C	0.89 (0.86, 0.93)	2.44E-07	0.89 (0.85, 0.93)	8.12E-08	0.91 (0.82, 1.04)	1.68E-01	Yes	No	Q,	
rs9271644	6	32592337	A	G	1.12 (1.08, 1.17)	3.02E-08	1.12 (1.07, 1.16)	8.22E-08	1.14 (1.03, 1.29)	1.10E-02	Yes	Yes	Q,	
rs9270979	6	32573780	A	G	0.89 (0.86, 0.93)	2.61E-07	0.89 (0.85, 0.93)	8.71E-08	0.91 (0.81, 1.03)	1.31E-01	Yes	No		
rs9270949	6	32572975	T	C	0.89 (0.86, 0.93)	2.66E-07	0.89 (0.85, 0.93)	8.74E-08	0.91 (0.82, 1.04)	1.72E-01	Yes	No	Q,	
rs9271469	6	32588630	C	T	1.12 (1.08, 1.16)	3.61E-08	1.12 (1.07, 1.16)	8.87E-08	1.13 (1.02, 1.27)	2.06E-02	Yes	Yes		
rs9271198	6	32578726	G	A	0.89 (0.86, 0.93)	2.79E-07	0.89 (0.85, 0.93)	9.18E-08	0.92 (0.83, 1.05)	2.25E-01	Yes	No	Q,	
rs9270873	6	32571201	G	A	0.89 (0.86, 0.93)	2.78E-07	0.89 (0.85, 0.93)	9.21E-08	0.91 (0.82, 1.04)	1.68E-01	Yes	No	Q,	
rs9270933	6	32572659	C	G	0.89 (0.86, 0.93)	2.98E-07	0.89 (0.85, 0.93)	9.29E-08	0.91 (0.82, 1.04)	1.73E-01	Yes	No	Q,	
rs9271163	6	32577733	C	T	0.89 (0.86, 0.93)	2.58E-07	0.89 (0.85, 0.93)	9.41E-08	0.90 (0.81, 1.02)	1.21E-01	Yes	No	Q,	
rs9271109	6	32576687	T	C	0.89 (0.86, 0.93)	3.01E-07	0.89 (0.85, 0.93)	9.63E-08	0.91 (0.82, 1.03)	1.48E-01	Yes	No	Q,	
rs11709680	3	49745188	C	T	1.11 (1.07, 1.16)	4.81E-08	1.11 (1.07, 1.15)	9.83E-08	1.04 (0.94, 1.15)	4.76E-01	Yes	No	G,	RNF123
