# Supplementary material for: Exhaustive Genome-Wide Search for SNP-SNP Interactions Across 10 Human Diseases
Source: G3 (Bethesda). 2016 May 12;6(7):2043–50. doi: 10.1534/g3.116.028563 (PMC4938657; doi:10.1534/g3.116.028563)
Supplement: Supplemental Material [file supp_g3.116.028563_TableS13.pdf]

Table S-13. Top 10 most significant marginal associations, cardiac disease.

RSID	Chr	Position	A1	A0	Discovery, unadjusted		Discovery, adjusted		Replication, adjusted		Genome-wide sig.?	Replicated?	Annotation	Gene
					OR (95% CI)	P	OR (95% CI)	P	OR (95% CI)	P				
rs6843082	4	111718067	G	A	1.06 (1.02, 1.10)	4.57E-03	1.11 (1.07, 1.16)	7.41E-07	0.97 (0.86, 1.13)	8.51E-01	No	No		
rs7558074	2	99992822	A	C	0.94 (0.91, 0.97)	2.14E-04	0.92 (0.89, 0.95)	3.04E-06	0.93 (0.82, 1.03)	1.28E-01	No	No	G,	EIF5B
rs2723334	4	111688752	T	C	1.05 (1.01, 1.09)	1.36E-02	1.11 (1.06, 1.15)	3.36E-06	0.98 (0.87, 1.14)	9.59E-01	No	No		
rs6714244	2	100105312	A	G	0.94 (0.91, 0.97)	2.54E-04	0.92 (0.89, 0.95)	4.59E-06	0.93 (0.83, 1.03)	1.70E-01	No	No	R, G,	REV1
rs13236941	7	94955820	T	C	0.92 (0.88, 0.96)	9.01E-05	0.90 (0.85, 0.94)	5.24E-06	1.02 (0.85, 1.16)	9.45E-01	No	No	D, G,	PON1
rs2392991	10	57954930	A	C	1.08 (1.04, 1.11)	3.39E-06	1.09 (1.05, 1.12)	5.50E-06	0.92 (0.79, 1.00)	4.55E-02	No	No		
rs28628093	15	94333190	A	C	1.05 (1.02, 1.09)	1.39E-03	1.09 (1.05, 1.13)	5.55E-06	0.94 (0.83, 1.05)	2.78E-01	No	No	G,	BC037497
rs7903175	10	123105123	T	C	0.93 (0.90, 0.97)	1.16E-04	0.91 (0.88, 0.95)	5.90E-06	0.98 (0.87, 1.12)	8.61E-01	No	No		
rs2393047	10	58046350	G	A	1.07 (1.04, 1.11)	2.60E-05	1.09 (1.05, 1.12)	9.78E-06	0.96 (0.83, 1.05)	2.32E-01	No	No		
rs10225710	7	22142742	T	C	0.93 (0.90, 0.96)	1.00E-05	0.92 (0.89, 0.96)	1.00E-05	1.01 (0.92, 1.16)	6.05E-01	No	No		
