# Supplementary material for: Exhaustive Genome-Wide Search for SNP-SNP Interactions Across 10 Human Diseases
Source: G3 (Bethesda). 2016 May 12;6(7):2043–50. doi: 10.1534/g3.116.028563 (PMC4938657; doi:10.1534/g3.116.028563)
Supplement: Supplemental Material [file supp_g3.116.028563_TableS14.pdf]

Table S-14. Top 10 most significant marginal associations, depression.

RSID	Chr	Position	A1	A0	Discovery, unadjusted		Discovery, adjusted		Replication, adjusted		Genome-wide sig.?	Replicated?	Annotation	Gene
					OR (95% CI)	P	OR (95% CI)	P	OR (95% CI)	P				
rs7797095	7	86567364	T	C	1.13 (1.07, 1.19)	1.12E-05	1.14 (1.08, 1.20)	4.11E-06	0.94 (0.82, 1.09)	4.18E-01	No	No	G,	KIAA1324L
rs12704297	7	86546982	C	T	1.13 (1.07, 1.19)	1.66E-05	1.14 (1.07, 1.20)	5.96E-06	N/A	N/A	No	No	G,	KIAA1324L
rs2396431	2	227765488	A	G	1.10 (1.05, 1.15)	2.06E-05	1.10 (1.06, 1.15)	8.87E-06	1.01 (0.89, 1.10)	8.82E-01	No	No	G,	RHBDD1
rs2217359	2	227759892	T	C	1.10 (1.05, 1.15)	2.21E-05	1.10 (1.06, 1.15)	9.85E-06	1.00 (0.88, 1.09)	7.20E-01	No	No	G,	RHBDD1
rs11933869	4	161763436	A	G	0.88 (0.83, 0.93)	3.24E-05	0.87 (0.82, 0.93)	1.19E-05	1.06 (0.90, 1.20)	5.83E-01	No	No		
rs4675115	2	227769123	G	C	1.10 (1.05, 1.14)	3.79E-05	1.10 (1.06, 1.15)	1.25E-05	N/A	N/A	No	No	G,	RHBDD1
rs7592387	2	227777095	G	A	1.10 (1.05, 1.14)	3.43E-05	1.10 (1.05, 1.15)	1.27E-05	0.99 (0.88, 1.09)	6.67E-01	No	No	G,	RHBDD1
rs2396432	2	227765891	T	C	1.10 (1.05, 1.14)	3.17E-05	1.10 (1.05, 1.15)	1.40E-05	1.01 (0.89, 1.10)	8.57E-01	No	No	R, G,	RHBDD1
rs10933161	2	227764872	G	A	1.10 (1.05, 1.14)	3.39E-05	1.10 (1.05, 1.15)	1.46E-05	1.01 (0.90, 1.11)	9.54E-01	No	No	G,	RHBDD1
rs3820928	2	227773466	C	T	1.10 (1.05, 1.14)	3.98E-05	1.10 (1.05, 1.15)	1.47E-05	1.00 (0.88, 1.09)	7.26E-01	No	No	R, G,	RHBDD1
