# Supplementary material for: Exhaustive Genome-Wide Search for SNP-SNP Interactions Across 10 Human Diseases
Source: G3 (Bethesda). 2016 May 12;6(7):2043–50. doi: 10.1534/g3.116.028563 (PMC4938657; doi:10.1534/g3.116.028563)
Supplement: Supplemental Material [file supp_g3.116.028563_TableS15.pdf]

Table S-15. Top 10 most significant marginal associations, dermatophytosis.

RSID	Chr	Position	A1	A0	Discovery, unadjusted		Discovery, adjusted		Replication, adjusted		Genome-wide sig.?	Replicated?	Annotation	Gene
					OR (95% CI)	P	OR (95% CI)	P	OR (95% CI)	P				
rs35626362	4	121583673	T	C	0.90 (0.86, 0.94)	2.43E-05	0.89 (0.85, 0.94)	1.12E-05	1.05 (0.93, 1.19)	3.92E-01	No	No		
rs34769609	4	121551435	C	-	0.90 (0.86, 0.94)	2.51E-05	0.89 (0.85, 0.94)	1.25E-05	1.05 (0.94, 1.19)	3.82E-01	No	No		
rs2072542	12	22440315	G	A	1.11 (1.06, 1.15)	1.21E-06	1.10 (1.05, 1.15)	1.32E-05	1.01 (0.91, 1.12)	8.98E-01	No	No	G,	ST8SIA1
rs908132	2	102093096	A	G	0.92 (0.88, 0.96)	7.89E-05	0.91 (0.87, 0.95)	1.82E-05	0.97 (0.86, 1.07)	5.03E-01	No	No	G,	RFX8
rs35513623	4	121579530	T	C	0.90 (0.86, 0.95)	4.08E-05	0.90 (0.85, 0.94)	1.97E-05	1.05 (0.93, 1.18)	4.26E-01	No	No		
rs6814315	4	39793385	T	G	1.09 (1.05, 1.14)	2.77E-05	1.10 (1.05, 1.14)	2.40E-05	1.01 (0.92, 1.12)	7.76E-01	No	No		
rs4805005	19	28468556	A	G	1.15 (1.09, 1.21)	3.66E-07	1.12 (1.06, 1.18)	2.41E-05	0.96 (0.83, 1.10)	5.32E-01	No	No		
rs433223	21	24423707	A	G	1.09 (1.05, 1.14)	5.45E-05	1.10 (1.05, 1.14)	2.42E-05	1.08 (0.97, 1.21)	1.43E-01	No	No		
rs4762904	12	22478425	A	G	1.10 (1.05, 1.14)	9.93E-06	1.09 (1.05, 1.14)	3.13E-05	1.04 (0.94, 1.16)	4.10E-01	No	No	G,	ST8SIA1
rs7728254	5	6810950	A	G	1.08 (1.04, 1.13)	1.39E-04	1.09 (1.05, 1.14)	3.32E-05	1.01 (0.91, 1.12)	8.53E-01	No	No		
