# Supplementary material for: Exhaustive Genome-Wide Search for SNP-SNP Interactions Across 10 Human Diseases
Source: G3 (Bethesda). 2016 May 12;6(7):2043–50. doi: 10.1534/g3.116.028563 (PMC4938657; doi:10.1534/g3.116.028563)
Supplement: Supplemental Material [file supp_g3.116.028563_TableS16.pdf]

Table S-16. All genome-wide significant marginal associations, type 2 diabetes.

RSID	Chr	Position	A1	A0	Discovery, unadjusted		Discovery, adjusted		Replication, adjusted		Genome-wide sig.?	Replicated?	Annotation	Gene
					OR (95% CI)	P	OR (95% CI)	P	OR (95% CI)	P				
rs4506565	10	114756041	T	A	1.35 (1.29, 1.41)	1.55E-37	1.35 (1.28, 1.41)	3.22E-36	1.31 (1.17, 1.46)	1.92E-06	Yes	Yes	D, G,	TCF7L2
rs4132670	10	114767771	A	G	1.34 (1.28, 1.40)	1.96E-36	1.34 (1.28, 1.40)	7.71E-35	1.32 (1.18, 1.47)	1.40E-06	Yes	Yes	D, G,	TCF7L2
rs55899248	10	114773608	G	A	1.34 (1.28, 1.41)	2.08E-31	1.35 (1.28, 1.42)	3.82E-31	1.26 (1.09, 1.39)	8.38E-04	Yes	Yes	D, G,	TCF7L2
rs12255372	10	114808902	T	G	1.30 (1.24, 1.36)	2.03E-28	1.30 (1.24, 1.36)	9.24E-27	1.32 (1.16, 1.46)	4.03E-06	Yes	Yes	D, G,	TCF7L2
rs61872786	10	114806697	A	G	1.30 (1.23, 1.36)	1.61E-23	1.30 (1.23, 1.37)	5.54E-23	1.26 (1.10, 1.40)	6.74E-04	Yes	Yes	D, G,	TCF7L2
rs7899529	10	114779538	A	G	1.20 (1.15, 1.25)	2.21E-16	1.20 (1.15, 1.25)	8.41E-16	1.17 (1.05, 1.30)	3.35E-03	Yes	Yes	D, G,	TCF7L2
rs11196191	10	114780633	C	A	1.20 (1.15, 1.25)	5.29E-16	1.20 (1.15, 1.25)	1.34E-15	1.16 (1.04, 1.28)	6.46E-03	Yes	Yes	D, G,	TCF7L2
rs61872790	10	114821527	G	A	1.23 (1.16, 1.30)	1.33E-12	1.24 (1.17, 1.31)	6.84E-13	1.25 (1.08, 1.43)	2.32E-03	Yes	Yes	D, G,	TCF7L2
rs12255678	10	114729482	G	T	1.17 (1.11, 1.23)	4.18E-10	1.17 (1.11, 1.23)	1.42E-09	1.07 (0.92, 1.18)	4.95E-01	Yes	No	R, D, G,	TCF7L2
rs2391591	7	28157664	G	T	1.14 (1.09, 1.19)	1.36E-08	1.14 (1.09, 1.20)	7.46E-09	1.00 (0.89, 1.10)	8.10E-01	Yes	No	D, G,	JAZF1
rs6962289	7	28161379	C	A	1.14 (1.09, 1.19)	1.13E-08	1.14 (1.09, 1.20)	7.48E-09	1.00 (0.89, 1.10)	8.21E-01	Yes	No	D, G,	JAZF1
rs13266634	8	118184783	T	C	0.87 (0.83, 0.91)	2.46E-08	0.87 (0.83, 0.91)	2.92E-08	0.95 (0.85, 1.07)	3.79E-01	Yes	No	EX, D, G,	SLC30A8
rs849142	7	28185891	T	C	1.13 (1.08, 1.18)	7.03E-08	1.13 (1.08, 1.18)	3.18E-08	1.09 (0.98, 1.21)	9.91E-02	Yes	No	D, G,	JAZF1
rs864745	7	28180556	T	C	1.13 (1.08, 1.18)	9.04E-08	1.13 (1.08, 1.18)	4.27E-08	1.10 (0.98, 1.21)	1.01E-01	Yes	No	D, G,	JAZF1
rs1635852	7	28189411	T	C	1.13 (1.08, 1.18)	9.51E-08	1.13 (1.08, 1.18)	4.62E-08	1.09 (0.99, 1.21)	9.33E-02	Yes	No	R, D, G,	JAZF1
rs849135	7	28196413	A	G	0.89 (0.85, 0.93)	1.19E-07	0.88 (0.85, 0.92)	5.39E-08	0.90 (0.81, 1.00)	5.48E-02	Yes	No	D, G,	JAZF1
rs1708302	7	28198677	T	C	0.89 (0.85, 0.93)	1.32E-07	0.88 (0.85, 0.92)	6.01E-08	0.90 (0.81, 1.00)	5.38E-02	Yes	No	D, G,	JAZF1
rs6801848	3	185499057	G	C	1.13 (1.08, 1.18)	5.17E-07	1.14 (1.09, 1.20)	9.58E-08	1.10 (0.98, 1.24)	8.99E-02	Yes	No	D, G,	IGF2BP2
