# Supplementary material for: Exhaustive Genome-Wide Search for SNP-SNP Interactions Across 10 Human Diseases
Source: G3 (Bethesda). 2016 May 12;6(7):2043–50. doi: 10.1534/g3.116.028563 (PMC4938657; doi:10.1534/g3.116.028563)
Supplement: Supplemental Material [file supp_g3.116.028563_FigureS2.pdf]

Figure S-2. Comparison of epistasis analysis results derived from Plink 1.07 and Plink 1.90.

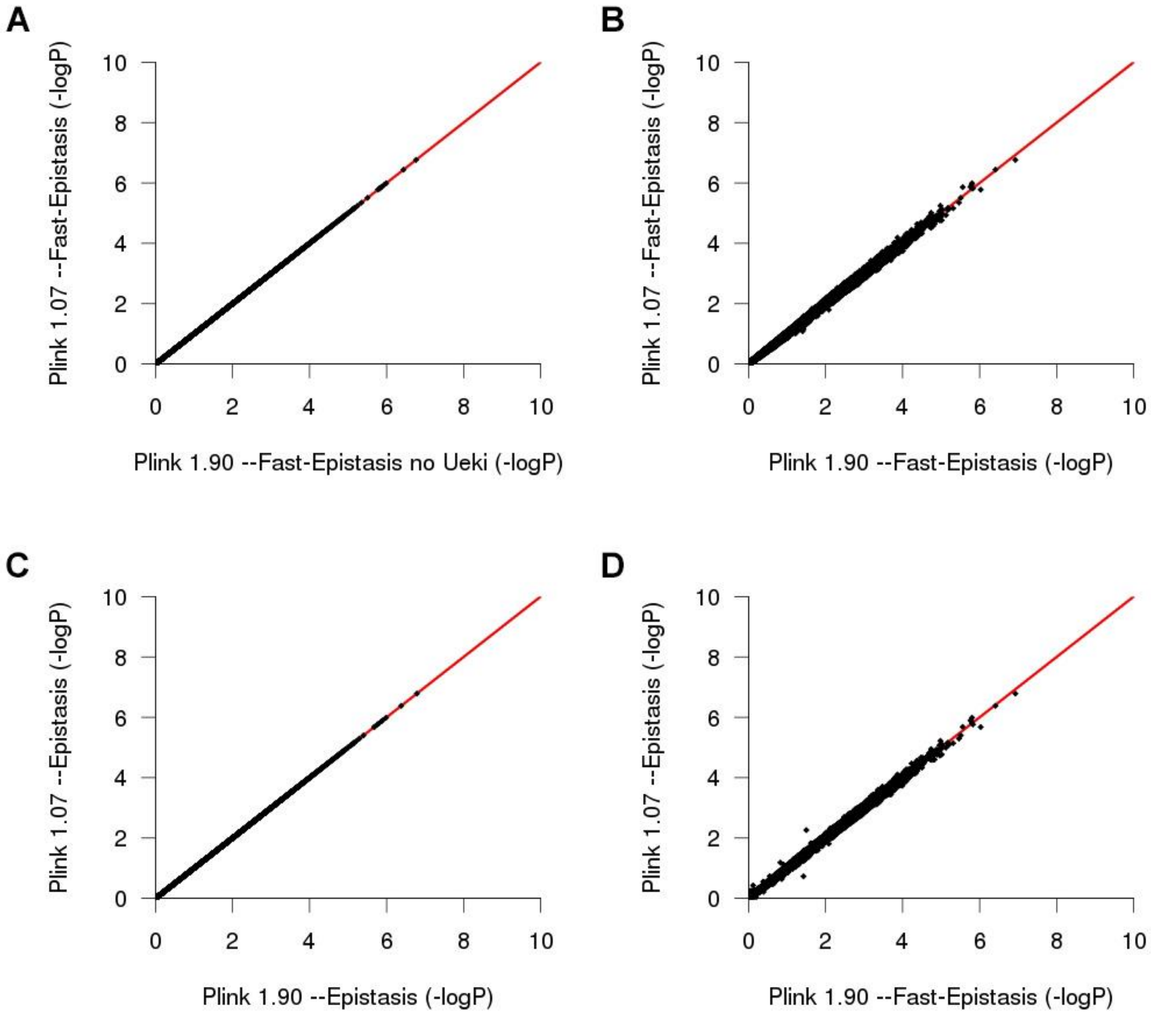

Since Plink 1.90 (the software used to conduct genome-wide SNP-SNP interaction analyses in the present study) was in beta testing at the time of study, we sought to verify the accuracy of its results. To do this, we compared four different analyses conducted in Plink 1.90 to similar analyses conducted in Plink 1.07 (the latest stable release of the original Plink toolset), as follows: **(A)** Plink 1.07 FastEpistasis vs. Plink 1.90 FastEpistasis with the "no Ueki" option. This option disables corrections made to the FastEpistasis algorithm implemented in Plink 1.90, in order to allow exact comparisons with FastEpistasis as implemented in Plink 1.07 (see https://www.cog-genomics.org/plink2/epistasis). **(B)** Plink 1.07 FastEpistasis vs. Plink 1.90 FastEpistasis without the "No Ueki" option. Slight differences in results from the two packages are due to known corrections made using the Ueki method. **(C)** Plink 1.07 Epistasis vs. Plink 1.90 Epistasis. Here, both packages used logistic regression to evaluate epistatic effects. **(D)** Plink 1.07 Epistasis vs. Plink 1.90 FastEpistasis. Slight differences are due to the known fact that FastEpistasis is designed as an approximate method to search for epistatic effects. To conduct all of these comparisons, N = 2,012 SNPs were randomly selected from the discovery dataset (for type 2 diabetes) and all possible combinations of these SNPs were subjected to the various analyses for SNP-SNP interactions (N = 2,023,066 interactions tested for each analysis). –Log10 P-values from these tests are plotted above. Red lines indicate 1:1 correlation.

References:

Package: Plink 1.90 beta. Authors: Shaun Purcell, Christopher Chang URL: https://www.cog-genomics.org/plink2
Chang CC, Chow CC, Tellier LCAM, Vattikuti S, Purcell SM, Lee JJ (2015) Second-generation PLINK: rising to the challenge of larger and richer datasets. GigaScience, 4.

Package: Plink 1.07. Authors: Shaun Purcell. URL: http://pngu.mgh.harvard.edu/purcell/plink/
Purcell S, Neale B, Todd-Brown K, Thomas L, Ferreira MAR, Bender D, Maller J, Sklar P, de Bakker PIW, Daly MJ & Sham PC (2007). PLINK: a toolset for whole-genome association and population-based linkage analysis. American Journal of Human Genetics, 81.
