# Supplementary material for: Exhaustive Genome-Wide Search for SNP-SNP Interactions Across 10 Human Diseases
Source: G3 (Bethesda). 2016 May 12;6(7):2043–50. doi: 10.1534/g3.116.028563 (PMC4938657; doi:10.1534/g3.116.028563)
Supplement: Supplemental Material [file supp_g3.116.028563_TableS17.pdf]

Table S-17. All genome-wide significant marginal associations, dyslipidaemia.

RSID	Chr	Position	A1	A0	Discovery, unadjusted		Discovery, adjusted		Replication, adjusted		Genome-wide sig.?	Replicated?	Annotation	Gene
					OR (95% CI)	P	OR (95% CI)	P	OR (95% CI)	P				
rs1367117	2	21263900	A	G	1.19 (1.16, 1.23)	4.18E-30	1.24 (1.20, 1.28)	1.83E-37	1.06 (0.94, 1.19)	3.41E-01	Yes	No	EX, D, G,	APOB
rs1469513	2	21259562	C	T	1.17 (1.14, 1.20)	6.95E-27	1.20 (1.16, 1.23)	9.38E-31	1.09 (0.98, 1.22)	9.59E-02	Yes	No	D, G,	APOB
rs7575840	2	21273490	T	G	1.17 (1.14, 1.21)	2.92E-25	1.21 (1.17, 1.25)	9.65E-31	1.08 (0.97, 1.22)	1.40E-01	Yes	No		
rs10199768	2	21244000	T	G	1.16 (1.13, 1.19)	3.36E-24	1.19 (1.15, 1.22)	1.10E-28	1.10 (0.99, 1.23)	6.47E-02	Yes	No	D, G,	APOB
rs17395512	2	21346556	C	T	1.16 (1.12, 1.19)	1.28E-20	1.19 (1.15, 1.23)	4.07E-26	1.06 (0.96, 1.20)	2.33E-01	Yes	No		
rs9305020	19	11186711	C	T	0.83 (0.80, 0.86)	2.39E-24	0.81 (0.78, 0.84)	5.34E-26	0.83 (0.70, 0.92)	2.28E-03	Yes	Yes		
rs952275	2	21221399	G	T	1.14 (1.11, 1.17)	7.76E-20	1.17 (1.14, 1.21)	2.31E-25	1.16 (1.06, 1.31)	3.17E-03	Yes	Yes	D, G,	APOB
rs693	2	21232195	A	G	1.14 (1.11, 1.17)	4.02E-20	1.17 (1.14, 1.21)	2.32E-25	1.16 (1.06, 1.31)	3.26E-03	Yes	Yes	D, G,	APOB
rs1041968	2	21232804	A	G	1.14 (1.11, 1.17)	2.17E-19	1.17 (1.14, 1.21)	1.17E-24	1.19 (1.07, 1.33)	1.28E-03	Yes	Yes	D, G,	APOB
rs562338	2	21288321	A	G	0.85 (0.82, 0.88)	3.49E-18	0.82 (0.79, 0.86)	3.27E-23	0.84 (0.72, 0.94)	5.22E-03	Yes	Yes		
rs515135	2	21286057	T	C	0.86 (0.83, 0.89)	1.76E-17	0.83 (0.80, 0.86)	1.00E-22	0.83 (0.71, 0.94)	3.65E-03	Yes	Yes		
rs541041	2	21294975	G	A	0.86 (0.83, 0.89)	1.53E-17	0.83 (0.80, 0.86)	5.24E-22	0.83 (0.72, 0.95)	5.93E-03	Yes	Yes		
rs13392272	2	21217490	T	C	1.13 (1.10, 1.16)	3.07E-17	1.16 (1.12, 1.19)	3.31E-21	1.16 (1.04, 1.29)	5.75E-03	Yes	Yes		
rs481069	2	21281856	G	A	0.86 (0.83, 0.89)	7.62E-15	0.82 (0.79, 0.86)	3.89E-20	0.82 (0.69, 0.93)	3.70E-03	Yes	Yes		
rs62122481	2	21216815	A	C	1.12 (1.09, 1.16)	7.66E-15	1.16 (1.12, 1.19)	1.07E-19	1.14 (1.02, 1.28)	2.08E-02	Yes	Yes		
rs10808546	8	126495818	T	C	0.88 (0.86, 0.91)	9.49E-19	0.87 (0.84, 0.90)	1.28E-19	0.90 (0.80, 1.00)	4.33E-02	Yes	Yes		
rs12477249	2	21400661	A	G	1.12 (1.09, 1.16)	1.64E-14	1.16 (1.12, 1.19)	2.08E-19	1.03 (0.91, 1.15)	6.75E-01	Yes	No		
rs7567217	2	21303470	C	T	0.86 (0.83, 0.90)	1.17E-13	0.83 (0.80, 0.86)	1.16E-18	0.81 (0.69, 0.93)	3.18E-03	Yes	Yes		
rs949790	2	21449987	A	G	1.13 (1.09, 1.17)	1.09E-12	1.17 (1.13, 1.22)	1.47E-18	1.07 (0.95, 1.22)	2.57E-01	Yes	No		
rs2980871	8	126488930	G	A	0.88 (0.86, 0.91)	8.66E-18	0.87 (0.85, 0.90)	2.04E-18	0.89 (0.80, 0.99)	3.78E-02	Yes	Yes		
rs2954031	8	126491733	T	G	0.89 (0.86, 0.91)	3.91E-17	0.87 (0.85, 0.90)	3.27E-18	0.89 (0.80, 0.99)	2.85E-02	Yes	Yes		
rs614303	2	21313577	G	A	0.88 (0.85, 0.91)	5.27E-14	0.85 (0.82, 0.88)	4.29E-18	0.84 (0.73, 0.95)	6.15E-03	Yes	Yes		
rs506585	2	21397182	G	A	0.87 (0.84, 0.90)	1.88E-14	0.85 (0.82, 0.88)	4.55E-18	0.83 (0.72, 0.94)	4.38E-03	Yes	Yes		
rs2001945	8	126477978	G	C	1.13 (1.10, 1.16)	2.28E-17	1.14 (1.11, 1.18)	4.61E-18	NA (0.95, 1.18)	3.11E-01	Yes	No		
rs6982502	8	126479362	C	T	1.13 (1.10, 1.16)	3.47E-17	1.14 (1.11, 1.18)	4.75E-18	1.06 (0.95, 1.17)	3.21E-01	Yes	No		
rs478442	2	21399216	G	T	0.87 (0.84, 0.91)	3.27E-14	0.85 (0.82, 0.88)	8.17E-18	0.84 (0.73, 0.94)	4.89E-03	Yes	Yes		
rs544039	2	21398985	C	A	0.87 (0.84, 0.91)	3.26E-14	0.85 (0.82, 0.88)	8.95E-18	NA (0.73, 0.95)	5.17E-03	Yes	Yes	R,	
rs1712251	2	21396551	G	A	0.88 (0.85, 0.91)	5.19E-14	0.85 (0.82, 0.88)	9.11E-18	0.83 (0.72, 0.94)	4.35E-03	Yes	Yes		
rs560408	2	21398379	T	C	0.88 (0.85, 0.91)	3.87E-14	0.85 (0.82, 0.88)	9.68E-18	NA (0.72, 0.94)	4.40E-03	Yes	Yes		
rs17321515	8	126486409	G	A	0.89 (0.86, 0.91)	3.31E-17	0.88 (0.85, 0.90)	1.16E-17	0.88 (0.80, 0.99)	3.34E-02	Yes	Yes		
rs574461	2	21400013	A	G	0.88 (0.85, 0.91)	5.67E-14	0.85 (0.82, 0.88)	1.25E-17	0.84 (0.73, 0.94)	4.68E-03	Yes	Yes		
rs578095	2	21393866	A	G	0.87 (0.85, 0.91)	4.44E-14	0.85 (0.82, 0.88)	1.26E-17	0.84 (0.73, 0.95)	5.72E-03	Yes	Yes		
rs1652420	2	21383881	A	T	0.88 (0.85, 0.91)	8.29E-14	0.85 (0.82, 0.88)	1.32E-17	0.85 (0.74, 0.96)	9.96E-03	Yes	Yes		
rs531380	2	21397561	C	G	0.88 (0.85, 0.91)	6.27E-14	0.85 (0.82, 0.88)	1.35E-17	NA (0.73, 0.95)	5.18E-03	Yes	Yes		
rs1712247	2	21383951	C	T	0.88 (0.85, 0.91)	5.65E-14	0.85 (0.82, 0.88)	1.45E-17	0.84 (0.73, 0.95)	7.06E-03	Yes	Yes		
rs522250	2	21387113	T	C	0.88 (0.84, 0.91)	6.52E-14	0.85 (0.82, 0.88)	1.45E-17	0.84 (0.73, 0.95)	6.23E-03	Yes	Yes		
rs1652416	2	21396609	A	G	0.88 (0.85, 0.91)	5.58E-14	0.85 (0.82, 0.88)	1.45E-17	0.83 (0.72, 0.94)	3.89E-03	Yes	Yes		
rs483436	2	21390407	G	A	0.88 (0.85, 0.91)	6.48E-14	0.85 (0.82, 0.88)	1.47E-17	0.84 (0.73, 0.95)	5.96E-03	Yes	Yes		
rs494465	2	21401010	C	T	0.88 (0.85, 0.91)	7.69E-14	0.85 (0.82, 0.88)	1.64E-17	0.84 (0.72, 0.94)	4.37E-03	Yes	Yes		
rs480787	2	21390169	A	G	0.88 (0.85, 0.91)	7.12E-14	0.85 (0.82, 0.88)	1.71E-17	0.84 (0.73, 0.95)	6.68E-03	Yes	Yes		
rs560844	2	21389108	A	G	0.88 (0.85, 0.91)	5.92E-14	0.85 (0.82, 0.88)	1.78E-17	0.84 (0.73, 0.95)	6.30E-03	Yes	Yes		
rs532225	2	21397637	T	C	0.88 (0.85, 0.91)	7.68E-14	0.85 (0.82, 0.88)	1.79E-17	0.84 (0.73, 0.95)	8.05E-03	Yes	Yes		
rs573314	2	21385161	C	G	0.88 (0.85, 0.91)	7.49E-14	0.85 (0.82, 0.88)	2.04E-17	0.84 (0.73, 0.95)	5.08E-03	Yes	Yes		
rs554414	2	21394892	T	C	0.88 (0.85, 0.91)	8.47E-14	0.85 (0.82, 0.88)	2.07E-17	0.84 (0.73, 0.95)	5.94E-03	Yes	Yes		

Table S-17. All genome-wide significant marginal associations, dyslipidaemia (continued).

RSID	Chr	Position	A1	A0	Discovery, unadjusted		Discovery, adjusted		Replication, adjusted		Genome-wide sig.?	Replicated?	Annotation	Gene
					OR (95% CI)	P	OR (95% CI)	P	OR (95% CI)	P				
rs494315	2	21400950	C	T	0.88 (0.85, 0.91)	7.80E-14	0.85 (0.82, 0.88)	2.15E-17	0.83 (0.72, 0.94)	3.88E-03	Yes	Yes		
rs570033	2	21399567	C	G	0.88 (0.85, 0.91)	8.78E-14	0.85 (0.82, 0.88)	2.19E-17	0.83 (0.72, 0.94)	3.93E-03	Yes	Yes		
rs558342	2	21395469	G	A	0.88 (0.85, 0.91)	1.73E-13	0.85 (0.82, 0.89)	2.21E-17	0.84 (0.73, 0.95)	5.04E-03	Yes	Yes		
rs486246	2	21406751	G	A	0.88 (0.85, 0.91)	7.92E-14	0.85 (0.82, 0.88)	2.40E-17	0.84 (0.73, 0.95)	6.95E-03	Yes	Yes		
rs548506	2	21385541	A	G	0.88 (0.85, 0.91)	8.37E-14	0.85 (0.82, 0.88)	2.60E-17	0.84 (0.73, 0.95)	6.13E-03	Yes	Yes		
rs1652418	2	21388456	T	C	0.88 (0.85, 0.91)	9.40E-14	0.85 (0.82, 0.88)	2.75E-17	0.83 (0.72, 0.93)	2.77E-03	Yes	Yes		
rs538528	2	21391740	T	C	0.88 (0.85, 0.91)	9.83E-14	0.85 (0.82, 0.88)	2.83E-17	0.84 (0.72, 0.94)	4.02E-03	Yes	Yes		
rs3741298	11	116657561	C	T	1.16 (1.12, 1.20)	4.20E-17	1.17 (1.13, 1.22)	2.88E-17	1.04 (0.92, 1.19)	5.03E-01	Yes	No	D, G,	ZNF259
rs4591370	2	21383742	A	G	0.88 (0.85, 0.91)	1.31E-13	0.85 (0.82, 0.88)	2.89E-17	0.85 (0.74, 0.96)	8.96E-03	Yes	Yes		
rs2954019	8	126477759	A	G	0.89 (0.86, 0.91)	2.91E-16	0.88 (0.85, 0.91)	3.15E-17	0.90 (0.81, 1.00)	5.59E-02	Yes	No		
rs2980856	8	126476379	G	C	0.89 (0.86, 0.91)	1.81E-16	0.88 (0.85, 0.91)	3.16E-17	NA (0.81, 1.01)	7.56E-02	Yes	No	R,	
rs501863	2	21390000	G	A	0.88 (0.85, 0.91)	1.06E-13	0.85 (0.82, 0.89)	3.20E-17	0.84 (0.73, 0.95)	6.56E-03	Yes	Yes		
rs529697	2	21387948	G	T	0.88 (0.85, 0.91)	1.56E-13	0.85 (0.82, 0.89)	3.40E-17	0.84 (0.72, 0.94)	4.52E-03	Yes	Yes		
rs540439	2	21391978	C	T	0.88 (0.85, 0.91)	1.43E-13	0.85 (0.82, 0.89)	3.58E-17	0.84 (0.73, 0.95)	5.82E-03	Yes	Yes		
rs6982636	8	126479315	A	G	0.89 (0.86, 0.91)	2.27E-16	0.88 (0.85, 0.91)	3.90E-17	0.89 (0.81, 1.00)	5.74E-02	Yes	No	R,	
rs568938	2	21303616	C	T	0.88 (0.85, 0.91)	1.51E-13	0.85 (0.82, 0.89)	4.15E-17	0.83 (0.73, 0.95)	5.52E-03	Yes	Yes		
rs541569	2	21398768	G	A	0.88 (0.85, 0.91)	1.70E-13	0.85 (0.82, 0.89)	4.35E-17	0.84 (0.72, 0.94)	4.39E-03	Yes	Yes		
rs561850	2	21395805	A	G	0.88 (0.85, 0.91)	1.32E-13	0.85 (0.82, 0.89)	4.39E-17	0.84 (0.73, 0.94)	5.04E-03	Yes	Yes		
rs2980855	8	126477476	C	T	0.89 (0.86, 0.91)	2.67E-16	0.88 (0.85, 0.91)	4.40E-17	0.91 (0.82, 1.02)	9.64E-02	Yes	No		
rs5002500	2	21399974	C	G	0.88 (0.85, 0.91)	1.32E-13	0.85 (0.82, 0.88)	4.51E-17	0.84 (0.73, 0.96)	8.43E-03	Yes	Yes		
rs4560142	2	21383717	C	T	0.88 (0.85, 0.91)	1.24E-13	0.85 (0.82, 0.89)	4.97E-17	0.83 (0.72, 0.94)	3.62E-03	Yes	Yes		
rs312970	2	21369815	A	T	0.88 (0.85, 0.91)	2.50E-13	0.85 (0.82, 0.89)	5.83E-17	NA (0.73, 0.95)	6.14E-03	Yes	Yes		
rs480732	2	21390149	A	T	0.88 (0.85, 0.91)	2.60E-13	0.85 (0.82, 0.89)	7.31E-17	NA (0.73, 0.95)	5.25E-03	Yes	Yes		
rs312985	2	21378805	A	G	0.88 (0.85, 0.91)	3.88E-13	0.86 (0.82, 0.89)	9.09E-17	NA (0.73, 0.95)	5.65E-03	Yes	Yes		
rs477146	2	21396334	A	G	0.88 (0.85, 0.91)	3.15E-13	0.85 (0.82, 0.89)	9.98E-17	0.84 (0.73, 0.95)	8.18E-03	Yes	Yes		
rs527034	2	21382786	C	A	0.88 (0.85, 0.91)	9.13E-13	0.85 (0.82, 0.89)	1.08E-16	0.85 (0.73, 0.96)	8.62E-03	Yes	Yes		
rs2001844	8	126478745	G	A	0.89 (0.87, 0.92)	1.12E-15	0.88 (0.86, 0.91)	1.37E-16	0.89 (0.81, 1.00)	5.45E-02	Yes	No	R,	
rs58443763	8	126480526	T	C	0.89 (0.87, 0.92)	1.06E-15	0.88 (0.86, 0.91)	1.79E-16	0.90 (0.81, 1.01)	6.29E-02	Yes	No		
rs2954022	8	126482621	A	C	0.89 (0.87, 0.92)	1.08E-15	0.88 (0.86, 0.91)	1.80E-16	0.90 (0.81, 1.01)	6.78E-02	Yes	No		
rs2980875	8	126481747	G	A	0.89 (0.87, 0.92)	1.56E-15	0.88 (0.86, 0.91)	2.84E-16	0.90 (0.81, 1.00)	5.98E-02	Yes	No		
rs576203	2	21393623	A	G	0.88 (0.85, 0.91)	3.68E-12	0.86 (0.83, 0.89)	1.76E-15	0.85 (0.74, 0.97)	1.57E-02	Yes	Yes		
rs7605304	2	21445569	A	G	1.13 (1.09, 1.17)	5.47E-11	1.17 (1.13, 1.22)	2.64E-15	1.10 (0.96, 1.27)	1.67E-01	Yes	No		
rs2927472	19	45349369	T	C	0.88 (0.85, 0.91)	9.93E-11	0.85 (0.81, 0.88)	6.68E-15	0.71 (0.59, 0.81)	5.45E-06	Yes	Yes	R, G,	PVRL2
rs3846662	5	74651084	G	A	1.11 (1.08, 1.14)	2.09E-12	1.12 (1.08, 1.15)	5.50E-13	1.12 (1.01, 1.25)	2.76E-02	Yes	Yes	D, G,	HMGCR
rs520354	2	21259612	G	A	0.90 (0.87, 0.93)	2.76E-13	0.90 (0.87, 0.92)	8.32E-13	1.00 (0.89, 1.10)	8.84E-01	Yes	No	D, G,	APOB
rs365946	2	21439329	T	G	0.89 (0.86, 0.93)	1.04E-09	0.87 (0.84, 0.90)	1.14E-12	0.81 (0.71, 0.95)	6.10E-03	Yes	Yes		
rs7846466	8	126485531	T	C	1.12 (1.09, 1.16)	1.27E-13	1.12 (1.09, 1.16)	3.37E-12	1.04 (0.94, 1.19)	3.65E-01	Yes	No		
rs12916	5	74656539	C	T	1.10 (1.07, 1.14)	1.98E-11	1.11 (1.08, 1.15)	3.74E-12	1.15 (1.04, 1.30)	6.06E-03	Yes	Yes	D, G,	HMGCR
rs97458	2	21327635	G	A	0.91 (0.89, 0.94)	4.67E-09	0.89 (0.86, 0.92)	6.04E-12	0.85 (0.74, 0.92)	9.59E-04	Yes	Yes		
rs7187	19	11275258	G	A	1.10 (1.07, 1.13)	9.17E-10	1.12 (1.08, 1.15)	9.60E-12	1.00 (0.92, 1.15)	6.79E-01	Yes	No	G,	KANK2
rs395908	19	45373565	A	G	0.89 (0.86, 0.93)	4.74E-09	0.87 (0.83, 0.90)	1.16E-11	0.82 (0.68, 0.92)	2.30E-03	Yes	Yes	G,	PVRL2
rs2954025	8	126484463	C	T	1.11 (1.08, 1.14)	1.28E-12	1.11 (1.08, 1.15)	2.32E-11	1.10 (1.00, 1.25)	5.26E-02	Yes	No		
rs4336630	8	126484276	T	G	1.11 (1.08, 1.14)	1.57E-12	1.11 (1.08, 1.15)	3.61E-11	1.10 (0.99, 1.25)	6.19E-02	Yes	No		
rs6859	19	45382034	A	G	1.09 (1.06, 1.12)	1.57E-08	1.11 (1.07, 1.14)	1.38E-10	1.05 (0.97, 1.20)	1.70E-01	Yes	No	G,	PVRL2

Table S-17. All genome-wide significant marginal associations, dyslipidaemia (continued).

RSID	Chr	Position	A1	A0	Discovery, unadjusted		Discovery, adjusted		Replication, adjusted		Genome-wide sig.?	Replicated?	Annotation	Gene
					OR (95% CI)	P	OR (95% CI)	P	OR (95% CI)	P				
rs11096689	2	21140540	C	T	1.09 (1.06, 1.13)	9.79E-08	1.12 (1.08, 1.15)	1.89E-10	1.10 (0.99, 1.26)	7.09E-02	Yes	No		
rs780093	2	27742603	T	C	1.10 (1.07, 1.13)	1.81E-10	1.10 (1.07, 1.14)	2.14E-10	1.00 (0.88, 1.09)	7.40E-01	Yes	No	D, G,	GCKR
rs11685356	2	21197159	T	C	1.10 (1.07, 1.14)	3.86E-09	1.12 (1.08, 1.16)	2.34E-10	1.13 (1.00, 1.27)	5.63E-02	Yes	No		
rs651007	9	136153875	T	C	1.12 (1.08, 1.15)	6.52E-10	1.13 (1.08, 1.17)	2.46E-10	1.10 (0.98, 1.27)	8.83E-02	Yes	No	G,	ABO
rs12654264	5	74648603	T	A	1.09 (1.06, 1.12)	4.07E-09	1.10 (1.07, 1.14)	3.01E-10	1.15 (1.04, 1.29)	9.05E-03	Yes	Yes	D, G,	HMGCR
rs35239705	2	21451687	A	G	0.91 (0.88, 0.94)	8.41E-08	0.89 (0.86, 0.92)	4.05E-10	0.89 (0.78, 1.02)	1.02E-01	Yes	No		
rs11749783	5	74626082	C	T	1.09 (1.06, 1.12)	3.21E-09	1.10 (1.07, 1.14)	4.28E-10	1.13 (1.02, 1.26)	2.38E-02	Yes	Yes		
rs780094	2	27741237	T	C	1.10 (1.07, 1.13)	2.05E-10	1.10 (1.07, 1.14)	4.36E-10	1.00 (0.89, 1.11)	8.72E-01	Yes	No	R, D, G,	GCKR
rs7703051	5	74625487	A	C	1.09 (1.06, 1.12)	5.59E-09	1.10 (1.07, 1.14)	7.20E-10	1.13 (1.02, 1.27)	2.28E-02	Yes	Yes		
rs5744680	5	74879890	A	G	1.09 (1.06, 1.12)	3.00E-09	1.10 (1.07, 1.13)	8.02E-10	1.09 (0.97, 1.21)	1.37E-01	Yes	No	G,	POLK
rs10207315	2	21196346	T	G	1.10 (1.06, 1.13)	3.43E-08	1.11 (1.08, 1.15)	1.02E-09	1.14 (1.00, 1.28)	5.03E-02	Yes	No		
rs3846663	5	74655726	T	C	1.09 (1.06, 1.12)	7.58E-09	1.10 (1.07, 1.13)	1.05E-09	1.14 (1.04, 1.29)	9.72E-03	Yes	Yes	D, G,	HMGCR
rs4704220	5	74757556	A	G	1.09 (1.06, 1.12)	4.45E-09	1.10 (1.07, 1.13)	1.24E-09	1.09 (0.97, 1.21)	1.35E-01	Yes	No	G,	COL4A3BP
rs4704223	5	74773420	C	T	1.09 (1.06, 1.12)	3.65E-09	1.10 (1.07, 1.13)	1.33E-09	1.10 (0.98, 1.22)	1.03E-01	Yes	No	G,	COL4A3BP
rs4704219	5	74748505	T	C	1.09 (1.06, 1.12)	4.98E-09	1.10 (1.07, 1.13)	1.41E-09	NA (0.97, 1.20)	1.74E-01	Yes	No	G,	COL4A3BP
rs3764261	16	56993324	A	C	0.91 (0.88, 0.94)	1.56E-09	0.91 (0.88, 0.94)	1.42E-09	1.05 (0.92, 1.16)	5.66E-01	Yes	No	D, G,	CETP
rs2335418	5	74603479	A	G	1.09 (1.06, 1.12)	2.72E-09	1.10 (1.06, 1.13)	1.44E-09	1.10 (0.99, 1.23)	6.39E-02	Yes	No		
rs4549504	5	74808237	T	G	1.09 (1.06, 1.12)	7.16E-09	1.10 (1.07, 1.13)	1.50E-09	NA (0.98, 1.22)	9.91E-02	Yes	No	G,	POLK
rs473770	2	21403965	G	A	0.91 (0.88, 0.94)	2.59E-08	0.90 (0.87, 0.93)	1.83E-09	0.85 (0.74, 0.95)	4.66E-03	Yes	Yes		
rs12149545	16	56993161	A	G	0.91 (0.88, 0.94)	2.30E-09	0.91 (0.88, 0.94)	1.95E-09	1.04 (0.92, 1.16)	6.30E-01	Yes	No	D, G,	CETP
rs11748027	5	74909972	T	C	1.09 (1.06, 1.12)	6.80E-09	1.10 (1.06, 1.13)	2.17E-09	1.09 (0.97, 1.21)	1.45E-01	Yes	No	G,	ANKDD1B
rs4704221	5	74759183	A	T	1.09 (1.06, 1.12)	1.14E-08	1.10 (1.06, 1.13)	2.25E-09	1.10 (0.99, 1.22)	9.10E-02	Yes	No	G,	COL4A3BP
rs6544713	2	44073881	T	C	1.09 (1.06, 1.12)	1.46E-08	1.10 (1.07, 1.14)	2.58E-09	1.02 (0.91, 1.14)	7.19E-01	Yes	No	D, G,	ABCG8
rs1551894	5	74570531	A	G	1.11 (1.07, 1.15)	3.04E-09	1.12 (1.08, 1.16)	2.71E-09	1.10 (0.96, 1.24)	1.76E-01	Yes	No	R,	
rs6878990	5	74689249	C	T	1.09 (1.06, 1.12)	1.02E-08	1.10 (1.06, 1.13)	2.71E-09	1.09 (0.97, 1.21)	1.36E-01	Yes	No	G,	COL4A3BP
rs11957260	5	74608732	T	C	1.09 (1.06, 1.12)	6.01E-09	1.10 (1.06, 1.13)	2.95E-09	1.10 (0.99, 1.23)	7.19E-02	Yes	No		
rs17671591	5	74615021	T	C	1.10 (1.06, 1.13)	1.49E-09	1.10 (1.07, 1.13)	3.28E-09	1.17 (1.05, 1.31)	4.71E-03	Yes	Yes	R,	
rs4604177	5	74808880	T	C	1.09 (1.06, 1.12)	1.74E-08	1.10 (1.06, 1.13)	4.20E-09	1.09 (0.98, 1.21)	1.16E-01	Yes	No	R, G,	POLK
rs2000999	16	72108093	A	G	1.12 (1.08, 1.16)	4.15E-10	1.12 (1.08, 1.16)	6.86E-09	NA (1.01, 1.32)	3.45E-02	Yes	Yes	G,	HPR
rs3828793	6	32635883	T	C	1.11 (1.07, 1.15)	7.89E-08	1.13 (1.08, 1.17)	7.23E-09	1.10 (0.95, 1.27)	2.21E-01	Yes	No	R, G,	HLA-DQB1
rs1728918	2	27635463	A	G	1.09 (1.06, 1.13)	2.32E-08	1.10 (1.07, 1.14)	9.42E-09	0.99 (0.86, 1.09)	5.78E-01	Yes	No	G,	PPM1G
rs76186695	19	10908623	T	G	0.92 (0.88, 0.95)	1.54E-06	0.89 (0.86, 0.93)	1.23E-08	0.97 (0.80, 1.05)	1.96E-01	Yes	No	R, G,	DNM2
rs4704200	5	74569856	T	G	1.09 (1.06, 1.12)	1.10E-08	1.09 (1.06, 1.12)	1.64E-08	1.06 (0.97, 1.20)	1.67E-01	Yes	No		
rs2304087	19	11107133	C	T	0.93 (0.90, 0.96)	6.53E-07	0.91 (0.89, 0.94)	1.69E-08	0.90 (0.77, 0.97)	1.10E-02	Yes	Yes	G,	SMARCA4
rs7254776	19	45227742	C	T	0.93 (0.91, 0.96)	2.93E-06	0.91 (0.89, 0.94)	1.73E-08	0.94 (0.83, 1.04)	2.12E-01	Yes	No		
rs2980885	8	126474306	G	A	1.11 (1.07, 1.14)	2.91E-09	1.11 (1.07, 1.15)	1.80E-08	1.07 (0.95, 1.22)	2.51E-01	Yes	No		
rs1794275	6	32671248	A	G	1.11 (1.07, 1.15)	4.06E-08	1.12 (1.08, 1.17)	1.96E-08	1.08 (0.94, 1.25)	2.71E-01	Yes	No		
rs1423527	5	74602699	A	C	1.08 (1.05, 1.11)	6.41E-08	1.09 (1.06, 1.12)	2.28E-08	1.12 (1.01, 1.26)	2.66E-02	Yes	Yes		
rs28396311	6	32628190	C	T	1.10 (1.06, 1.15)	2.55E-07	1.12 (1.08, 1.17)	2.54E-08	1.08 (0.95, 1.26)	2.29E-01	Yes	No	G,	HLA-DQB1
rs6871667	5	74604742	A	G	1.08 (1.05, 1.11)	1.73E-07	1.09 (1.06, 1.12)	3.55E-08	1.14 (1.01, 1.25)	3.57E-02	Yes	Yes		
rs588245	2	21270057	A	G	1.08 (1.05, 1.11)	1.99E-07	1.09 (1.06, 1.12)	3.98E-08	0.95 (0.85, 1.05)	2.65E-01	Yes	No	D, G,	APOB
rs7730344	5	74587940	A	C	1.09 (1.05, 1.12)	1.31E-07	1.10 (1.06, 1.13)	4.45E-08	1.00 (0.89, 1.12)	9.57E-01	Yes	No		
rs13207836	6	32596374	G	A	1.10 (1.06, 1.15)	7.71E-07	1.12 (1.07, 1.17)	6.08E-08	1.06 (0.94, 1.26)	2.73E-01	Yes	No		
rs34947241	6	32592239	C	T	1.11 (1.06, 1.15)	4.60E-07	1.12 (1.07, 1.17)	7.68E-08	1.07 (0.95, 1.27)	2.18E-01	Yes	No		

Table S-17. All genome-wide significant marginal associations, dyslipidaemia (continued).

RSID	Chr	Position	A1	A0	Discovery, unadjusted		Discovery, adjusted		Replication, adjusted		Genome-wide sig.?	Replicated?	Annotation	Gene
					OR (95% CI)	P	OR (95% CI)	P	OR (95% CI)	P				
rs4248166	6	32366421	C	T	1.09 (1.05, 1.14)	4.70E-06	1.12 (1.07, 1.16)	8.57E-08	0.97 (0.83, 1.12)	6.32E-01	Yes	No	R, G,	BTNL2
rs35014460	6	32591106	C	T	1.10 (1.06, 1.14)	1.05E-06	1.12 (1.07, 1.16)	8.63E-08	1.04 (0.92, 1.24)	3.78E-01	Yes	No		
