# Supplementary material for: Exhaustive Genome-Wide Search for SNP-SNP Interactions Across 10 Human Diseases
Source: G3 (Bethesda). 2016 May 12;6(7):2043–50. doi: 10.1534/g3.116.028563 (PMC4938657; doi:10.1534/g3.116.028563)
Supplement: Supplemental Material [file supp_g3.116.028563_TableS18.pdf]

Table S-18. Top 10 most significant marginal associations, hemorrhoids.

RSID	Chr	Position	A1	A0	Discovery, unadjusted		Discovery, adjusted		Replication, adjusted		Genome-wide sig.?	Replicated?	Annotation	Gene
					OR (95% CI)	P	OR (95% CI)	P	OR (95% CI)	P				
rs6106205	20	20158867	C	T	1.11 (1.07, 1.16)	5.52E-07	1.11 (1.07, 1.16)	6.57E-07	1.01 (1.00, 1.00)	9.99E-01	No	No	G,	C20orf26
rs1350852	18	39905989	A	G	1.11 (1.07, 1.16)	7.87E-07	1.11 (1.07, 1.16)	7.80E-07	1.01 (0.91, 1.14)	7.97E-01	No	No	G,	LINC00907
rs952793	20	6970831	T	C	1.14 (1.08, 1.19)	1.38E-06	1.14 (1.08, 1.20)	1.18E-06	1.06 (0.93, 1.22)	3.63E-01	No	No		
rs2431718	5	148853121	A	G	1.10 (1.06, 1.14)	3.12E-06	1.10 (1.05, 1.14)	4.77E-06	1.02 (0.91, 1.13)	7.80E-01	No	No		
rs2633815	3	156573214	G	A	1.12 (1.06, 1.17)	9.35E-06	1.12 (1.06, 1.17)	1.07E-05	0.98 (0.86, 1.12)	7.60E-01	No	No	G,	LEKR1
rs414683	3	156570703	G	A	1.11 (1.06, 1.17)	1.24E-05	1.11 (1.06, 1.17)	1.41E-05	0.98 (0.85, 1.11)	7.17E-01	No	No	G,	RNF141
rs6806773	3	195571024	A	G	0.90 (0.85, 0.94)	6.44E-06	0.90 (0.86, 0.94)	1.49E-05	0.98 (0.86, 1.10)	6.55E-01	No	No		
rs1426802	12	74737650	T	C	0.91 (0.87, 0.95)	2.44E-05	0.91 (0.87, 0.95)	1.63E-05	1.03 (0.93, 1.16)	4.97E-01	No	No		
rs3865420	18	56010715	C	T	0.90 (0.85, 0.94)	9.06E-06	0.90 (0.85, 0.94)	1.72E-05	1.07 (0.95, 1.21)	2.80E-01	No	No	G,	NEDD4L
rs7776573	7	31438342	C	A	1.10 (1.05, 1.15)	3.07E-05	1.10 (1.05, 1.15)	1.81E-05	0.97 (0.86, 1.10)	6.94E-01	No	No		
