# Supplementary material for: Exhaustive Genome-Wide Search for SNP-SNP Interactions Across 10 Human Diseases
Source: G3 (Bethesda). 2016 May 12;6(7):2043–50. doi: 10.1534/g3.116.028563 (PMC4938657; doi:10.1534/g3.116.028563)
Supplement: Supplemental Material [file supp_g3.116.028563_TableS19.pdf]

**Table S-19. Top 10 most significant marginal associations, hypertensive disease.**

| RSID | Chr | Position | A1 | A0 | Discovery, unadjusted | | Discovery, adjusted | | Replication, adjusted | | Genome-wide sig.? | Replicated? | Annotation | Gene |
|---|---|---|---|---|---|---|---|---|---|---|---|---|---|---|
| | | | | | OR (95% CI) | P | OR (95% CI) | P | OR (95% CI) | P | | | | |
| rs1275985 | 2 | 26911745 | C | T | 1.09 (1.06, 1.12) | 1.02E-08 | 1.11 (1.07, 1.14) | 2.82E-10 | 1.17 (1.07, 1.34) | 1.84E-03 | Yes | Yes | G, | KCNK3 |
| rs1275988 | 2 | 26914364 | C | T | 1.08 (1.05, 1.12) | 3.69E-08 | 1.11 (1.07, 1.14) | 4.45E-10 | 1.16 (1.06, 1.32) | 3.53E-03 | Yes | Yes | G, | KCNK3 |
| rs9322334 | 6 | 152200041 | A | G | 1.11 (1.07, 1.15) | 1.65E-09 | 1.11 (1.07, 1.15) | 6.62E-08 | 0.96 (0.83, 1.08) | 4.56E-01 | Yes | No | D, G, | ESR1 |
| rs1458038 | 4 | 81164723 | T | C | 1.10 (1.06, 1.13) | 7.22E-09 | 1.10 (1.06, 1.14) | 7.77E-08 | 1.04 (0.92, 1.18) | 5.19E-01 | Yes | No | | |
| rs9322335 | 6 | 152200129 | T | C | 1.10 (1.06, 1.14) | 1.26E-08 | 1.10 (1.06, 1.14) | 2.87E-07 | 0.99 (0.87, 1.13) | 8.84E-01 | No | No | D, G, | ESR1 |
| rs7112413 | 11 | 10265500 | T | C | 1.08 (1.04, 1.12) | 6.38E-05 | 1.11 (1.06, 1.15) | 8.32E-07 | 0.91 (0.80, 1.07) | 2.76E-01 | No | No | G, | SBF2 |
| rs2627321 | 15 | 81022569 | T | C | 1.07 (1.04, 1.10) | 1.36E-06 | 1.08 (1.05, 1.11) | 1.84E-06 | 1.01 (0.93, 1.16) | 4.93E-01 | No | No | G, | ABHD17C |
| rs4845953 | 1 | 10801989 | G | A | 1.06 (1.03, 1.09) | 2.33E-05 | 1.08 (1.05, 1.11) | 2.07E-06 | 1.00 (0.91, 1.14) | 7.28E-01 | No | No | R, D, G, | CASZ1 |
| rs7173751 | 15 | 80998903 | A | G | 1.07 (1.04, 1.10) | 1.47E-06 | 1.08 (1.04, 1.11) | 2.81E-06 | 1.04 (0.94, 1.18) | 3.78E-01 | No | No | G, | ABHD17C |
| rs2596126 | 8 | 76640621 | G | A | 0.94 (0.91, 0.97) | 2.08E-05 | 0.93 (0.90, 0.96) | 3.18E-06 | 1.07 (0.96, 1.21) | 1.81E-01 | No | No | | |
