# Supplementary material for: Exhaustive Genome-Wide Search for SNP-SNP Interactions Across 10 Human Diseases
Source: G3 (Bethesda). 2016 May 12;6(7):2043–50. doi: 10.1534/g3.116.028563 (PMC4938657; doi:10.1534/g3.116.028563)
Supplement: Supplemental Material [file supp_g3.116.028563_TableS20.pdf]

Table S-20. Top 10 most significant marginal associations, osteoarthritis.

RSID	Chr	Position	A1	A0	Discovery, unadjusted		Discovery, adjusted		Replication, adjusted		Genome-wide sig.?	Replicated?	Annotation	Gene
					OR (95% CI)	P	OR (95% CI)	P	OR (95% CI)	P				
rs6925021	6	156588729	A	G	0.94 (0.91, 0.97)	2.96E-04	0.92 (0.89, 0.95)	1.92E-06	1.05 (0.94, 1.19)	3.62E-01	No	No		
rs2083418	2	42335185	T	C	0.94 (0.92, 0.97)	6.36E-05	0.93 (0.90, 0.96)	3.70E-06	1.09 (0.96, 1.20)	2.13E-01	No	No	R,	
rs1017476	2	42324553	C	A	0.94 (0.91, 0.97)	4.10E-05	0.93 (0.90, 0.96)	4.47E-06	1.10 (0.98, 1.23)	1.16E-01	No	No		
rs36039266	3	14303604	C	T	0.93 (0.90, 0.95)	1.27E-06	0.93 (0.89, 0.96)	7.24E-06	0.94 (0.84, 1.07)	3.65E-01	No	No		
rs828152	4	17063564	T	C	0.93 (0.90, 0.97)	9.28E-04	0.91 (0.87, 0.95)	1.91E-05	1.05 (0.87, 1.18)	8.70E-01	No	No		
rs11508026	16	56999328	T	C	1.06 (1.03, 1.09)	7.74E-05	1.07 (1.04, 1.10)	2.10E-05	1.01 (0.91, 1.12)	8.54E-01	No	No	G,	CETP
rs9514592	13	107558936	T	C	0.94 (0.91, 0.97)	1.54E-04	0.93 (0.90, 0.96)	2.83E-05	1.03 (0.93, 1.18)	4.33E-01	No	No		
rs77242996	3	73683549	G	A	0.94 (0.91, 0.97)	1.55E-05	0.94 (0.91, 0.97)	3.01E-05	1.01 (0.89, 1.11)	8.75E-01	No	No		
rs9404236	6	103186025	A	G	1.09 (1.05, 1.13)	1.05E-05	1.09 (1.05, 1.13)	3.41E-05	0.96 (0.85, 1.14)	8.28E-01	No	No		
rs12407873	1	167796012	C	A	1.06 (1.03, 1.09)	1.24E-04	1.07 (1.03, 1.10)	4.11E-05	1.06 (0.95, 1.19)	2.83E-01	No	No	G,	ADCY10
