# Supplementary material for: Exhaustive Genome-Wide Search for SNP-SNP Interactions Across 10 Human Diseases
Source: G3 (Bethesda). 2016 May 12;6(7):2043–50. doi: 10.1534/g3.116.028563 (PMC4938657; doi:10.1534/g3.116.028563)
Supplement: Supplemental Material [file supp_g3.116.028563_TableS21.pdf]

Table S-21. Enrichment analysis (allergic rhinitis).

Annotation	Match group	FastEpistasis / logistic regression					BOOST				
		N, total	N, rep. (%)	Enrich P	Min Int P	Thresh	N, total	N, rep.	Enrich P	Min Int P	Thresh
Exonic	None	3367	2.4	0.627	A: 8.61E-11<br>R: 2.08E-09	1.13E-12	3790	5.2	0.014	A: 3.92E-11<br>R: 1.14E-09	1.13E-12
	Either	46	0		A: 2.85E-09<br>R: N/A	6.39E-11	61	13.1		A: 1.78E-10<br>R: 8.34E-09	6.39E-11
	Both	0	0		A: N/A<br>R: N/A	1.45E-08	0	0		A: N/A<br>R: N/A	1.45E-08
Reg. 1,2, or 3	None	3151	2.4	0.700	A: 8.61E-11<br>R: 2.08E-09	1.21E-12	3559	5.1	0.215	A: 3.92E-11<br>R: 1.14E-09	1.21E-12
	Either	258	2.7		A: 8.02E-10<br>R: 2.20E-08	1.42E-11	289	7.6		A: 4.88E-10<br>R: 5.07E-09	1.42E-11
	Both	4	0		A: 5.47E-09<br>R: N/A	6.73E-10	3	0		A: 4.48E-08<br>R: N/A	6.73E-10
Disease EQTL	None	3406	2.4	1.000	A: 8.61E-11<br>R: 2.08E-09	1.12E-12	3836	5.3	1.000	A: 3.92E-11<br>R: 1.14E-09	1.12E-12
	Either	7	0		A: 1.77E-08<br>R: N/A	1.96E-10	15	0		A: 3.14E-09<br>R: N/A	1.96E-10
	Both	0	0		A: N/A<br>R: N/A	1.38E-07	0	0		A: N/A<br>R: N/A	1.38E-07
Disease gene	None	3401	2.4	1.000	A: 8.61E-11<br>R: 2.08E-09	1.12E-12	3840	5.3	0.112	A: 3.92E-11<br>R: 1.14E-09	1.12E-12
	Either	12	0		A: 8.22E-09<br>R: N/A	1.96E-10	11	18.2		A: 3.03E-08<br>R: 5.74E-08	1.96E-10
	Both	0	0		A: N/A<br>R: N/A	1.38E-07	0	0		A: N/A<br>R: N/A	1.38E-07
Any gene	None	1060	2.3	0.266	A: 2.17E-10<br>R: 2.15E-09	3.75E-12	1186	4.5	0.278	A: 2.73E-10<br>R: 1.86E-09	3.75E-12
	Either	1678	2.7		A: 8.61E-11<br>R: 2.08E-09	2.24E-12	1896	5.5		A: 7.64E-11<br>R: 1.48E-09	2.24E-12
	Both	675	1.6		A: 2.38E-10<br>R: 1.97E-08	5.34E-12	769	6.0		A: 3.92E-11<br>R: 1.14E-09	5.34E-12
Marginal	None	3047	2.5	0.240	A: 8.61E-11<br>R: 2.08E-09	1.24E-12	3417	5.1	0.152	A: 3.92E-11<br>R: 1.14E-09	1.24E-12
	Either	352	1.4		A: 9.04E-11<br>R: 2.63E-08	1.08E-11	415	7.0		A: 4.53E-10<br>R: 1.87E-09	1.08E-11
	Both	14	7.1		A: 8.36E-09<br>R: 8.28E-08	3.78E-10	19	10.5		A: 3.23E-08<br>R: 4.48E-08	3.78E-10

Followed-up interactions (interactions with $P<10^{-7}$ in either the FastEpistasis or BOOST analyses) were grouped by the number of SNPs that match a particular annotation category (**Match group**: none, either, or both SNPs matching). Annotation categories are described in the Supplemental Methods section. **N, total**: total number of interactions in each annotation match group. **N, rep (%)**: percentage of the total that were nominally replicated. **Enrich P**: enrichment test P-value (testing if there is a significant difference between percentages of nominally replicated interactions among the different annotation match groups; chi-square test or Fisher's exact test). **Min Int P**: among all interactions in the particular annotation match category, the P-value of the most significant interaction (A), and the P-value of the most significant nominally replicated interaction (R). **Thresh**: hypothetical significance threshold assuming that the search space was first trimmed to only include SNP pairs corresponding to the particular annotation match group. Green shading: enrichment P-value < 0.05. Cyan shading: a "Min Int P" value that is less than the respective "Thresh".
