# Supplementary material for: Exhaustive Genome-Wide Search for SNP-SNP Interactions Across 10 Human Diseases
Source: G3 (Bethesda). 2016 May 12;6(7):2043–50. doi: 10.1534/g3.116.028563 (PMC4938657; doi:10.1534/g3.116.028563)
Supplement: Supplemental Material [file supp_g3.116.028563_TableS22.pdf]

Table S-22. Enrichment analysis (asthma).

Annotation	Match group	FastEpistasis / logistic regression					BOOST				
		N, total	N, rep. (%)	Enrich P	Min Int P	Thresh	N, total	N, rep.	Enrich P	Min Int P	Thresh
Exonic	None	3303	2.2	0.626	A: 2.50E-11<br>R: 3.53E-10	1.13E-12	3913	4.9	0.100	A: 2.55E-11<br>R: 8.78E-10	1.13E-12
	Either	45	0		A: 3.90E-09<br>R: N/A	6.38E-11	54	11.1		A: 4.19E-09<br>R: 1.90E-08	6.38E-11
	Both	0	0		A: N/A<br>R: N/A	1.45E-08	1	0		A: 6.77E-08<br>R: N/A	1.45E-08
Reg. 1,2, or 3	None	3109	2.1	0.366	A: 2.50E-11<br>R: 3.53E-10	1.20E-12	3669	4.8	0.152	A: 2.55E-11<br>R: 8.78E-10	1.20E-12
	Either	238	2.9		A: 2.52E-10<br>R: 1.49E-09	1.42E-11	291	7.2		A: 5.19E-11<br>R: 6.90E-09	1.42E-11
	Both	1	0		A: 1.94E-08<br>R: N/A	6.76E-10	8	0		A: 9.61E-10<br>R: N/A	6.76E-10
Disease EQTL	None	3327	2.1	0.074	A: 2.50E-11<br>R: 3.53E-10	1.12E-12	3943	5.0	0.648	A: 2.55E-11<br>R: 8.78E-10	1.12E-12
	Either	21	9.5		A: 1.84E-09<br>R: 1.77E-08	9.16E-11	24	0		A: 6.01E-10<br>R: N/A	9.16E-11
	Both	0	0		A: N/A<br>R: N/A	3.00E-08	1	0		A: 9.14E-08<br>R: N/A	3.00E-08
Disease gene	None	3068	2.1	0.227	A: 2.50E-11<br>R: 3.53E-10	1.21E-12	3634	5.1	0.313	A: 2.55E-11<br>R: 8.78E-10	1.21E-12
	Either	278	3.2		A: 6.27E-11<br>R: 1.29E-09	1.35E-11	327	3.4		A: 5.19E-11<br>R: 3.64E-09	1.35E-11
	Both	2	0		A: 5.02E-10<br>R: N/A	6.06E-10	7	0		A: 2.77E-09<br>R: N/A	6.06E-10
Any gene	None	1057	2.0	0.831	A: 2.50E-11<br>R: 1.27E-08	3.72E-12	1202	5.2	0.351	A: 1.28E-10<br>R: 2.87E-09	3.72E-12
	Either	1631	2.2		A: 8.34E-11<br>R: 3.53E-10	2.23E-12	1983	5.2		A: 2.55E-11<br>R: 1.00E-09	2.23E-12
	Both	660	2.4		A: 6.27E-11<br>R: 1.86E-09	5.36E-12	783	4.0		A: 3.02E-10<br>R: 8.78E-10	5.36E-12
Marginal	None	2978	2.2	0.069	A: 2.50E-11<br>R: 3.53E-10	1.24E-12	3568	4.9	0.753	A: 2.55E-11<br>R: 8.78E-10	1.24E-12
	Either	363	1.7		A: 1.89E-10<br>R: 5.42E-08	1.04E-11	390	5.1		A: 2.52E-10<br>R: 1.90E-08	1.04E-11
	Both	7	14.3		A: 1.03E-08<br>R: 1.03E-08	3.47E-10	10	10.0		A: 5.67E-09<br>R: 6.51E-09	3.47E-10

Followed-up interactions (interactions with $P<10^{-7}$ in either the FastEpistasis or BOOST analyses) were grouped by the number of SNPs that match a particular annotation category (**Match group**: none, either, or both SNPs matching). Annotation categories are described in the Supplemental Methods section. **N, total**: total number of interactions in each annotation match group. **N, rep (%)**: percentage of the total that were nominally replicated. **Enrich P**: enrichment test P-value (testing if there is a significant difference between percentages of nominally replicated interactions among the different annotation match groups; chi-square test or Fisher's exact test). **Min Int P**: among all interactions in the particular annotation match category, the P-value of the most significant interaction (A), and the P-value of the most significant nominally replicated interaction (R). **Thresh**: hypothetical significance threshold assuming that the search space was first trimmed to only include SNP pairs corresponding to the particular annotation match group. Green shading: enrichment P-value < 0.05. Cyan shading: a "Min Int P" value that is less than the respective "Thresh".
