# Supplementary material for: Exhaustive Genome-Wide Search for SNP-SNP Interactions Across 10 Human Diseases
Source: G3 (Bethesda). 2016 May 12;6(7):2043–50. doi: 10.1534/g3.116.028563 (PMC4938657; doi:10.1534/g3.116.028563)
Supplement: Supplemental Material [file supp_g3.116.028563_TableS23.pdf]

Table S-23. Enrichment analysis (cardiac disease).

Annotation	Match group	FastEpistasis / logistic regression					BOOST				
		N, total	N, rep. (%)	Enrich P	Min Int P	Thresh	N, total	N, rep.	Enrich P	Min Int P	Thresh
Exonic	None	3421	2.6		A: 1.64E-11<br>R: 4.19E-09	1.12E-12	3919	5.5		A: 6.54E-11<br>R: 4.51E-10	1.12E-12
	Either	33	0	1.000	A: 9.75E-11<br>R: N/A	6.38E-11	47	4.3	1.000	A: 2.35E-09<br>R: 3.63E-08	6.38E-11
	Both	0	0		A: N/A<br>R: N/A	1.45E-08	0	0		A: N/A<br>R: N/A	1.45E-08
Reg. 1,2, or 3	None	3209	2.6		A: 1.64E-11<br>R: 4.19E-09	1.20E-12	3656	5.5		A: 6.54E-11<br>R: 4.51E-10	1.20E-12
	Either	240	2.1	0.852	A: 2.12E-11<br>R: 7.65E-08	1.42E-11	308	5.2	1.000	A: 1.82E-10<br>R: 7.78E-09	1.42E-11
	Both	5	0		A: 4.56E-09<br>R: N/A	6.73E-10	2	0		A: 2.40E-08<br>R: N/A	6.73E-10
Disease EQTL	None	3425	2.5		A: 1.64E-11<br>R: 4.19E-09	1.12E-12	3926	5.5		A: 6.54E-11<br>R: 4.51E-10	1.12E-12
	Either	29	3.5	0.528	A: 3.67E-09<br>R: 2.40E-07	7.16E-11	40	7.5	0.480	A: 1.62E-09<br>R: 6.67E-09	7.16E-11
	Both	0	0		A: N/A<br>R: N/A	1.83E-08	0	0		A: N/A<br>R: N/A	1.83E-08
Disease gene	None	2837	2.8		A: 1.64E-11<br>R: 4.19E-09	1.35E-12	3229	5.6		A: 6.54E-11<br>R: 4.51E-10	1.35E-12
	Either	586	1.4	0.084	A: 2.19E-11<br>R: 1.81E-07	6.37E-12	691	4.9	0.462	A: 8.54E-11<br>R: 3.37E-09	6.37E-12
	Both	31	0		A: 9.72E-09<br>R: N/A	1.20E-10	46	2.2		A: 2.75E-09<br>R: 7.20E-08	1.20E-10
Any gene	None	1088	3.4		A: 1.64E-11<br>R: 1.36E-08	3.72E-12	1185	6.2		A: 1.16E-10<br>R: 4.51E-10	3.72E-12
	Either	1668	2.3	0.067	A: 2.12E-11<br>R: 4.19E-09	2.23E-12	1973	5.1	0.426	A: 6.54E-11<br>R: 1.25E-09	2.23E-12
	Both	698	1.7		A: 9.75E-11<br>R: 4.97E-09	5.34E-12	808	5.5		A: 8.54E-11<br>R: 2.15E-09	5.34E-12
Marginal	None	3086	2.5		A: 1.64E-11<br>R: 4.19E-09	1.24E-12	3543	5.2		A: 6.54E-11<br>R: 4.51E-10	1.24E-12
	Either	353	2.0	8.03E-05	A: 3.18E-10<br>R: 3.94E-08	1.08E-11	415	7.0	1.15E-04	A: 3.37E-10<br>R: 2.15E-09	1.08E-11
	Both	15	20.0 *		A: 1.29E-08<br>R: 1.09E-07	3.77E-10	8	37.5		A: 1.23E-08<br>R: 1.23E-08	3.77E-10

Followed-up interactions (interactions with $P<10^{-7}$ in either the FastEpistasis or BOOST analyses) were grouped by the number of SNPs that match a particular annotation category (**Match group**: none, either, or both SNPs matching). Annotation categories are described in the Supplemental Methods section. **N, total**: total number of interactions in each annotation match group. **N, rep (%)**: percentage of the total that were nominally replicated. **Enrich P**: enrichment test P-value (testing if there is a significant difference between percentages of nominally replicated interactions among the different annotation match groups; chi-square test or Fisher's exact test). **Min Int P**: among all interactions in the particular annotation match category, the P-value of the most significant interaction (A), and the P-value of the most significant nominally replicated interaction (R). **Thresh**: hypothetical significance threshold assuming that the search space was first trimmed to only include SNP pairs corresponding to the particular annotation match group. Green shading: enrichment P-value < 0.05. Cyan shading: a "Min Int P" value that is less than the respective "Thresh".

* The three interactions that were nominally replicated (among FastEpistasis-identified interactions where both SNPs were marginally significant; i.e., 20.0% of 15 in the table above) were the following: rs17018095 × rs2206426, rs10485227 × rs61352759, rs11768588 × rs11105883.
