# Supplementary material for: Exhaustive Genome-Wide Search for SNP-SNP Interactions Across 10 Human Diseases
Source: G3 (Bethesda). 2016 May 12;6(7):2043–50. doi: 10.1534/g3.116.028563 (PMC4938657; doi:10.1534/g3.116.028563)
Supplement: Supplemental Material [file supp_g3.116.028563_TableS24.pdf]

Table S-24. Enrichment analysis (depression).

Annotation	Match group	FastEpistasis / logistic regression					BOOST				
		N, total	N, rep. (%)	Enrich P	Min Int P	Thresh	N, total	N, rep.	Enrich P	Min Int P	Thresh
Exonic	None	3435	2.5	0.188	A: 1.81E-11<br>R: 1.26E-10	1.13E-12	3838	5.3	0.6000	A: 4.85E-11<br>R: 6.16E-10	1.13E-12
	Either	32	6.3		A: 4.57E-10<br>R: 4.11E-08	6.40E-11	59	6.8		A: 1.31E-09<br>R: 1.40E-08	6.40E-11
	Both	0	0		A: N/A<br>R: N/A	1.45E-08	2	0		A: 7.60E-08<br>R: N/A	1.45E-08
Reg. 1,2, or 3	None	3209	2.4	0.561	A: 1.81E-11<br>R: 1.26E-10	1.21E-12	3607	5.4	0.574	A: 4.85E-11<br>R: 6.16E-10	1.21E-12
	Either	255	3.1		A: 6.88E-10<br>R: 1.18E-09	1.43E-11	286	4.2		A: 5.34E-11<br>R: 5.34E-09	1.43E-11
	Both	3	0		A: 9.33E-09<br>R: N/A	6.75E-10	6	0		A: 1.64E-08<br>R: N/A	6.75E-10
Disease EQTL	None	3461	2.5	1.000	A: 1.81E-11<br>R: 1.26E-10	1.11E-12	3894	5.3	1.000	A: 4.85E-11<br>R: 6.16E-10	1.11E-12
	Either	6	0		A: 9.54E-09<br>R: N/A	5.50E-10	5	0		A: 1.74E-08<br>R: N/A	5.50E-10
	Both	0	0		A: N/A<br>R: N/A	1.09E-06	0	0		A: N/A<br>R: N/A	1.09E-06
Disease gene	None	3341	2.5	0.374	A: 1.81E-11<br>R: 1.26E-10	1.16E-12	3742	5.4	0.315	A: 4.85E-11<br>R: 6.16E-10	1.16E-12
	Either	126	0.8		A: 5.14E-10<br>R: 1.38E-07	2.68E-11	156	3.2		A: 1.20E-09<br>R: 4.67E-09	2.68E-11
	Both	0	0		A: N/A<br>R: N/A	2.48E-09	1	0		A: 7.88E-08<br>R: N/A	2.48E-09
Any gene	None	1078	2.1	0.494	A: 1.71E-10<br>R: 2.79E-10	3.74E-12	1135	6.4	0.134	A: 1.16E-10<br>R: 3.96E-09	3.74E-12
	Either	1733	2.5		A: 1.81E-11<br>R: 1.26E-10	2.24E-12	1993	4.9		A: 4.85E-11<br>R: 6.16E-10	2.24E-12
	Both	656	3.1		A: 1.33E-10<br>R: 1.18E-09	5.36E-12	771	4.8		A: 1.90E-10<br>R: 1.36E-09	5.36E-12
Marginal	None	3135	2.6	0.468	A: 1.81E-11<br>R: 1.26E-10	1.23E-12	3541	5.3	0.638	A: 4.85E-11<br>R: 6.16E-10	1.23E-12
	Either	324	1.5		A: 1.33E-10<br>R: 3.12E-08	1.13E-11	345	5.8		A: 5.34E-11<br>R: 7.37E-09	1.13E-11
	Both	8	0		A: 1.07E-08<br>R: N/A	4.13E-10	13	0		A: 1.23E-08<br>R: N/A	4.13E-10

Followed-up interactions (interactions with $P<10^{-7}$ in either the FastEpistasis or BOOST analyses) were grouped by the number of SNPs that match a particular annotation category (**Match group**: none, either, or both SNPs matching). Annotation categories are described in the Supplemental Methods section. **N, total**: total number of interactions in each annotation match group. **N, rep (%)**: percentage of the total that were nominally replicated. **Enrich P**: enrichment test P-value (testing if there is a significant difference between percentages of nominally replicated interactions among the different annotation match groups; chi-square test or Fisher's exact test). **Min Int P**: among all interactions in the particular annotation match category, the P-value of the most significant interaction (A), and the P-value of the most significant nominally replicated interaction (R). **Thresh**: hypothetical significance threshold assuming that the search space was first trimmed to only include SNP pairs corresponding to the particular annotation match group. Green shading: enrichment P-value < 0.05. Cyan shading: a "Min Int P" value that is less than the respective "Thresh".
