# Supplementary material for: Exhaustive Genome-Wide Search for SNP-SNP Interactions Across 10 Human Diseases
Source: G3 (Bethesda). 2016 May 12;6(7):2043–50. doi: 10.1534/g3.116.028563 (PMC4938657; doi:10.1534/g3.116.028563)
Supplement: Supplemental Material [file supp_g3.116.028563_TableS25.pdf]

Table S-25. Enrichment analysis (dermatophytosis).

Annotation	Match group	FastEpistasis / logistic regression					BOOST				
		N, total	N, rep. (%)	Enrich P	Min Int P	Thresh	N, total	N, rep.	Enrich P	Min Int P	Thresh
Exonic	None	3578	2.6	0.395	A: 4.29E-13<br>R: 1.87E-09	1.13E-12	4000	4.8	0.540	A: 1.20E-12<br>R: 3.22E-10	1.13E-12
	Either	50	4.0		A: 1.06E-09<br>R: 1.06E-09	6.39E-11	50	2.0		A: 3.21E-09<br>R: 9.07E-08	6.39E-11
	Both	1	0		A: 1.59E-08<br>R: N/A	1.45E-08	1	0		A: 4.22E-09<br>R: N/A	1.45E-08
Reg. 1,2, or 3	None	3354	2.6	0.716	A: 4.29E-13<br>R: 1.87E-09	1.20E-12	3765	4.8	0.158	A: 1.20E-12<br>R: 3.22E-10	1.20E-12
	Either	272	2.9		A: 1.20E-10<br>R: 1.06E-09	1.43E-11	283	5.0		A: 4.08E-10<br>R: 1.99E-08	1.43E-11
	Both	3	0		A: 2.93E-10<br>R: N/A	6.75E-10	3	33.0		A: 6.13E-08<br>R: 9.13E-08	6.75E-10
Disease EQTL	None	3629	2.6	N/A	A: 4.29E-13<br>R: 1.06E-09	1.11E-12	4050	4.8	1.000	A: 1.20E-12<br>R: 3.22E-10	1.11E-12
	Either	0	0		A: N/A<br>R: N/A	2.78E-08	1	0		A: 1.69E-08<br>R: N/A	2.78E-08
	Both	0	0		A: N/A<br>R: N/A	3.33E-03	0	0		A: N/A<br>R: N/A	3.33E-03
Disease gene	None	3629	2.6	N/A	A: 4.29E-13<br>R: 1.06E-09	1.11E-12	4050	4.8	1.000	A: 1.20E-12<br>R: 3.22E-10	1.11E-12
	Either	0	0		A: N/A<br>R: N/A	6.40E-09	1	0		A: 1.69E-08<br>R: N/A	6.40E-09
	Both	0	0		A: N/A<br>R: N/A	1.54E-04	0	0		A: N/A<br>R: N/A	1.54E-04
Any gene	None	1112	2.3	0.650	A: 9.63E-12<br>R: 2.89E-09	3.74E-12	1235	5.5	0.355	A: 1.69E-10<br>R: 1.15E-09	3.74E-12
	Either	1804	2.8		A: 4.29E-13<br>R: 1.06E-09	2.24E-12	2004	4.5		A: 1.20E-12<br>R: 1.29E-09	2.24E-12
	Both	713	2.8		A: 1.75E-10<br>R: 1.87E-09	5.35E-12	812	4.3		A: 7.23E-11<br>R: 3.22E-10	5.35E-12
Marginal	None	3237	2.8	0.109	A: 4.29E-13<br>R: 1.06E-09	1.24E-12	3629	4.8	0.572	A: 5.20E-11<br>R: 3.22E-10	1.24E-12
	Either	384	1.0		A: 5.27E-11<br>R: 5.13E-09	1.09E-11	403	5.2		A: 1.20E-12<br>R: 1.29E-09	1.09E-11
	Both	8	0		A: 2.67E-08<br>R: N/A	3.86E-10	19	0		A: 5.13E-09<br>R: N/A	3.86E-10

Followed-up interactions (interactions with $P<10^{-7}$ in either the FastEpistasis or BOOST analyses) were grouped by the number of SNPs that match a particular annotation category (**Match group**: none, either, or both SNPs matching). Annotation categories are described in the Supplemental Methods section. **N, total**: total number of interactions in each annotation match group. **N, rep (%)**: percentage of the total that were nominally replicated. **Enrich P**: enrichment test P-value (testing if there is a significant difference between percentages of nominally replicated interactions among the different annotation match groups; chi-square test or Fisher's exact test). **Min Int P**: among all interactions in the particular annotation match category, the P-value of the most significant interaction (A), and the P-value of the most significant nominally replicated interaction (R). **Thresh**: hypothetical significance threshold assuming that the search space was first trimmed to only include SNP pairs corresponding to the particular annotation match group. Green shading: enrichment P-value < 0.05. Cyan shading: a "Min Int P" value that is less than the respective "Thresh".
