# Supplementary material for: Exhaustive Genome-Wide Search for SNP-SNP Interactions Across 10 Human Diseases
Source: G3 (Bethesda). 2016 May 12;6(7):2043–50. doi: 10.1534/g3.116.028563 (PMC4938657; doi:10.1534/g3.116.028563)
Supplement: Supplemental Material [file supp_g3.116.028563_TableS26.pdf]

Table S-26. Enrichment analysis (diabetes, type 2).

Annotation	Match group	FastEpistasis / logistic regression					BOOST				
		N, total	N, rep. (%)	Enrich P	Min Int P	Thresh	N, total	N, rep.	Enrich P	Min Int P	Thresh
Exonic	None	3248	2.3	0.615	A: 1.02E-10<br>R: 3.11E-10	1.13E-12	3912	4.7	0.697	A: 2.33E-11<br>R: 2.69E-11	1.13E-12
	Either	40	2.5		A: 2.25E-09<br>R: 4.33E-09	6.41E-11	38	5.3		A: 7.22E-09<br>R: 7.22E-09	6.41E-11
	Both	0	0		A: N/A<br>R: N/A	1.46E-08	0	0		A: N/A<br>R: N/A	1.46E-08
Reg. 1,2, or 3	None	3042	2.4	0.695	A: 1.02E-10<br>R: 3.11E-10	1.20E-12	3627	4.8	0.496	A: 2.33E-11<br>R: 2.69E-11	1.20E-12
	Either	241	1.7		A: 2.30E-10<br>R: 2.76E-08	1.42E-11	317	3.5		A: 5.62E-10<br>R: 8.34E-09	1.42E-11
	Both	5	0		A: 4.79E-08<br>R: N/A	6.74E-10	6	0		A: 2.55E-09<br>R: N/A	6.74E-10
Disease EQTL	None	3253	2.3	0.566	A: 1.02E-10<br>R: 3.11E-10	1.13E-12	3901	4.7	0.727	A: 2.33E-11<br>R: 2.69E-11	1.13E-12
	Either	35	2.9		A: 4.48E-09<br>R: 7.68E-08	5.90E-11	49	2.0		A: 2.32E-09<br>R: 1.59E-08	5.90E-11
	Both	0	0		A: N/A<br>R: N/A	1.23E-08	0	0		A: N/A<br>R: N/A	1.23E-08
Disease gene	None	2610	2.3	0.833	A: 1.02E-10<br>R: 6.27E-09	1.41E-12	3136	4.6	0.052	A: 2.33E-11<br>R: 2.98E-10	1.41E-12
	Either	640	2.7		A: 1.76E-10<br>R: 3.11E-10	5.50E-12	763	4.5		A: 2.69E-11<br>R: 2.69E-11	5.50E-12
	Both	38	2.6		A: 5.29E-09<br>R: 3.02E-07	8.57E-11	51	11.8		A: 7.11E-10<br>R: 7.22E-09	8.57E-11
Any gene	None	1010	2.1	0.682	A: 1.12E-10<br>R: 6.27E-09	3.74E-12	1252	4.4	0.297	A: 2.33E-11<br>R: 6.40E-10	3.74E-12
	Either	1633	2.6		A: 1.76E-10<br>R: 3.20E-09	2.24E-12	1909	4.4		A: 2.69E-11<br>R: 2.69E-11	2.24E-12
	Both	645	2.2		A: 1.02E-10<br>R: 3.11E-10	5.35E-12	789	5.7		A: 2.78E-10<br>R: 4.80E-10	5.35E-12
Marginal	None	2857	2.2	0.439	A: 1.02E-10<br>R: 3.11E-10	1.26E-12	3486	4.5	0.275	A: 2.33E-11<br>R: 2.69E-11	1.26E-12
	Either	415	3.1		A: 3.26E-10<br>R: 1.30E-08	9.54E-12	445	5.6		A: 5.47E-11<br>R: 6.40E-10	9.54E-12
	Both	16	0		A: 1.45E-08<br>R: N/A	2.89E-10	19	10.5		A: 1.55E-09<br>R: 6.31E-08	2.89E-10

Followed-up interactions (interactions with $P<10^{-7}$ in either the FastEpistasis or BOOST analyses) were grouped by the number of SNPs that match a particular annotation category (**Match group**: none, either, or both SNPs matching). Annotation categories are described in the Supplemental Methods section. **N, total**: total number of interactions in each annotation match group. **N, rep (%)**: percentage of the total that were nominally replicated. **Enrich P**: enrichment test P-value (testing if there is a significant difference between percentages of nominally replicated interactions among the different annotation match groups; chi-square test or Fisher's exact test). **Min Int P**: among all interactions in the particular annotation match category, the P-value of the most significant interaction (A), and the P-value of the most significant nominally replicated interaction (R). **Thresh**: hypothetical significance threshold assuming that the search space was first trimmed to only include SNP pairs corresponding to the particular annotation match group. Green shading: enrichment P-value < 0.05. Cyan shading: a "Min Int P" value that is less than the respective "Thresh".
