# Supplementary material for: Exhaustive Genome-Wide Search for SNP-SNP Interactions Across 10 Human Diseases
Source: G3 (Bethesda). 2016 May 12;6(7):2043–50. doi: 10.1534/g3.116.028563 (PMC4938657; doi:10.1534/g3.116.028563)
Supplement: Supplemental Material [file supp_g3.116.028563_TableS27.pdf]

Table S-27. Enrichment analysis (dyslipidaemia).

Annotation	Match group	FastEpistasis / logistic regression					BOOST				
		N, total	N, rep. (%)	Enrich P	Min Int P	Thresh	N, total	N, rep.	Enrich P	Min Int P	Thresh
Exonic	None	3435	2.7	0.340	A: 1.09E-11<br>R: 1.69E-09	1.12E-12	3776	4.2	1.000	A: 2.80E-11<br>R: 2.84E-10	1.12E-12
	Either	45	4.4		A: 1.06E-09<br>R: 1.40E-08	6.33E-11	39	2.6		A: 4.57E-09<br>R: 1.65E-08	6.33E-11
	Both	0	0		A: N/A<br>R: N/A	1.44E-08	1	0		A: 1.07E-08<br>R: N/A	1.44E-08
Reg. 1,2, or 3	None	3222	2.6	0.208	A: 1.09E-11<br>R: 6.18E-09	1.19E-12	3542	4.4	0.304	A: 2.80E-11<br>R: 2.84E-10	1.19E-12
	Either	252	4.4		A: 9.85E-11<br>R: 1.69E-09	1.41E-11	271	2.6		A: 1.62E-09<br>R: 1.72E-08	1.41E-11
	Both	6	0		A: 3.13E-09<br>R: N/A	6.67E-10	3	0		A: 1.79E-08<br>R: N/A	6.67E-10
Disease EQTL	None	3477	2.7	0.078	A: 1.09E-11<br>R: 1.69E-09	1.10E-12	3811	4.2	1.000	A: 2.80E-11<br>R: 2.84E-10	1.10E-12
	Either	3	33.3		A: 8.93E-09<br>R: 8.93E-09	8.33E-10	5	0		A: 2.65E-08<br>R: N/A	8.33E-10
	Both	0	0		A: N/A<br>R: N/A	2.54E-06	0	0		A: N/A<br>R: N/A	2.54E-06
Disease gene	None	3440	2.7	0.290	A: 1.09E-11<br>R: 1.69E-09	1.11E-12	3768	4.2	1.000	A: 2.80E-11<br>R: 2.84E-10	1.11E-12
	Either	40	5.0		A: 4.33E-09<br>R: 8.93E-09	9.39E-11	48	4.2		A: 5.94E-09<br>R: 3.02E-08	9.39E-11
	Both	0	0		A: N/A<br>R: N/A	3.18E-08	0	0		A: N/A<br>R: N/A	3.18E-08
Any gene	None	1085	3.6	0.055	A: 1.90E-11<br>R: 8.48E-09	3.70E-12	1111	4.5	0.854	A: 2.80E-11<br>R: 8.70E-10	3.70E-12
	Either	1738	2.4		A: 9.85E-11<br>R: 1.69E-09	2.21E-12	1914	4.1		A: 5.43E-11<br>R: 2.84E-10	2.21E-12
	Both	657	1.8		A: 1.09E-11<br>R: 6.18E-09	5.30E-12	791	4.1		A: 9.98E-11<br>R: 9.13E-09	5.30E-12
Marginal	None	3124	2.4	0.022	A: 1.90E-11<br>R: 1.69E-09	1.25E-12	3394	4.1	0.309	A: 2.80E-11<br>R: 2.84E-10	1.25E-12
	Either	346	4.9		A: 1.09E-11<br>R: 6.17E-08	9.47E-12	405	5.4		A: 7.95E-11<br>R: 1.88E-09	9.47E-12
	Both	10	0		A: 1.01E-08<br>R: N/A	2.88E-10	17	0		A: 1.94E-08<br>R: N/A	2.88E-10

Followed-up interactions (interactions with $P<10^{-7}$ in either the FastEpistasis or BOOST analyses) were grouped by the number of SNPs that match a particular annotation category (**Match group**: none, either, or both SNPs matching). Annotation categories are described in the Supplemental Methods section. **N, total**: total number of interactions in each annotation match group. **N, rep (%)**: percentage of the total that were nominally replicated. **Enrich P**: enrichment test P-value (testing if there is a significant difference between percentages of nominally replicated interactions among the different annotation match groups; chi-square test or Fisher's exact test). **Min Int P**: among all interactions in the particular annotation match category, the P-value of the most significant interaction (A), and the P-value of the most significant nominally replicated interaction (R). **Thresh**: hypothetical significance threshold assuming that the search space was first trimmed to only include SNP pairs corresponding to the particular annotation match group. Green shading: enrichment P-value < 0.05. Cyan shading: a "Min Int P" value that is less than the respective "Thresh".
