# Supplementary material for: Exhaustive Genome-Wide Search for SNP-SNP Interactions Across 10 Human Diseases
Source: G3 (Bethesda). 2016 May 12;6(7):2043–50. doi: 10.1534/g3.116.028563 (PMC4938657; doi:10.1534/g3.116.028563)
Supplement: Supplemental Material [file supp_g3.116.028563_TableS28.pdf]

Table S-28. Enrichment analysis (hemorrhoids).

Annotation	Match group	FastEpistasis / logistic regression					BOOST				
		N, total	N, rep. (%)	Enrich P	Min Int P	Thresh	N, total	N, rep.	Enrich P	Min Int P	Thresh
Exonic	None	3348	2.8	1.000	A: 1.05E-11<br>R: 1.80E-09	1.13E-12	4013	5.2	0.358	A: 1.18E-11<br>R: 1.18E-11	1.13E-12
	Either	44	2.3		A: 2.08E-09<br>R: 7.00E-08	6.39E-11	53	7.6		A: 1.39E-09<br>R: 1.39E-09	6.39E-11
	Both	0	0		A: N/A<br>R: N/A	1.45E-08	0	0		A: N/A<br>R: N/A	1.45E-08
Reg. 1,2, or 3	None	3151	2.8	0.903	A: 1.05E-11<br>R: 2.71E-09	1.20E-12	3759	5.0	0.013	A: 1.18E-11<br>R: 1.18E-11	1.20E-12
	Either	235	2.6		A: 6.11E-10<br>R: 1.80E-09	1.42E-11	299	8.7		A: 1.05E-10<br>R: 1.17E-09	1.42E-11
	Both	6	0		A: 1.61E-08<br>R: N/A	6.74E-10	8	12.5		A: 6.03E-09<br>R: 3.04E-08	6.74E-10
Disease EQTL	None	3392	2.7	N/A	A: 1.05E-11<br>R: 1.80E-09	1.11E-12	4066	5.2	N/A	A: 1.18E-11<br>R: 1.18E-11	1.11E-12
	Either	0	0		A: N/A<br>R: N/A	2.77E-08	0	0		A: N/A<br>R: N/A	2.77E-08
	Both	0	0		A: N/A<br>R: N/A	3.33E-03	0	0		A: N/A<br>R: N/A	3.33E-03
Disease gene	None	3390	2.7	1.000	A: 1.05E-11<br>R: 1.80E-09	1.11E-12	4065	5.2	1.000	A: 1.18E-11<br>R: 1.18E-11	1.11E-12
	Either	2	0		A: 3.70E-08<br>R: N/A	2.11E-09	1	0		A: 5.44E-08<br>R: N/A	2.11E-09
	Both	0	0		A: N/A<br>R: N/A	1.62E-05	0	0		A: N/A<br>R: N/A	1.62E-05
Any gene	None	1074	2.0	0.126	A: 1.05E-11<br>R: 1.05E-08	3.73E-12	1201	4.6	0.459	A: 1.18E-11<br>R: 1.18E-11	3.73E-12
	Either	1658	3.3		A: 2.18E-11<br>R: 2.71E-09	2.23E-12	2074	5.5		A: 3.49E-11<br>R: 2.83E-10	2.23E-12
	Both	660	2.7		A: 5.85E-11<br>R: 1.80E-09	5.35E-12	791	5.7		A: 1.36E-10<br>R: 2.94E-09	5.35E-12
Marginal	None	3062	2.8	0.439	A: 1.05E-11<br>R: 1.80E-09	1.23E-12	3649	5.1	0.216	A: 1.18E-11<br>R: 1.18E-11	1.23E-12
	Either	326	1.8		A: 6.09E-10<br>R: 5.67E-09	1.13E-11	406	6.9		A: 5.04E-10<br>R: 2.94E-09	1.13E-11
	Both	4	0		A: 6.26E-08<br>R: N/A	4.13E-10	11	0		A: 8.79E-09<br>R: N/A	4.13E-10

Followed-up interactions (interactions with $P<10^{-7}$ in either the FastEpistasis or BOOST analyses) were grouped by the number of SNPs that match a particular annotation category (**Match group**: none, either, or both SNPs matching). Annotation categories are described in the Supplemental Methods section. **N, total**: total number of interactions in each annotation match group. **N, rep (%)**: percentage of the total that were nominally replicated. **Enrich P**: enrichment test P-value (testing if there is a significant difference between percentages of nominally replicated interactions among the different annotation match groups; chi-square test or Fisher's exact test). **Min Int P**: among all interactions in the particular annotation match category, the P-value of the most significant interaction (A), and the P-value of the most significant nominally replicated interaction (R). **Thresh**: hypothetical significance threshold assuming that the search space was first trimmed to only include SNP pairs corresponding to the particular annotation match group. Green shading: enrichment P-value < 0.05. Cyan shading: a "Min Int P" value that is less than the respective "Thresh".
