# Supplementary material for: Exhaustive Genome-Wide Search for SNP-SNP Interactions Across 10 Human Diseases
Source: G3 (Bethesda). 2016 May 12;6(7):2043–50. doi: 10.1534/g3.116.028563 (PMC4938657; doi:10.1534/g3.116.028563)
Supplement: Supplemental Material [file supp_g3.116.028563_TableS29.pdf]

Table S-29. Enrichment analysis (hypertensive disease).

Annotation	Match group	FastEpistasis / logistic regression					BOOST				
		N, total	N, rep. (%)	Enrich P	Min Int P	Thresh	N, total	N, rep.	Enrich P	Min Int P	Thresh
Exonic	None	3461	2.4	0.380	A: 1.41E-11<br>R: 2.20E-09	1.12E-12	3898	5.9	0.391	A: 1.09E-11<br>R: 2.51E-10	1.12E-12
	Either	55	3.6		A: 6.48E-10<br>R: 1.06E-06	6.33E-11	58	8.6		A: 1.22E-09<br>R: 1.22E-09	6.33E-11
	Both	0	0		A: N/A<br>R: N/A	1.43E-08	0	0		A: N/A<br>R: N/A	1.43E-08
Reg. 1,2, or 3	None	3254	2.3	0.281	A: 1.41E-11<br>R: 2.20E-09	1.19E-12	3638	5.9	0.652	A: 1.09E-11<br>R: 1.22E-09	1.19E-12
	Either	258	3.5		A: 2.78E-10<br>R: 7.87E-09	1.41E-11	311	6.8		A: 7.91E-11<br>R: 2.51E-10	1.41E-11
	Both	4	0		A: 8.61E-08<br>R: N/A	6.69E-10	7	0		A: 1.26E-08<br>R: N/A	6.69E-10
Disease EQTL	None	3489	2.4	0.135	A: 1.41E-11<br>R: 2.20E-09	1.11E-12	3929	6.0	0.404	A: 1.09E-11<br>R: 2.51E-10	1.11E-12
	Either	27	7.4		A: 2.27E-09<br>R: 1.06E-06	9.50E-11	27	0		A: 5.15E-09<br>R: N/A	9.50E-11
	Both	0	0		A: N/A<br>R: N/A	3.24E-08	0	0		A: N/A<br>R: N/A	3.24E-08
Disease gene	None	3171	2.3	0.333	A: 1.41E-11<br>R: 2.20E-09	1.22E-12	3587	5.8	0.400	A: 1.09E-11<br>R: 2.51E-10	1.22E-12
	Either	341	3.2		A: 2.69E-10<br>R: 4.23E-09	1.16E-11	361	7.2		A: 6.99E-11<br>R: 1.17E-08	1.16E-11
	Both	4	0		A: 5.26E-07<br>R: N/A	4.41E-10	8	12.5		A: 1.01E-08<br>R: 1.15E-08	4.41E-10
Any gene	None	1073	2.2	0.261	A: 1.41E-11<br>R: 2.20E-09	3.70E-12	1167	5.7	0.952	A: 1.41E-10<br>R: 5.71E-09	3.70E-12
	Either	1766	2.2		A: 2.64E-11<br>R: 4.18E-09	2.22E-12	1995	6.0		A: 6.99E-11<br>R: 2.51E-10	2.22E-12
	Both	677	3.3		A: 9.16E-11<br>R: 7.87E-09	5.31E-12	794	5.9		A: 1.09E-11<br>R: 1.96E-09	5.31E-12
Marginal	None	3038	2.1	1.36E-04	A: 1.41E-11<br>R: 2.20E-09	1.26E-12	3467	5.9	0.552	A: 1.09E-11<br>R: 2.51E-10	1.26E-12
	Either	459	3.5		A: 1.56E-10<br>R: 2.47E-08	8.75E-12	472	5.5		A: 2.26E-10<br>R: 7.70E-09	8.75E-12
	Both	19	15.8		A: 4.09E-09<br>R: 8.31E-07	2.42E-10	17	11.8		A: 1.18E-08<br>R: 1.86E-08	2.42E-10

Followed-up interactions (interactions with $P<10^{-7}$ in either the FastEpistasis or BOOST analyses) were grouped by the number of SNPs that match a particular annotation category (**Match group**: none, either, or both SNPs matching). Annotation categories are described in the Supplemental Methods section. **N, total**: total number of interactions in each annotation match group. **N, rep (%)**: percentage of the total that were nominally replicated. **Enrich P**: enrichment test P-value (testing if there is a significant difference between percentages of nominally replicated interactions among the different annotation match groups; chi-square test or Fisher's exact test). **Min Int P**: among all interactions in the particular annotation match category, the P-value of the most significant interaction (A), and the P-value of the most significant nominally replicated interaction (R). **Thresh**: hypothetical significance threshold assuming that the search space was first trimmed to only include SNP pairs corresponding to the particular annotation match group. Green shading: enrichment P-value < 0.05. Cyan shading: a "Min Int P" value that is less than the respective "Thresh".
