# Supplementary material for: Exhaustive Genome-Wide Search for SNP-SNP Interactions Across 10 Human Diseases
Source: G3 (Bethesda). 2016 May 12;6(7):2043–50. doi: 10.1534/g3.116.028563 (PMC4938657; doi:10.1534/g3.116.028563)
Supplement: Supplemental Material [file supp_g3.116.028563_TableS30.pdf]

Table S-30. Enrichment analysis (osteoarthritis).

Annotation	Match group	FastEpistasis / logistic regression					BOOST				
		N, total	N, rep. (%)	Enrich P	Min Int P	Thresh	N, total	N, rep.	Enrich P	Min Int P	Thresh
Exonic	None	3313	3.0	0.181	A: 1.86E-11<br>R: 1.33E-10	1.12E-12	3650	5.8	0.075	A: 1.85E-11<br>R: 2.78E-10	1.12E-12
	Either	48	6.3		A: 1.57E-09<br>R: 2.07E-07	6.35E-11	56	0		A: 1.54E-10<br>R: N/A	6.35E-11
	Both	0	0		A: N/A<br>R: N/A	1.44E-08	0	0		A: N/A<br>R: N/A	1.44E-08
Reg. 1,2, or 3	None	3118	3.1	1.000	A: 1.86E-11<br>R: 1.33E-10	1.20E-12	3425	5.8	0.541	A: 1.85E-11<br>R: 2.78E-10	1.20E-12
	Either	238	2.9		A: 4.22E-10<br>R: 3.88E-08	1.42E-11	271	4.4		A: 9.50E-10<br>R: 2.99E-08	1.42E-11
	Both	5	0		A: 5.82E-08<br>R: N/A	6.70E-10	10	10.0		A: 5.46E-09<br>R: 5.46E-09	6.70E-10
Disease EQTL	None	3354	3.1	1.000	A: 1.86E-11<br>R: 1.33E-10	1.11E-12	3697	5.7	0.412	A: 1.85E-11<br>R: 2.78E-10	1.11E-12
	Either	7	0		A: 3.57E-09<br>R: N/A	2.09E-10	9	11.1		A: 1.10E-08<br>R: 5.54E-08	2.09E-10
	Both	0	0		A: N/A<br>R: N/A	1.58E-07	0	0		A: N/A<br>R: N/A	1.58E-07
Disease gene	None	3315	3.1	0.651	A: 1.86E-11<br>R: 1.33E-10	1.12E-12	3652	5.7	0.235	A: 1.85E-11<br>R: 2.78E-10	1.12E-12
	Either	46	4.4		A: 2.26E-09<br>R: 2.40E-07	7.70E-11	54	9.3		A: 2.45E-09<br>R: 2.66E-08	7.70E-11
	Both	0	0		A: N/A<br>R: N/A	2.12E-08	0	0		A: N/A<br>R: N/A	2.12E-08
Any gene	None	1083	3.1	0.537	A: 8.15E-11<br>R: 1.33E-10	3.71E-12	1181	6.3	0.560	A: 6.12E-11<br>R: 2.78E-10	3.71E-12
	Either	1657	3.3		A: 2.57E-10<br>R: 4.40E-10	2.22E-12	1800	5.3		A: 1.85E-11<br>R: 8.63E-10	2.22E-12
	Both	621	2.4		A: 1.86E-11<br>R: 2.43E-09	5.32E-12	725	5.8		A: 1.38E-10<br>R: 1.16E-09	5.32E-12
Marginal	None	3006	3.0	0.625	A: 1.86E-11<br>R: 1.33E-10	1.23E-12	3328	5.8	0.731	A: 1.85E-11<br>R: 2.78E-10	1.23E-12
	Either	345	3.8		A: 2.24E-10<br>R: 9.83E-09	1.07E-11	366	4.9		A: 1.24E-10<br>R: 1.16E-09	1.07E-11
	Both	10	0		A: 2.34E-08<br>R: N/A	3.74E-10	12	8.3		A: 2.23E-09<br>R: 5.71E-08	3.74E-10

Followed-up interactions (interactions with $P<10^{-7}$ in either the FastEpistasis or BOOST analyses) were grouped by the number of SNPs that match a particular annotation category (**Match group**: none, either, or both SNPs matching). Annotation categories are described in the Supplemental Methods section. **N, total**: total number of interactions in each annotation match group. **N, rep (%)**: percentage of the total that were nominally replicated. **Enrich P**: enrichment test P-value (testing if there is a significant difference between percentages of nominally replicated interactions among the different annotation match groups; chi-square test or Fisher's exact test). **Min Int P**: among all interactions in the particular annotation match category, the P-value of the most significant interaction (A), and the P-value of the most significant nominally replicated interaction (R). **Thresh**: hypothetical significance threshold assuming that the search space was first trimmed to only include SNP pairs corresponding to the particular annotation match group. Green shading: enrichment P-value < 0.05. Cyan shading: a "Min Int P" value that is less than the respective "Thresh".
