# Supplementary material for: Exhaustive Genome-Wide Search for SNP-SNP Interactions Across 10 Human Diseases
Source: G3 (Bethesda). 2016 May 12;6(7):2043–50. doi: 10.1534/g3.116.028563 (PMC4938657; doi:10.1534/g3.116.028563)
Supplement: Supplemental Material [file supp_g3.116.028563_TableS31.pdf]

Table S-31A. BioGRID interactions (FastEpistasis with logistic regression), by condition.

Condition	SNP1				SNP2				Discovery				Replication			Rep.?	Gene1	Gene2
	RSID	Chr	A1	A0	RSID	Chr	A1	A0	FE, P	Unadj., P	Adj., OR	Adj., P	Unadj., P	Adj., OR	Adj, p			
Allergic rhinitis	None																	
Asthma	rs300572	4	A	G	rs2414444	15	C	T	4.78E-08	8.67E-08	0.86 (0.81, 0.91)	6.90E-08	7.82E-01	0.99 (0.85, 1.15)	8.75E-01	No	SPRY1	NEDD4
Cardiac disease	None																	
Depression	rs3177954	14	C	T	rs179255	14	A	G	6.72E-08	8.22E-08	0.83 (0.77, 0.89)	9.30E-08	4.07E-01	1.08 (0.91, 1.28)	3.61E-01	No	SALL2	TSHR
Dermatophytosis	None																	
Diabetes, type 2	None																	
Dyslipidaemia	None																	
Hemorrhoids	rs996174	3	C	T	rs17064660	3	A	G	9.98E-08	1.20E-07	0.83 (0.77, 0.89)	1.27E-07	6.72E-01	0.96 (0.80, 1.15)	6.30E-01	No	FHIT	FHIT
Hypertensive dis.	None																	
Osteoarthritis	None																	

Interactions were first analyzed with FastEpistasis. Each interaction with FastEpistasis P < 10-7 was assessed to determine if it met the criteria of being a "BioGRID interaction" (see the Supplemental Methods section). All such interactions are listed here and were subjected to analysis with logistic regression. **Chr**: chromosome number. **A1**: non-referent allele. **A0**: referent allele. **FE, P**: P-value from the Fast Epistasis analysis. **Unadj., P**: P-value from the unadjusted logistic regression analysis. **Adj., OR**: interaction odds ratio and 95% confidence interval, from the adjusted logistic regression analysis. **Adj., P**: P-value from the adjusted logistic regression analysis. All "adjusted" analyses were adjusted for the first two principal components, birth year category, and sex. **Rep.?** Whether or not the interaction was nominally replicated. **Gene1, Gene2**: gene assigned to SNP1 and SNP2, respectively. **None**: for these conditions, there were no interactions with FastEpistasis P < 10-7 that could be considered a BioGRID interaction.

Table S-31B. BioGRID interactions (BOOST), by condition.

Condition	SNP1				SNP2				Discovery P	Replication P	Rep.?	Gene1	Gene2
	RSID	Chr	A1	A0	RSID	Chr	A1	A0					
Allergic rhinitis	None												
Asthma	rs300572	4	A	G	rs2414444	15	C	T	1.25E-08	6.35E-01	No	SPRY1	NEDD4
Cardiac disease	None												
Depression	None												
Dermatophytosis	None												
Diabetes, type 2	rs7933204	11	G	A	rs2388067	13	T	G	6.72E-08	5.77E-01	No	ROBO3	MTUS2
Dyslipidaemia	None												
Hemorrhoids	None												
Hypertensive dis.	None												
Osteoarthritis	None												

Interactions were first analyzed with BOOST. Each interaction with BOOST P < 10-7 was assessed to determine if it met the criteria of being a "BioGRID interaction" (see the Supplemental Methods section). All such interactions are listed here. **Chr**: chromosome number. **A1**: non-referent allele. **A0**: referent allele. **Discovery P**: P-value from the discovery BOOST analysis. **Replication P**: P-value from the replication BOOST analysis. **Rep.?** Whether or not the interaction was nominally replicated. **Gene1, Gene2**: gene assigned to SNP1 and SNP2, respectively. **None**: for these conditions, there were no interactions with BOOST P < 10-7 that could be considered a BioGRID interaction.
