# Supplementary material for: Exhaustive Genome-Wide Search for SNP-SNP Interactions Across 10 Human Diseases
Source: G3 (Bethesda). 2016 May 12;6(7):2043–50. doi: 10.1534/g3.116.028563 (PMC4938657; doi:10.1534/g3.116.028563)
Supplement: Supplemental Material [file supp_g3.116.028563_FileS2.pdf]

Supplement for:

Exhaustive genome-wide search for SNP-SNP interactions across ten human diseases

William Murk, PhD* and Andrew T. DeWan, PhD*,†

*Department of Chronic Disease Epidemiology, Yale School of Public Health, 60 College St., New Haven, CT 06510, USA.

†Corresponding author. Email: andrew.dewan@yale.edu. Tel: 203-785-3528.

SUPPLEMENTAL METHODS

SNP annotation

A SNP was assigned to a gene if it was inside, or was located within 5 kilobases (kb) upstream or downstream of, a gene transcript. This was done using Annovar (2015Mar22 version; http://annovar.openbioinformatics.org) (Wang *et al.* 2010) and the UCSC Known Gene database (Hsu *et al.* 2006). SNPs were annotated based on the following categories: "exonic"; "regulatory"; "disease-gene"; "disease-eQTL"; "any-gene "; and "marginal". An "exonic" (EX) SNP was one that results in a frameshift, a non-synonymous substitution, or a stopgain (determined using Annovar). A "regulatory" (R) SNP was one that was annotated in RegulomeDB version 1.1 (Boyle *et al.* 2012) as having a score of 1, 2, or 3 (i.e., at least having evidence of tissue factor binding and a binding motif). A "disease-gene" (D) SNP was one that was assigned to a gene that has been previously associated with the particular condition of interest. The Genetic Association Database (09/01/2014 data freeze) (Becker *et al.* 2004) was used to identify genes previously associated with the conditions, using database search terms described in **Table S-5**. For dermatophytosis and hemorrhoids, the DisGenNET database was also searched (Pinero *et al.* 2015). A "disease-eQTL" (Q) SNP was a SNP that was itself, or was located within 1 kilobase upstream or downstream of, a SNP that has been annotated as being an eQTL (expression quantitative trait locus) affecting a gene that has been previously associated with the particular condition of interest. These were identified using eQTL annotations in RegulomeDB and the genes found via the Genetic Association Database. An "any-gene" (G) SNP is a SNP that was assigned to any gene. A "marginal" (M) SNP was a SNP with a marginal effect P-value < 0.05 in the discovery dataset for the respective condition, after adjusting for birth year category, age, and the first two principal components. The kgAlias table from the UCSC Table Browser (Karolchik *et al.* 2004) was used to account for gene names aliases when matching between gene names of the different annotation source databases. The numbers of SNPs within each annotation category are listed in **Table S-6**.

Annotation of BioGRID interactions

All followed-up interactions (interaction P < 10^{-7} from either the FastEpistasis or BOOST analyses) were assessed to determine if the two SNPs of each interaction were located in genes that are known to interact with one another, as reported in the BioGRID database (http://thebiogrid.org) (Stark *et al.* 2006). Specifically, we obtained the BioGRID database for *Homo sapiens* (version 3.4.129), which is a curated list of pairs of genes (or their protein products) that have been previously reported to interact genetically or physically. If, in a followed-up interaction from the current study, one SNP was located inside, or within 1 kb of, the transcribed region of one gene of a BioGRID pair, and the other SNP was located inside, or within 1 kb of, the transcribed region of the other gene of the same BioGRID pair, then that interaction was flagged as a "BioGRID interaction".

Enrichment analysis

All followed-up interactions (i.e., those with interaction P < 10^{-7} from either the FastEpistasis or BOOST analyses) were grouped based on how many of their participating SNPs matched a particular annotation category (i.e., three possible match groups: neither SNP, only one SNP, or both SNPs matching). To examine if interactions were enriched with SNPs of a particular annotation category, statistical tests were performed to determine if there was a significant difference between the proportion of interactions that were nominally replicated and the match group of the interaction (i.e., hypothesizing that interactions with more matches are more likely to be replicated). Specifically, for every possible condition and every possible annotation category, a 3 by 2 contingency table was constructed, where the rows were the match group and the columns were the replication result (yes or no); the cells of the tables contained interaction counts. These tables were then analyzed using chi-square tests or Fishers' exact tests (the latter were used in situations where 25% or more of the table cells had expected counts that were less than five).

Prior to these enrichment analyses, the lists of followed-up interactions were trimmed to remove non-independent observations (i.e., to remove interactions where the SNPs may have been in linkage disequilibrium with the SNPs of another interaction). This was done by grouping interactions by proximity, based on distances of less than 100 kb between SNPs. For example, if SNP-A of Interaction-1 was located within 100 kb of either SNP-A or SNP-B of Interaction-2, *and* SNP-B of Interaction-1 was located within 100 kb of either SNP-A or SNP-B of Interaction-2, then the

two interactions were grouped (more than two interactions could be grouped together). Among each group, only one interaction was selected for inclusion (this was the interaction with the most significant interaction P-value in the discovery dataset). For each enrichment analysis, approximately 3,000 to 3,500 interactions were ultimately included. This trimming was only performed for enrichment analyses; the overall genome-wide interaction results (described in other sections of this manuscript) were not trimmed in this manner.

Since there were ten conditions, six annotation categories, and two analytical methods evaluated (FastEpistasis and BOOST), a total of 120 enrichment tests were performed.

Estimation of phenotypic variance explained by additive genetic variance of the included SNPs

GCTA version 1.24 (Yang *et al.* 2011) was used to estimate the proportion of phenotypic variance explained by additive genetic variance, for each studied condition. These estimates were restricted to the SNPs that were ultimately included in the interaction analyses (approximately 300,000 SNPs; see **Table S-3** for precise numbers). In addition, these estimates were calculated within subsets of the total sample. Specifically, approximately 4,000 cases and 4,000 controls were randomly selected for GCTA analysis, from the subjects included in the interaction analyses. Default analytical options were used, including use of the "AI-REML" algorithm (http://cnsgenomics.com/software/gcta/GCTA_UserManual_v1.24.pdf). The first two principal components were included as covariates. Disease prevalence estimates were the same as those that are specified in **Table S-7**. Phenotypic variance explained by the SNPs is expressed as V(G) / Vp_L, which is the genetic variance captured by the SNPs over the phenotypic variance, transformed to the underlying liability scale.

Power estimation

Power was estimated analytically using Quanto version 1.2 ((http://biostats.usc.edu/software) and empirically using epiSIM version 1.10 (Shang *et al.* 2011) (**Table S-7** and **S-8**). Quanto was used to estimate the power to detect an interaction using logistic regression, where each SNP has a MAF of 0.15, with no main effects, and an interaction odds ratio of either 1.25 or 1.50. The population prevalence of each condition was assumed to be equal to the prevalence of the condition in the overall GERA dataset, rounded to the nearest 5%.

Data simulated with epiSIM were used to estimate the power to detect an interaction via FastEpistasis and BOOST, assuming an interaction involving the model shown in **Table S-9** (derived from (Shang *et al.* 2011)). For each disease condition, data were simulated using three different relative penetrances (f = 0.15, 0.20, and 0.25), for case-control counts corresponding to the respective discovery and replication datasets. Baseline penetrance was determined such that the overall prevalence of disease approximately equaled the prevalence of the condition in the overall GERA dataset, rounded to the nearest 5%. For every combination of variable (condition, relative penetrance, discovery/replication), 100 datasets were generated. Power was calculated as the percentage of tests whose interaction P-value exceeded the specified type I error threshold.

Supplemental Methods References

Becker, K. G., K. C. Barnes, T. J. Bright and S. A. Wang, 2004 The genetic association database. Nat Genet 36**:** 431-432.

Boyle, A. P., E. L. Hong, M. Hariharan, Y. Cheng, M. A. Schaub *et al.*, 2012 Annotation of functional variation in personal genomes using RegulomeDB. Genome Res 22**:** 1790-1797.

Hsu, F., W. J. Kent, H. Clawson, R. M. Kuhn, M. Diekhans *et al.*, 2006 The UCSC Known Genes. Bioinformatics 22**:** 1036-1046.

Karolchik, D., A. S. Hinrichs, T. S. Furey, K. M. Roskin, C. W. Sugnet *et al.*, 2004 The UCSC Table Browser data retrieval tool. Nucleic Acids Res 32**:** D493-496.

Pinero, J., N. Queralt-Rosinach, A. Bravo, J. Deu-Pons, A. Bauer-Mehren *et al.*, 2015 DisGeNET: a discovery platform for the dynamical exploration of human diseases and their genes. Database (Oxford) 2015**:** bav028.

Shang, J., J. Zhang, Y. Sun, D. Liu, D. Ye *et al.*, 2011 Performance analysis of novel methods for detecting epistasis. BMC Bioinformatics 12**:** 475.

Stark, C., B. J. Breitkreutz, T. Reguly, L. Boucher, A. Breitkreutz *et al.*, 2006 BioGRID: a general repository for interaction datasets. Nucleic Acids Res 34**:** D535-539.

Wang, K., M. Li and H. Hakonarson, 2010 ANNOVAR: functional annotation of genetic variants from high-throughput sequencing data. Nucleic Acids Res 38**:** e164.

Yang, J., S. H. Lee, M. E. Goddard and P. M. Visscher, 2011 GCTA: a tool for genome-wide complex trait analysis. Am J Hum Genet 88**:** 76-82.
