# Supplementary material for: Exhaustive Genome-Wide Search for SNP-SNP Interactions Across 10 Human Diseases
Source: G3 (Bethesda). 2016 May 12;6(7):2043–50. doi: 10.1534/g3.116.028563 (PMC4938657; doi:10.1534/g3.116.028563)
Supplement: Supplemental Material [file supp_g3.116.028563_TableS1.pdf]

Table S-1. Subject quality control.

Inclusion/exclusion step	N, subjects
Initially available (genotyped on the EUR chip)	62,318
Excluded, ambiguous SNP-estimated sex	55
Excluded, subject call rate < 95%	0
Excluded, relatedness; pi-hat >= 0.20	16,457
Excluded, race not reported as "white" and/or European principal components not available	398
Excluded, PCA outliers	237
Remaining for inclusion in disease-specific analyses	45,171

Quality control steps were applied sequentially, in the order indicated from top to bottom.
