# Supplementary material for: Exhaustive Genome-Wide Search for SNP-SNP Interactions Across 10 Human Diseases
Source: G3 (Bethesda). 2016 May 12;6(7):2043–50. doi: 10.1534/g3.116.028563 (PMC4938657; doi:10.1534/g3.116.028563)
Supplement: Supplemental Material [file supp_g3.116.028563_TableS2.pdf]

Table S-2. Disease-specific subject counts.

Condition	N, subjects available, post-QC	Minimum allowed estimated age of controls	N, excluded, controls below minimum estimated age	Final subject counts			
				Discovery		Replication	
				N, Cases	N, Controls	N, Cases	N, Controls
Allergic rhinitis	45,171	18 years	0	10,258	30,933	976	3,004
Asthma	45,171	18 years	0	6,486	34,669	988	3,028
Cardiac disease	45,171	34 years	1,106	11,069	28,979	1,004	3,013
Depression	45,171	24 years	215	4,824	36,162	978	2,992
Dermatophytosis	45,171	18 years	0	5,163	36,083	989	2,936
Diabetes, type 2	45,171	34 years	1,106	4,563	35,573	986	2,943
Dyslipidaemia	45,171	34 years	1,106	23,061	17,021	986	2,997
Hemorrhoids	45,171	29 years	493	6,199	34,356	1,006	3,117
Hypertensive disease	45,171	34 years	1,106	21,713	18,332	984	3,036
Osteoarthritis	45,171	39 years	2,193	15,454	23,578	961	2,985
