# Supplementary material for: Exhaustive Genome-Wide Search for SNP-SNP Interactions Across 10 Human Diseases
Source: G3 (Bethesda). 2016 May 12;6(7):2043–50. doi: 10.1534/g3.116.028563 (PMC4938657; doi:10.1534/g3.116.028563)
Supplement: Supplemental Material [file supp_g3.116.028563_TableS3.pdf]

**Table S-3. SNP quality control.**

| Condition | N, SNPs initially available | N, Excluded, could not map to hg19 | N, Excluded, SNP call rate < 98% (overall) | N, Excluded, MAF < 0.15 (overall) | N, Excluded, non-autosomal | N, Excluded, call rate < 98% in cases and/or controls | N, Excluded, HWE $p < 10^{-5}$ in controls | N, remaining for final analysis |
|---|---|---|---|---|---|---|---|---|
| Allergic rhinitis | 670,176 | 464 | 36,300 | 321,745 | 7,316 | 488 | 3,643 | 300,220 |
| Asthma | 670,176 | 464 | 36,300 | 321,745 | 7,316 | 305 | 3,385 | 300,661 |
| Cardiac disease | 670,176 | 464 | 36,300 | 321,745 | 7,316 | 325 | 3,210 | 300,816 |
| Depression | 670,176 | 464 | 36,300 | 321,745 | 7,316 | 463 | 3,694 | 300,194 |
| Dermatophytosis | 670,176 | 464 | 36,300 | 321,745 | 7,316 | 414 | 3,648 | 300,289 |
| Diabetes, type 2 | 670,176 | 464 | 36,300 | 321,745 | 7,316 | 392 | 3,673 | 300,286 |
| Dyslipidaemia | 670,176 | 464 | 36,300 | 321,745 | 7,316 | 267 | 2,267 | 301,817 |
| Hemorrhoids | 670,176 | 464 | 36,300 | 321,745 | 7,316 | 348 | 3,583 | 300,420 |
| Hypertensive disease | 670,176 | 464 | 36,300 | 321,745 | 7,316 | 336 | 2,429 | 301,586 |
| Osteoarthritis | 670,176 | 464 | 36,300 | 321,745 | 7,316 | 259 | 2,788 | 301,304 |

N refers to number of SNPs. Quality control steps were applied sequentially, in the order indicated from left to right.
