# Supplementary material for: Exhaustive Genome-Wide Search for SNP-SNP Interactions Across 10 Human Diseases
Source: G3 (Bethesda). 2016 May 12;6(7):2043–50. doi: 10.1534/g3.116.028563 (PMC4938657; doi:10.1534/g3.116.028563)
Supplement: Supplemental Material [file supp_g3.116.028563_TableS4.pdf]

Table S-4. Genomic inflation, marginal effects.

Condition	Dataset	λ (genomic inflation factor)		
		No adjustment	Adjustment for PC1, PC2	Adjustment for PC1, PC2, sex, age
Allergic rhinitis	Discovery	1.03	1.03	1.03
	Replication	1.01	1.01	1.01
Asthma	Discovery	1.03	1.03	1.03
	Replication	1.02	1.02	1.02
Cardiac disease	Discovery	1.14	1.02	1.02
	Replication	1.00	1.00	1.00
Depression	Discovery	1.04	1.01	1.02
	Replication	1.01	1.01	1.01
Dermatophytosis	Discovery	1.23	1.02	1.02
	Replication	1.04	1.00	1.00
Diabetes, type 2	Discovery	1.08	1.08	1.09
	Replication	1.02	1.01	1.02
Dyslipidaemia	Discovery	1.11	1.07	1.08
	Replication	1.04	1.00	1.02
Hemorrhoids	Discovery	1.04	1.01	1.01
	Replication	1.03	1.00	1.00
Hypertensive disease	Discovery	1.30	1.10	1.13
	Replication	1.01	1.01	1.00
Osteoarthritis	Discovery	1.03	1.02	1.03
	Replication	1.01	1.01	1.01

Red shading indicates λ > 1.05.
