# Supplementary material for: Exhaustive Genome-Wide Search for SNP-SNP Interactions Across 10 Human Diseases
Source: G3 (Bethesda). 2016 May 12;6(7):2043–50. doi: 10.1534/g3.116.028563 (PMC4938657; doi:10.1534/g3.116.028563)
Supplement: Supplemental Material [file supp_g3.116.028563_TableS5.pdf]

Table S-5. Database search for previously known disease-related candidate genes

**Condition**	**GAD search term(s)**	**N, unique gene symbols found**
Allergic rhinitis	“rhinitis”	122
Asthma	“asthma”	762
Cardiac disease	“heart failure”, “heart disease”, “myocardial infarc”, “angina”, “cardiomyopathy”, “tachycardia”, “aneurysm”, “cardiac arrest”	1,579
Depression	“depression”, “depressive”	350
Dermatophytosis[1]	“dermatophytosis”	3
Diabetes, type 2	“type 2 diabetes”, “type ii diabetes”, “diabetes, type 2”, “diabetes type 2”, “diabetes mellitus, type 2”, “diabetes mellitus type 2”, “diabetes mellitus, type ii”, “diabetes mellitus type ii”	3,001
Dyslipidaemia	“dyslipid”, “hyperlipid”, “hypertriglycer”	150
Hemorrhoids[1]	“hemorrhoid”	7
Hypertensive disease	“hypertens”	971
Osteoarthritis	“osteoarthritis”	150

GAD: Genetic Association Database. Searches were not case-sensitive. Search terms were allowed to be sub-strings of larger strings in GAD (for example, “hypertens” would return entries including either “hypertension” and “hypertensive”). **1**: Zero (dermatophytosis) or only one (hemorrhoids) resulting gene symbol found in GAD. The DisGenNET database (http://www.disgenet.org/) was also searched for these two conditions, using the exact condition names as the search terms. This resulted in three (dermatophytosis) and six (hemorrhoids) additional gene symbols for these conditions.
