# Supplementary material for: Exhaustive Genome-Wide Search for SNP-SNP Interactions Across 10 Human Diseases
Source: G3 (Bethesda). 2016 May 12;6(7):2043–50. doi: 10.1534/g3.116.028563 (PMC4938657; doi:10.1534/g3.116.028563)
Supplement: Supplemental Material [file supp_g3.116.028563_TableS6.pdf]

Table S-6. SNP annotations, by category.

Condition	N, Total	N, any gene (G)	N, exonic (EX)	N, by RegulomeDB score				N, by prior disease relevance type		N, marginal P < 0.05 (M)
				1	2	3	1,2, or 3 (R)	Gene (D)	eQTL (Q)	
Allergic rhinitis	300,220	136,829	2,629	3,805	4,375	4,007	12,187	851	851	16,260
Asthma	300,661	136,642	2,628	3,797	4,361	4,008	12,166	12,845	1,827	16,987
Cardiac disease	300,816	136,892	2,628	3,804	4,370	4,015	12,189	28,872	2,341	16,284
Depression	300,194	136,619	2,627	3,799	4,364	4,006	12,169	6,351	303	15,566
Dermatophytosis	300,289	136,680	2,627	3,801	4,366	4,008	12,175	26	6	16,090
Diabetes, type 2	300,286	136,667	2,622	3,803	4,368	4,012	12,183	34,168	2,848	18,613
Dyslipidaemia	301,817	137,356	2,640	3,827	4,385	4,031	12,243	1,775	199	18,637
Hemorrhoids	300,420	136,704	2,629	3,800	4,364	4,015	12,179	79	6	15,560
Hypertensive disease	301,586	137,247	2,644	3,819	4,384	4,026	12,229	15,054	1,756	20,319
Osteoarthritis	301,304	137,149	2,635	3,820	4,378	4,019	12,217	2,170	797	16,345

N refers to number of SNPs. The categories (except the RegulomeDB sub-categories of 1, 2, and 3) are not mutually exclusive.
